# Growth of sulfate-reducing *Desulfobacterota* and *Bacillota* at periodic oxygen stress of 50% air-O_2_ saturation

**DOI:** 10.1186/s40168-024-01909-7

**Published:** 2024-10-04

**Authors:** Stefan Dyksma, Michael Pester

**Affiliations:** 1 Department of Microorganisms, Leibniz Institute DSMZ–German Collection of Microorganisms and Cell Cultures, Braunschweig, Germany; 2 Technical University of Braunschweig, Institute of Microbiology, Braunschweig, Germany

**Keywords:** Sulfur cycle, Sulfate-reducing bacteria, Bioreactor, Oxygen defense, Reactive oxygen species, Oxic-anoxic transition zone

## Abstract

**Background:**

Sulfate-reducing bacteria (SRB) are frequently encountered in anoxic-to-oxic transition zones, where they are transiently exposed to microoxic or even oxic conditions on a regular basis. This can be marine tidal sediments, microbial mats, and freshwater wetlands like peatlands. In the latter, a cryptic but highly active sulfur cycle supports their anaerobic activity. Here, we aimed for a better understanding of how SRB responds to periodically fluctuating redox regimes.

**Results:**

To mimic these fluctuating redox conditions, a bioreactor was inoculated with peat soil supporting cryptic sulfur cycling and consecutively exposed to oxic (one week) and anoxic (four weeks) phases over a period of > 200 days. SRB affiliated to the genus *Desulfosporosinus* (*Bacillota*) and the families *Syntrophobacteraceae*, *Desulfomonilaceae*, *Desulfocapsaceae*, and *Desulfovibrionaceae* (*Desulfobacterota*) successively established growing populations (up to 2.9% relative abundance) despite weekly periods of oxygen exposures at 133 µM (50% air saturation). Adaptation mechanisms were analyzed by genome-centric metatranscriptomics. Despite a global drop in gene expression during oxic phases, the perpetuation of gene expression for energy metabolism was observed for all SRBs. The transcriptional response pattern for oxygen resistance was differentiated across individual SRBs, indicating different adaptation strategies. Most SRB transcribed differing sets of genes for oxygen consumption, reactive oxygen species detoxification, and repair of oxidized proteins as a response to the periodical redox switch from anoxic to oxic conditions. Noteworthy, a *Desulfosporosinus*, a *Desulfovibrionaceaea*, and a *Desulfocapsaceaea* representative maintained high transcript levels of genes encoding oxygen defense proteins even under anoxic conditions, while representing dominant SRB populations after half a year of bioreactor operation.

**Conclusions:**

In situ-relevant peatland SRB established large populations despite periodic one-week oxygen levels that are one order of magnitude higher than known to be tolerated by pure cultures of SRB. The observed decrease in gene expression regulation may be key to withstand periodically occurring changes in redox regimes in these otherwise strictly anaerobic microorganisms. Our study provides important insights into the stress response of SRB that drives sulfur cycling at oxic-anoxic interphases.

Video Abstract

**Supplementary Information:**

The online version contains supplementary material available at 10.1186/s40168-024-01909-7.

## Introduction

Sulfate-reducing bacteria (SRB) have relevance in driving the global sulfur cycle [1] and share a common enzyme repertoire to carry out dissimilatory sulfate reduction. This sulfate reduction pathway encompasses the enzymes sulfate adenylyltransferase (Sat), adenylyl phosphosulfate reductase (AprAB), dissimilatory (bi)sulfite reductase (DsrAB), and the sulfide-releasing DsrC. The complexes QmoAB(C) and DsrMK(JOP) complement the pathway by transferring reducing equivalents towards AprAB and DsrC, respectively [1]. Furthermore, DsrD serves as an allosteric activator of DsrAB [2] and *dsrT* encodes a protein with an unknown function that is typically encoded on the *dsr* operon as well [3]. Due to the sensitivity of sulfate reduction pathway enzymes towards oxygen [4], most SRBs are characterized as strictly anaerobic microorganisms [3, 5]. However, they are often encountered in natural environments where oxic conditions can temporarily or periodically exist, e.g., in marine tidal sediments, microbial mats, and freshwater wetlands like peatlands [3]. Oxygenation impairs the metabolism of strictly anaerobic microorganisms generally by two mechanisms. First, molecular oxygen can directly disrupt enzyme activity, e.g., by the oxidation of catalytic metal centers with a low redox potential. Second, endogenous and environmental reactive oxygen species (ROS) cause oxidative stress due to damage to biomolecules like proteins and nucleic acids [6].

Anaerobes including the SRB have evolved mechanisms to tolerate oxygen and resulting oxidative stress to some extent. Oxygen reduction can be facilitated in the cytoplasm by the bifunctional rubredoxin:oxygen oxidoreductase/nitric oxide reductase (Roo/NorV) [7, 8]. Alternatively, this can be achieved by membrane-bound oxygen reductases such as the heme copper cytochrome *c* oxidase or *bd*-type oxidase (CydAB). The latter is widely distributed in SRB and known to play an important role in oxygen consumption as a defense mechanism [9, 10]. As a consequence of oxygenation, reactive oxygen species like superoxide, peroxides, and hydroxyl radicals are produced endogenously as metabolic byproducts. Extracellular superoxide and peroxide formation are also possible in environments where hydrogen sulfide, the end product of sulfate reduction, can react with oxygen [6]. To cope with ROS, many SRBs possess a variety of defense mechanisms that are also shared by aerobes [3, 11]. Superoxide can be disproportionated by superoxide dismutase (Sod) to molecular oxygen and hydrogen peroxide or reduced without the formation of oxygen by superoxide reductase (Sor)/desulfoferredoxin (Dfx) involving the electron transfer protein rubredoxin (Rub). Several proteins can be important for the removal of peroxides, which comprise oxygen-forming catalase (KatA), catalase-peroxidase (KatG), alkyl hydroperoxide reductase (Ahp), and rubrerythrin/reverse rubrerythrin (Rbr/revRbr) [12–17].

Repair of proteins damaged by oxidative stress is crucial to restore metabolic function [18–20]. Molecular chaperones participate in protein folding and refolding as well as in the prevention of aggregation and disaggregation [21], thus keeping proteins in their native state. The ClpB-DnaK chaperone system, which is assisted by other chaperones such as HtpG and DnaJ [22], is used for ATP-dependent refolding of insoluble protein aggregates [23]. Similarly, GroLS can bind to unfolded proteins enabling refolding, and has been associated with oxidative stress in bacteria [24, 25]. Thioredoxin (TrxA) and thioredoxin reductase (TrxB) can participate in various cellular functions [26] and together with peptide methionine sulfoxide reductase (MsrA) is also key for the repair of oxidized proteins [18–20, 27, 28]. In addition, SRB can avoid getting in contact with oxygen by behavioral strategies such as self-aggregation and aggregate formation with oxygen-scavenging microorganisms or migration to anoxic zones [11, 29].

Peatlands store up to one-third of the total terrestrial organic carbon [30, 31]. At the same time, they are well-known sources of the strong greenhouse gas methane [32, 33]. Typical peatlands are permanently or periodically water-saturated soils, which leads to steep gradients in redox conditions varying in space and time [30, 34–36]. Water table fluctuations during drying and rewetting regularly shift the oxic-anoxic interphase, with seasonal variability expected to increase due to climate change [37–39]. In these dynamic and largely anoxic environments, the peat soil microbiome drives complex biogeochemical cycling linked to organic carbon mineralization [40–43]. Sulfate concentrations in peatlands are typically in the lower micromolar range. However, efficient sulfate reduction coupled with rapid re-oxidation of reduced sulfur species sustains a cryptic sulfur cycle in peatlands and allows sulfate reduction rates (SRR) that can be as high as SRR in marine environments where sulfate is abundant [36]. As SRB compete with methanogenic *Archaea* for their common substrates such as acetate and molecular hydrogen (H_2_), they considerably attenuate methane emissions from these globally important ecosystems [44–46].

SRB that affiliate with the *Syntrophobacteraceae*, *Desulfomonilaceae, Desulfovibrionaceae*, and *Desulfosporosinus* are regularly identified in peat soils [34, 46–49]. Their abundance is often below the arbitrarily specified border of 0.1% relative abundance, which defines the rare biosphere [50]. Evidence is emerging that low-abundance species are not just dormant microorganisms providing a seed bank to become active and abundant under favorable environmental conditions, but can rather be hidden drivers of microbiome and ecosystem functioning [51, 52]. For example, low-abundance *Desulfosporosinus* representatives were suggested as keystone species in peat soil of an acidic fen, being responsible for a substantial fraction of sulfate reduction and carbon degradation [53]. In this fen, *Desulfosporosinus* maintained a stable but low-abundance population while showing increased metabolic activity under prolonged favorable sulfate-reducing conditions [46, 54].

To understand how peatland SRBs cope with periodically fluctuating redox conditions they encounter in their natural habitat, we incubated peat soil in a continuous bioreactor setting under alternating oxic and anoxic/sulfate-reducing conditions. The microbial response was followed using 16S rRNA gene amplicon sequencing and genome-centric metatranscriptomics. SRB of the *Desulfobacterota* and *Bacillota* not only survived a periodical exposure to 50% air-O_2_ saturation for 1 week but were even enriched during continuous cultivation and their transcriptional profiles revealed different oxygen defense strategies.

## Results and discussion

### Distinct peatland SRB is established under fluctuating oxic-anoxic conditions

**Fig. 1:**
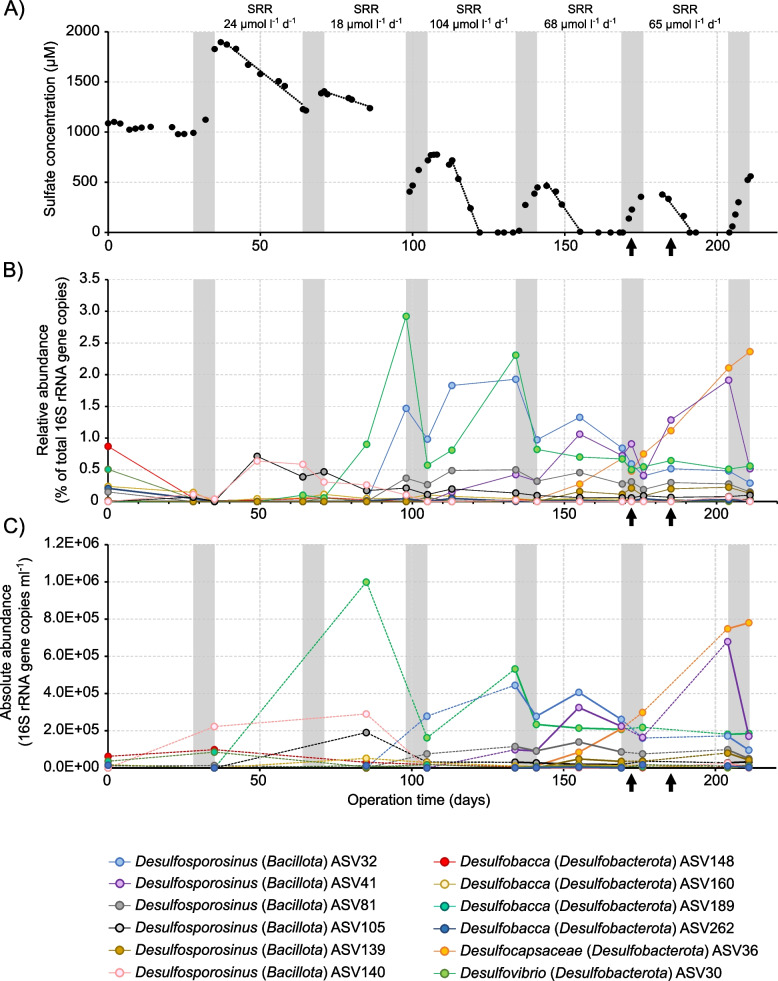
Bioreactor performance and enrichment of sulfate-reducing bacteria (SRB). Sulfate concentrations during bioreactor operation and sulfate reduction rates (**A**). Parts of the data on sulfate concentrations and sulfate reduction rates have been published elsewhere [55] and are reproduced here to contextualize SRB-community dynamics and sulfate reduction activity. Relative abundance of amplicon sequence variants (ASVs) affiliating with SRB (**B**). Only ASVs with a relative abundance ≥ 0.1% are shown. ASVs were determined by 16S rRNA gene amplicon sequencing. White and grey shaded areas represent anoxic (0% air-O_2_ saturation) and oxic (50% air-O_2_ saturation) conditions, respectively. Estimated absolute abundance of 16S rRNA gene copies of SRB-ASVs (**C**) as determined by quantitative PCR (qPCR) targeting total *Bacteria* and *Archaea* and the relative abundance information derived from 16S rRNA gene amplicon sequencing. Dashed lines indicate that some sampling time points could not be analyzed by quantitative PCR. Arrows indicate samples used for metagenome and metatranscriptome sequencing at days 172 and 185. Source data is provided as Supplementary Data

Peat soil from an acidic fen (pH 4–5) served as an inoculum for a bioreactor that was operated as a continuous culture at pH 4.5. The conditions were periodically switched between oxic for 1 week and anoxic/sulfate-reducing for 4 weeks over a period of 211 days (Fig. 1A). Air saturation was set to 50% (corresponding to 133 µM O_2_) during the oxic and to 0% during the anoxic periods. Oxic and anoxic conditions were maintained stable by the control loop of the bioreactor through the supply with compressed air or 100% N_2_, respectively. The medium contained the abundant terrestrial plant polysaccharide pectin (0.5 g l^−1^) and to a lesser extent glucose (0.1 g l^−1^) as carbon source and electron donor and sulfate (0.1 g l^−1^) as an electron acceptor, thus providing relevant substrates for different functional guilds of primary and secondary organic matter degraders. After 40 days of bioreactor operation, there was always sulfate reduction activity in the anoxic phases, with sulfate reduction rates ranging between 18 µmol SO_4_ l^−1^ day^−1^ and 104 µmol SO_4_ l^−1^ day^−1^ (Fig. 1A). During oxic phases, sulfate concentrations increased again, likely due to microbial sulfur oxidation [55]. As revealed by 16S rRNA gene amplicon sequencing, the microbial community was dominated by representatives of the phyla *Pseudomonadota* and *Acidobacteriota* throughout bioreactor operation, which together accounted for 53–93% of all prokaryotes [55]. A facultatively anaerobic sulfate-reducing bacterium affiliating with the *Acidobacteriota* was identified as well and has been described in detail elsewhere [55]. Here, we focused on the strictly anaerobic peatland SRB [34, 46–49]. *Desulfobacca* was the most abundant SRB in the inoculum (Supplementary Table S1). Amplicon sequence variants (ASVs) summed within the genus *Desulfobacca* accounted for 2.3% of all prokaryotic 16S rRNA gene amplicons. SRBs like *Desulfomonile* (0.2%), *Syntrophobacter* (0.25%), and *Desulfosporosinus* (0.33%) were identified in the inoculum as well (Supplementary Table S1). The most abundant *Desulfosporosinus* ASV at the start of bioreactor operation (ASV81, 0.15%) shared 100% nucleotide identity to the 16S rRNA gene of *Candidatus* (*Ca*.) Desulfosporosinus infrequens is a low-abundance SRB that is cosmopolitan in freshwater wetlands [46, 53, 54]. Different ASVs of SRB became successively abundant in the bioreactor over the period of more than 200 days of operation (Fig. 1B, C). The most abundant ASVs that reached relative abundances > 1% were assigned to *Desulfovibrio* (ASV30, 2.9% at day 98), *Desulfocapsaceae* (ASV36, 2.4% at day 211), and *Desulfosporosinus* (ASV32, 1.9% at day 134 and ASV41, 1.9% at day 204). While those ASVs were of low abundance at the start of bioreactor operation (< 0.02% relative abundance), other ASVs assigned to *Desulfomonile* and *Syntrophobacter* maintained a low-abundance state throughout the operation period or only reached a low relative abundance (< 0.2%) (Supplementary Table S1). Due to the high numbers of 16S rRNA gene copies found in the genomes of *Desulfosporosinus* spp. (up to 11 copies), the amplicon survey likely overestimates their relative abundance [56]. Responsive SRB-ASVs were typically characterized by a decrease in relative abundance during oxic conditions and an increase during anoxic conditions (Fig. 1B). This suggests that the periodically fluctuating redox conditions selected for sulfate reducers endure prolonged oxygen stress and efficiently recover after regularly occurring oxic periods. Total bacterial and archaeal 16S rRNA genes were determined in the bioreactor using quantitative PCR (qPCR) [55]. By combining the relative abundance of the ASVs and the total 16S rRNA gene copies as revealed by qPCR, we verified that *Desulfovibrio* ASV30, *Desulfocapsaceae* ASV36, *Desulfosporosinus* ASV32, and ASV41 were also enriched in absolute numbers and as such were actively growing during the cultivation period (Fig. 1C).

**Fig. 2:**
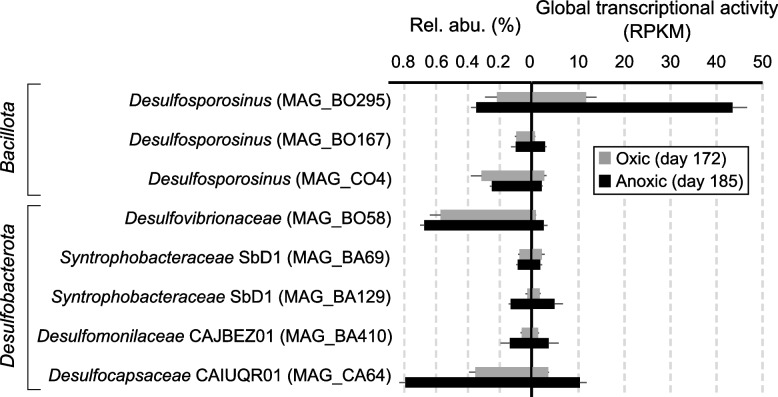
Relative genome abundance and global transcriptional activity of three *Bacillota* MAGs and five *Desulfobacterota* MAGs. The relative abundance was determined using metagenomic OTUs (mOTUs) based on the universal single copy marker gene COG0172 (seryl-tRNA synthetase). Genome-wide transcriptional activity is depicted as reads per kilobase million (RPKM). Error bars represent the standard deviation of three (relative abundance) or four (transcriptional activity) technical replicates. Source data is provided as Supplementary Data

In a genome-centric metagenomics approach at day 172 (oxic period) and day 185 (anoxic period), 106 high- or medium-quality metagenome-assembled genomes (MAGs) were recovered from the bioreactor, which was published elsewhere [55]. Three of those MAGs (BO167, BO295, and CO4) affiliated with *Desulfosporosinus* with a genome completeness of > 91% and a potential contamination level of < 4%. The three MAGs shared *Ca*. D. infrequens as their closest relative (Supplementary Figure S2). Average nucleotide identities (ANI) between these high-quality, nearly complete genomes ranged between 82.2% and 86.6%, which is below the intra-species cut-off of 95% [57] and suggests that these MAGs represent individual species. Besides *Desulfosporosinus* representatives, MAGs of typical peatland sulfate reducers from the phylum *Desulfobacterota* were recovered from the bioreactor metagenome as well. Those affiliated with the families *Syntrophobacteraceae* (MAGs BA129 and BA69; 79.6% ANI), *Desulfomonilaceae* (MAG BA410), *Desulfocapsaceae* (MAG CA64), and *Desulfovibrionaceae* (MAG BO58) (Supplementary Figure S2). Their genome completeness was > 70% and the potential contamination level was below 4% (Supplementary Table S2). None of the eight sulfate reducer MAGs from the bioreactor contained a 16S rRNA gene, and therefore the MAGs cannot be directly linked to the ASVs. Based on the single copy marker gene COG0172 (seryl-tRNA synthetase), their relative abundance in the metagenomes was estimated (Fig. 2). In accordance with the 16S rRNA gene approach, MAGs affiliating with *Desulfocapsaceae* (MAG CA64), *Desulfovibrionaceae* (MAG BO58) and *Desulfosporosinus* (BO167, BO295 and CO4) were the most abundant SRB in the metagenome (Fig. 2). *Syntrophobacteraceae* (MAGs BA129 and BA69) and *Desulfomonilaceae* (MAG BA410) on the other hand only reached a relative abundance up to 0.1%.

### Global transcript levels drop during oxic conditions in peatland SRB

**Fig. 3:**
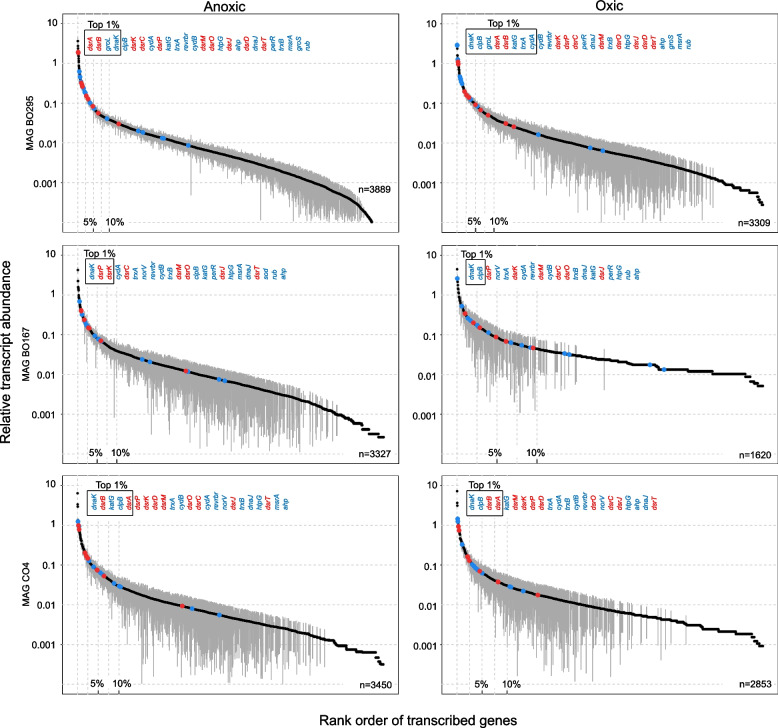
Ranked relative transcript abundance of genes in three *Bacillota* SRB-MAGs under anoxic (left column) and oxic conditions (right column). The mean ± standard deviation of four replicates is depicted. If the mean minus standard deviation gave a negative value, the standard deviation could not be shown due to the logarithmic scale. The total number of transcribed genes is shown for each MAG in the lower right corner. Sulfate reduction pathway genes found and transcribed in the genome are highlighted in red. Genes coding for oxygen defense proteins are highlighted in blue. The top 1% of transcribed genes are indicated by the black box. The order of the sulfate reduction pathway and oxygen defense encoding genes shown above corresponds to their rank order. Source data is provided as Supplementary Data

**Fig. 4:**
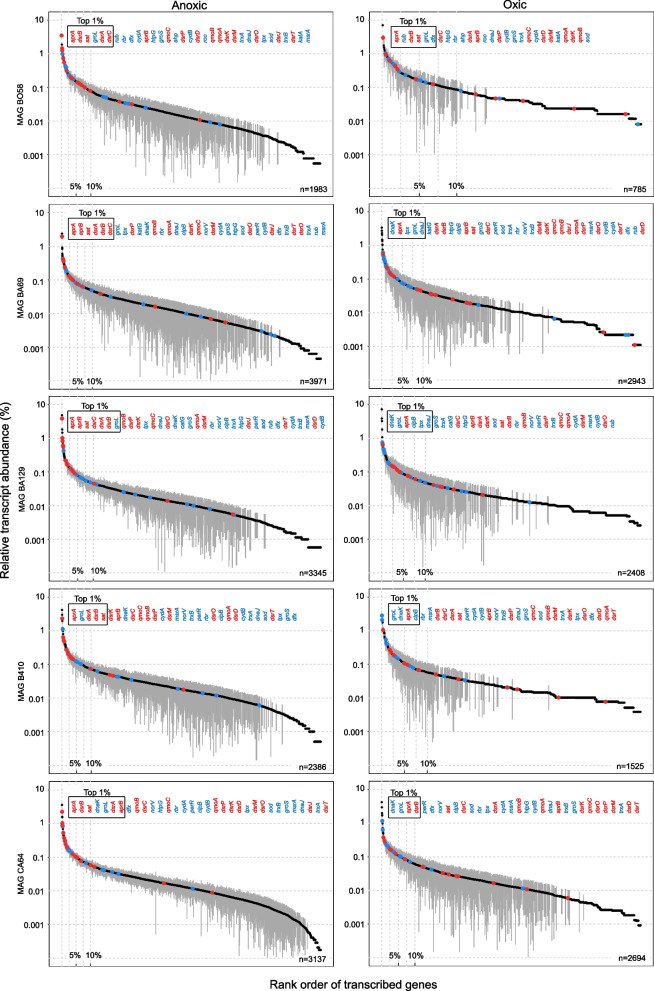
Ranked relative transcript abundance of genes in five *Desulfobacterota* MAGs under anoxic (left column) and oxic conditions (right column). The mean ± standard deviation of four replicates is depicted. If the mean minus standard deviation gave a negative value, the standard deviation could not be shown due to the logarithmic scale. The total number of transcribed genes is shown for each MAG in the lower right corner. Sulfate reduction pathway genes found and transcribed in the genome are highlighted in red. Genes coding for oxygen defense proteins are highlighted in blue. The top 1% of transcribed genes are indicated by the black box. The order of the sulfate reduction pathway and oxygen defense encoding genes shown above corresponds to their rank order. Source data is provided as Supplementary Data

Metatranscriptomes sequenced from day 172 (oxic period) and day 185 (anoxic period) revealed that all SRB analyzed by genome-resolved metatranscriptomics were transcriptionally active and survived the periodical exposure to 50% air-O_2_ saturation (Fig. 2, Supplementary Tables S3–S10). However, genome-wide transcriptional activity was substantially lower under oxygen exposure for most SRB, except *Syntrophobacteraceae* MAG BA69 and *Desulfosporosinus* MAG CO4 which maintained similar transcription levels under both conditions (Fig. 2). *Desulfosporosinus* MAG BO295 was clearly predominating in the transcriptome from the anoxic phase and accounted for 72% of all SRB-MAG transcripts (Fig. 2). Analysis of the ranked relative transcript abundance in the individual SRB-MAGs indicated a global transcriptomic response to oxygen stress. Transcripts of most sulfate reduction pathway genes ranked within the top 10% under anoxic conditions, with *dsrAB* transcripts being consistently in the top 1% (Figs. 3 and 4). Sulfate reduction pathway genes were also transcribed under oxic conditions; however, their transcript levels shifted towards a lower rank accompanied by a substantially lower number of total transcribed genes under oxygen stress, which was observed for all MAGs (Figs. 3 and 4).

**Fig. 5:**
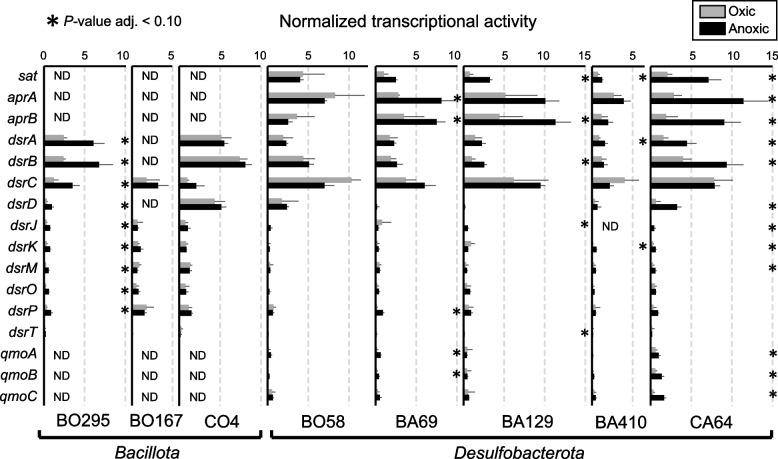
Normalized transcriptional activity of sulfate reduction pathway genes during oxic and anoxic conditions across three *Bacillota* MAGs and five *Desulfobacterota* MAGs. RPKM values of gene transcripts were divided by the average RPKM transcript values of 16 ribosomal protein-encoding genes that were identified in all MAGs (Supplementary Table S11). This was done to account for the strong global transcript decreases under oxic conditions (see also Fig. 2). Grey and black asterisks indicate significantly higher normalized transcript levels (*P* value < 0.10) under oxic and anoxic conditions, respectively. ND, gene not found in the genome. Assigned taxonomy: MAG BO295, BO167 and CO4, *Desulfosporosinus*; MAG BO58, *Desulfovibrionaceae*; MAG BA69 and BA129, *Syntrophobacteraceae*; MAG BA410, *Desulfomonilaceae*; MAG CA64, *Desulfocapsaceae*. Source data is provided as Supplementary Data

A generally lower transcriptional activity among strictly anaerobic SRB under oxygen exposure can be expected [20], but makes a direct comparison of transcriptional activities (RPKM) across oxic and anoxic conditions precarious [58]. Therefore, RPKM counts were further normalized against the averaged RPKM counts of 16 ribosomal protein-encoding genes that were identified in all MAGs (Supplementary Table S11). The latter served as a general transcriptional activity marker and was used to account for the strong global transcript decreases under oxic conditions. Normalized transcript levels for sulfate reduction pathway genes were in most SRBs either elevated or equally high under anoxic as compared to oxic conditions (Fig. 5). Similarly, high transcript levels or abundance of proteins for sulfate reduction have been shown for SRB during syntrophic/fermentative growth in the absence of sulfate [59, 60]. The redox-sensing transcriptional regulator Rex was linked to the regulation of the transcription of *sat* in *Desulfovibrio vulgaris* [61]. Rex is also encoded in five of the enriched peatland SRB in the bioreactor (BO58, BA69, BA129, BO167 and BO295). However, its transcription levels were low and similar under both conditions (Supplementary Tables S3–S10). Taken together, this might indicate that the sulfate reduction pathway is under a global transcriptional control rather than being specifically down-regulated under oxic conditions in the enriched peatland SRB.

### Different strategies to cope with oxygen stress exist among peatland SRB

It was intriguing that diverse SRB survived regular exposure to relatively high oxygen concentrations (133 µM O_2_) and was even enriched in the bioreactor (Fig. 1, Supplementary Table S1). This stands in contrast to reports on well-studied model SRB, whose viability usually decreases upon exposure to such high oxygen levels [15, 20, 62–64]. The oxygen concentration during the 1-week oxic periods in the bioreactor was approximately one order of magnitude higher than oxygen levels that could be tolerated for a limited time period by pure cultures of different SRB [64–67]. Co-cultivation with facultative anaerobic bacteria was shown before to increase the oxygen tolerance of SRB while maintaining sulfate reduction activity at 87 µM O_2_ [68].

**Fig. 6:**
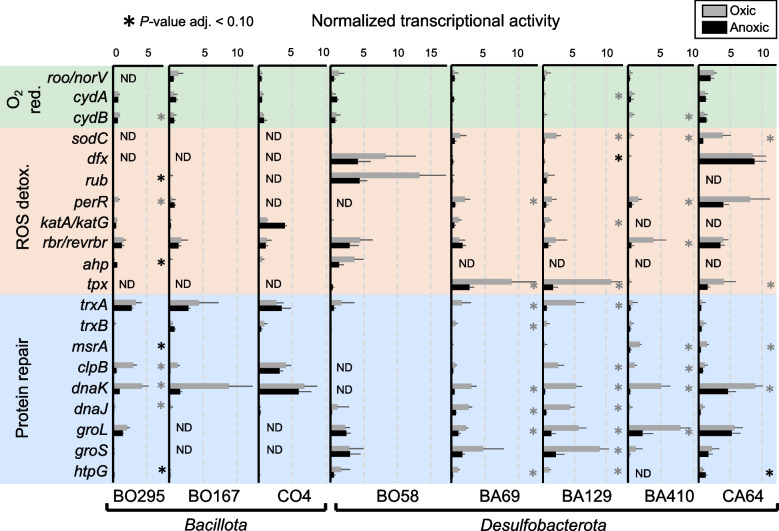
Normalized transcriptional activity of oxygen defense-encoding genes during oxic and anoxic conditions across three *Bacillota* MAGs and five *Desulfobacterota* MAGs. Shown are selected genes encoding proteins involved in oxygen reduction, reactive oxygen species (ROS) detoxification, and protein repair. If multiple gene copies were found in a MAG, the gene with the highest transcript levels is depicted. A more detailed list can be found in Supplementary Table S12. RPKM values of gene transcripts were divided by the average RPKM transcript values of 16 ribosomal protein-encoding genes that were identified in all MAGs (Supplementary Table S11). This was done to account for the strong global transcript decreases under oxic conditions (see also Fig. 2) and to identify genes that were upregulated under oxygen stress. Grey and black asterisks indicate significantly higher normalized transcript levels (*P* value < 0.10) under oxic and anoxic conditions, respectively. ND, gene not found in the genome. Assigned taxonomy: MAG BO295, BO167 and CO4, *Desulfosporosinus*; MAG BO58, *Desulfovibrionaceae*; MAG BA69 and BA129, *Syntrophobacteraceae*; MAG BA410, *Desulfomonilaceae*; MAG CA64, *Desulfocapsaceae*. Source data is provided as Supplementary Data

In the following, we focused on the enzymatic mechanisms that were potentially involved in oxygen stress defense. Therefore, we targeted a selection of genes encoding proteins for oxygen reduction, reactive oxygen species (ROS) detoxification, and repair of oxidized proteins to examine how peatland SRB deals with periodic oxygen exposure. Annotations of the selected genes were additionally validated by phylogenetic analysis (Supplementary Figure S2). Interestingly, oxic conditions did not trigger global differential expression of genes encoding oxygen defense proteins. Rather, each MAG showed individual response patterns with some of the activated genes being equally well expressed under both, oxic and anoxic conditions. We categorized and highlighted the most pronounced responses in the following (Fig. 6 and Supplementary Table S12).

Molecular oxygen can directly inactivate enzymes of the sulfate reduction metabolism [4]. The capability of oxygen reduction is prevalent among SRB by means of the cytoplasmic bifunctional rubredoxin:oxygen oxidoreductase/nitric oxide reductase (Roo/NorV) [7, 8, 69] and/or membrane-bound oxygen reductases such as the *bd*-type oxidase (CydAB) [70]. Homologs of Roo/NorV were encoded in all MAGs except in *Desulfosporosinus* MAG BO295 and also transcribed (Fig. 6 and Supplementary Figure S2). All MAGs further encoded cytochrome *bd*-type oxidases, which are known to play an important role in protection against oxidative stress [10]. However, genes coding for Roo/NorV and Cyd did not show pronounced differences in normalized transcript levels among all SRB (Fig. 6). The five *Desulfobacterota* MAGs encoded in addition to other terminal oxidases that could be involved in oxygen reduction, i.e., cytochrome *c* oxidase (*caa*_3_-type) and/or cytochrome *bo*_3_ ubiquinol oxidase (Supplementary Table S12). The low transcript levels of the respective genes compared to the transcription of *cyd* genes encoding the *bd*-type oxidases (Supplementary Table S12) agree with earlier observations in the model organism *Desulfovibrio vulgaris* [71]. In *D. vulgaris,* the transcription of *bd*-type oxidase was upregulated under low oxygen concentrations (0.1%) [20], while the cytochrome *c* oxidase is constitutively expressed and not upregulated with oxygen [72].

The major toxic effect of oxygen results in the endogenous formation of ROS like superoxide, peroxides, and hydroxyl radicals. All can lead to damage to macromolecules like protein and DNA [6]. Genes encoding either superoxide dismutase (*sodC*) or superoxide reductase/desulfoferredoxin (*dfx*) for superoxide elimination showed high normalized transcript levels (mainly under oxic conditions) in all *Desulfobacterota* MAGs but were not identified or had very low transcript levels in the *Desulfosporosinus* MAGs (Fig. 6). In *Desulfovibrionaceae* MAG BO58, *dfx* was preferentially transcribed in combination with *rub*, which encodes the necessary electron transfer protein rubredoxin. In contrast, *Syntrophobacteraceae* MAGs BA69 and BA129, as well as Desulfomonilaceae MAG BA410, preferentially transcribed *sodC. Desulfocapsacaea* MAG CA64 transcribed both, *sodC* and *dfx*. Unlike SodC, oxygen is not a product of Dfx, which might be a more effective antioxidant system for anaerobes [17].

Peroxide detoxification can be facilitated by various enzyme systems involving catalase (KatA), catalase-peroxidase (KatG), rubrerythrin/reverse rubrerythrin (Rbr/revRbr), alkyl hydroperoxide reductase (Ahp) and thiol peroxidase (Tpx). In most SRB-MAGs, genes encoding rubrerythrins were transcribed equally well under oxic and anoxic conditions. The only exception was *Desulfomonilaceae* MAG BA410, where a significant (*P* value < 0.10) upregulation was detected under oxic conditions (Fig. 6). *Syntrophobacteraceae* MAGs BA69 and BA129 encoded a catalase-peroxidase (*katG*) for hydrogen peroxide degradation in direct proximity to the peroxide response regulon (*perR*), which both showed elevated or even significantly higher (*P* value < 0.10) normalized transcript levels under oxic conditions (Fig. 6 and Supplementary Table S12). This stands in contrast to *D. vulgaris*, which upregulated genes in the peroxide response regulon only under weak oxidative stress when exposed to 0.1% oxygen and downregulated the genes when exposed to higher oxygen concentrations [20]. A significant transcriptional increase under oxic conditions was also observed for the thiol peroxidase-encoding gene (*tpx*) in *Syntrophobacteraceae* MAGs BA69 and BA129 as well as in *Desulfocapsacaea* MAG CA64. Similar to *D. vulgaris*, the thiol peroxidases might have a key function in the defense mechanism against oxidative stress in members of the *Syntrophobacteraceae* and *Desulfocapsacaea* [73]*.* A pronounced difference among the five *Desulfobacterota* MAGs was the much higher normalized transcription levels of ROS detoxification genes for the *Desulfovibrionaceae* and *Desulfocapsacaea* MAGs under both conditions (Fig. 6). Interestingly, all *Desulfosporosinus* MAGs showed transcription of ROS detoxification genes as well but most genes, except *perR* of MAG BO295, showed no pronounced increase at the normalized transcript level under oxic conditions (Fig. 6).

Genes encoding protein repair showed elevated normalized transcript levels under oxic conditions across the different MAGs as well (Fig. 6). Most MAGs significantly overexpressed genes encoding the ClpB-DnaK chaperone system. Quite similarly, genes encoding the chaperons GroL and GroS had elevated or even significantly higher normalized transcript levels in the *Syntrophobacteraceae* MAGs BA69 and BA129 and *Desulfomonilacaea* MAG BA410. In all *Desulfobacterota* MAGs, a thioredoxin encoding gene (*trxA*) had elevated or even significantly higher normalized transcript levels under oxic conditions. In contrast, *trxA* normalized transcript levels were high in all *Desulfosporosinus* MAGs, but under both oxic and anoxic conditions (Fig. 6). Thioredoxins can fulfill various cellular functions including the reduction of cytoplasmic proteins and the direct or indirect elimination of ROS [74].

The ability to form spores differentiates SRB of the *Bacillota* such as *Desulfosporosinus* from non-spore-forming SRB of the *Desulfobacterota*. Transcriptional activity of genes involved in sporulation was consistently low among the *Desulfosporosinus* MAGs BO167, BO295, and CO4 under oxic and anoxic conditions suggesting that a switch to a dormant state was not the major strategy to cope with periodically occurring oxygen stress (Supplementary Tables S3–S5).

## Conclusions

Alternating oxic and anoxic conditions applied in the sulfur-cycling bioreactor allowed functional insights into the lifestyle of in situ-relevant sulfate reducers known to drive the cryptic sulfur cycle in low-sulfate environments like peatlands. Typical peatland SRB affiliating with the genus *Desulfosporosinus* and the families *Syntrophobacteraceae*, *Desulfomonilaceae*, *Desulfocapsaceae*, and *Desulfovibrionaceae* survived oxygen concentrations above 130 µM when periodically exposed for 1 week. They even established large and stable populations in the percent range of the overall microbial community (Fig. 1). Perpetuation of gene expression for energy metabolism was observed for all SRB in the bioreactor (Figs. 3, 4, and 5). A retained expression of core metabolism genes and a decrease in regulation was also observed for microbial communities from tidal sediments under strong environmental fluctuations, which was attributed to a specific adaption to periodic oxygen exposure [75, 76]. The decrease in genome-wide transcriptional activity under oxic conditions among many SRBs from the bioreactor (Fig. 2) was typically accompanied by a decrease in their relative abundance (Fig. 1 and Supplementary Table S1). This implies that these SRBs undergo cycles of growth and death during favorable and unfavorable conditions, respectively. Transcription of genes encoding well-known oxygen resistance proteins was a strategy shared by all SRB in the bioreactor to survive and endure oxic conditions (Fig. 6). However, the transcriptional response pattern for oxygen resistance varied substantially among SRB. Other strategies such as aggregation with oxygen-scavenging microorganisms [77] could have been used concurrently by some SRBs in the bioreactor community. It is noteworthy that three SRBs (*Desulfosporosinus* MAG CO4, *Desulfovibrionaceaea* MAG BO58, and *Desulfocapsaceaea* MAG CA64) maintained high transcript levels of oxygen defense genes even under anoxic conditions (Fig. 6). When compared to the ASV data, also here several *Desulfosporosinus* representatives, one *Desulfovibrionaceae* representative, and one *Desulfocapsacaea* representative established populations of 0.3–2.4% relative abundance after more than half a year of bioreactor operation (Fig. 1). As such, a constant expression of oxygen defense genes may be keys adaptation to withstand periodically occurring changes in redox regimes in these otherwise strictly anaerobic SRBs.

## Methods

### Sample collection and bioreactor operation

The soil was sampled from the long-term experimental field “Schlöppnerbrunnen II”, an acidic fen located in south-eastern Germany (50°08′38′′N, 11°51′41′′E). A detailed description of the study site was addressed in earlier publications [34, 41]. Peat soil was sampled from the surface layer (10–20 cm depth) and stored at 4 °C for 3 days until inoculation of the bioreactor. Details on bioreactor operation were published elsewhere [55]. Briefly, the medium contained glucose (0.1 g l^−1^) and pectin (0.5 g l^−1^) as electron donor and carbon source and sulfate (0.1 g l^−1^) as electron acceptor. The bioreactor was operated as a continuous culture with a dilution rate of 0.025 day^−1^ under anoxic conditions and as a fed-batch under oxic conditions. It was consecutively exposed to oxic (50% air-O_2_ saturation, 1-week duration) and anoxic (100% N_2_, 4-week duration) cycles over a period of 211 days. Dissolved oxygen in the bioreactor was monitored with an optical LumiSense DO sensor (Getinge) at 2-s interval readouts to the bioreactor control unit. This was manually double-checked on a regular basis (almost every working day). Set oxic or anoxic conditions were maintained stable by the control loop of the bioreactor through continuous supply with compressed air or 100% N_2_, respectively. Percent air-O_2_ saturation was converted to dissolved oxygen concentrations according to Weiss [78].

### 16S rRNA gene amplicon sequencing and quantitative PCR

The procedures of DNA extraction and subsequent sequencing as well as data processing were described in detail elsewhere [55]. In brief, the bacterial diversity in all bioreactor samples was determined by analyzing the hypervariable V4 region of the 16S rRNA gene using MiSeq (Illumina, CA, USA) sequencing of barcoded amplicons. Barcoded amplicons were prepared using primers 515F/806R [79–81] targeting *Bacteria* and *Archaea*. Denoising and chimera filtering was performed using the DADA2 pipeline v2022.2.0 [82] implemented in Qiime2 v2022.2.1 [83]. Representative amplicon sequence variants (ASVs) were classified using the SINA classifier and the SILVA SSU reference database release 138.1 [84, 85]. ASVs that affiliated with known and putative sulfate-reducing bacteria were selected for further downstream analysis (Fig. 1 and Supplementary Table S1). Total 16S rRNA genes of *Bacteria* and *Archaea* were quantified as described earlier [55].

### Combined genome-centric metagenomics and metatranscriptomics

Samples were taken from an oxic phase at day 172 and from an anoxic phase at day 185 from the bioreactor for metagenome (three replicates per condition) and metatranscriptome (four replicates per condition) sequencing as described before [55]. In brief, metagenome libraries were prepared using the NEBNext Ultra II DNA library prep kit (New England Biolabs, MA, USA) and sequenced on an Illumina NextSeq 4000 platform (2 × 150 bp). Metagenome reads were quality trimmed at quality score Q20 using bbduk.sh v38.22 [86] and assembled using MEGAHIT v1.2.9 [87]. Assembled contigs were binned using MetaBAT2 v2.12.1 [88], MaxBin2 v 2.2.7 [89] and MetaCoAG v1.0 [90] and dereplicated with DAS_Tool v1.1.4 [91]. The quality of the metagenome-assembled genomes (MAGs) was assessed using CheckM v1.0.7 [92]. Taxonomy was assigned to the MAGs using GTDB-Tk v2.1.1 [93]. All MAGs recovered from the bioreactor were initially annotated using MetaErg v1.2.0 [94]. Annotations were validated and extended by comparison to annotations obtained through eggNOG v2.1.12 [95] and METABOLIC v4.0 [96]. Genes encoding the sulfate reduction pathway were further identified using DiSCo v1.0.0 [97], a tool specifically dedicated to identifying genes coding for enzymes involved in dissimilatory sulfur metabolism. Genes coding for proteins involved in oxygen defense were further identified by BLAST v2.9.0 [98] using the Swiss-Prot [99] database as a reference. To confirm the accuracy of annotations for genes encoding oxygen defense proteins, phylogenetic analyses were performed. Here, sequences of related proteins were collected from Swiss-Prot and RefSeq [100]. Sequence alignments were performed with MAFFT v7.453 [101] (E-INS-i) and used for phylogenetic reconstruction with IQ-TREE v2.2.0.3 [102] using ultrafast bootstrap analysis (*n* = 1000) after automatic substitution model selection. The relative abundance of the MAGs was estimated by using the metagenomic OTU (mOTU) approach [103] with the universal single copy marker gene COG0172. A concatenated amino acid alignment deduced from 120 single copy marker genes [104] encoded on 59 reference genomes and the eight SRB-MAGs from the bioreactor was used for phylogenomic reconstruction with IQ-TREE v2.2.0.3 [102] using ultrafast bootstrap analysis (*n* = 1000) after automatic substitution model selection (LG + F + R6).

For metatranscriptome sequencing, libraries were prepared from ribosomal RNA-depleted (Vazyme Biotech, Nanjing, China) total RNA using Illumina’s TruSeq stranded mRNA kit. Sequencing was performed on an Illumina NextSeq 4000 platform (2 × 150 bp). Raw metatranscriptome reads were trimmed at quality score Q20 using bbduk.sh v38.22 and remaining rRNA reads were removed with RiboDetector v0.2.6 [105]. To estimate the global transcriptional activity of MAGs, RPKM (reads per kilobase million) tables were generated using bbmap.sh v38.22 [86]. The strong global transcript decreases under oxic conditions as observed for most of the sulfate-reducing bacteria in the bioreactor made the use of count-based methods for comparisons across different conditions inappropriate, as such approaches assume that most genes were not differentially expressed [106, 107]. Therefore, RPKM counts were further normalized against the average transcript counts of 16 ribosomal protein-encoding genes that were identified in all MAGs (Supplementary Table S11). Statistical analysis of changes in gene expression was performed using the Wilcoxon rank-sum test. FDR-corrected *P* values < 0.10 were considered statistically significant given the limited sensitivity of nonparametric tests such as the Wilcoxon rank-sum test and the small number of replicates (here *n* = 4) possible in metatranscriptomics studies.

## Supplementary Information

Supplementary Material 1: Supplementary Figure S1. Maximum likelihood phylogenomic reconstruction of all metagenome assembled genomes (MAGs) that affiliated with sulfate-reducing bacteria recovered from the bioreactor (bold). The tree was constructed based on concatenated amino acid alignment deduced from 120 single copy marker genes [104]. Bootstrap support ≥90% is indicated by black dots. The asterisk denotes a facultatively anaerobic, sulfate-reducing *Acidobacteriota* representative that has been published elsewhere [55]. Abbreviations of the families are as follows: Bry, *Bryobacteraceae*; D’vib, *Desulfovibrionaceae*; D’mon, *Desulfomonilaceae*; D’cap, *Desulfocapsaceae*; Syn, *Syntrophobacteraceae*.

Supplementary Material 2: Supplementary Figure S2. Maximum likelihood phylogeny of selected oxygen defense proteins identified in the eight SRB-MAGs. MAG-derived sequences are highlighted in blue. Manually annotated and reviewed reference protein sequences collected from the Swiss-Prot database are highlighted in red. All trees were midpoint rooted and bootstrap support ≥ 90% is indicated by black dots.

Supplementary Material 3: Supplementary Table S1. Relative abundance of amplicon sequence variants (ASVs) that affiliated with known and putative sulfate-reducing bacteria. Supplementary Table S2. Basic genome characteristics of the metagenome assembled genomes (MAGs) that were assigned to sulfate-reducing bacteria. Supplementary Table S3. Annotations and global transcriptional activity (reads per kilobase million, RPKM) of *Desulfosporosinus* MAG BO167. Supplementary Table S4. Annotations and global transcriptional activity (reads per kilobase million, RPKM) of *Desulfosporosinus* MAG BO295. Supplementary Table S5. Annotations and global transcriptional activity (reads per kilobase million, RPKM) of *Desulfosporosinus* MAG CO4. Supplementary Table S6. Annotations and global transcriptional activity (reads per kilobase million, RPKM) of *Humidesulfovibrio* MAG BO58. Supplementary Table S7. Annotations and global transcriptional activity (reads per kilobase million, RPKM) of *Syntrophobacteraceae* SbD1 MAG BA69. Supplementary Table S8. Annotations and global transcriptional activity (reads per kilobase million, RPKM) of *Syntrophobacteraceae* SbD1 MAG BA129. Supplementary Table S9. Annotations and global transcriptional activity (reads per kilobase million, RPKM) of *Desulfomonilaceae* CAJBEZ01 MAG BA410. Supplementary Table S10. Annotations and global transcriptional activity (reads per kilobase million, RPKM) of *Desulfocapsaceae* CAIUQR01 MAG CA64. Supplementary Table S11. Transcription levels (RPKM) of ribosomal protein-encoding genes used to normalize transcriptional activity as presented in Figure 5 and Figure 6. Supplementary Table S12. Annotations and global transcriptional activity (reads per kilobase million, RPKM) of genes that encode proteins involved in oxygen reduction, reactive oxygen species (ROS) detoxification and protein repair. Genes were collected from five SRB-MAGs affiliating with the *Desulfobacterota* and three SRB-MAGs affiliating with the *Bacillota*.

Supplementary Material 4.

## Data Availability

All nucleotide sequences obtained in this study have been deposited in GenBank. Amplicon sequences from the 16S rRNA gene survey were deposited in NCBI BioProject PRJNA923133. Metagenomes, metatranscriptomes, and MAGs are available under BioProject PRJNA923161.

## References

[1] Diao M, Dyksma S, Koeksoy E, Ngugi DK, Anantharaman K, Loy A, et al. Global diversity and inferred ecophysiology of microorganisms with the potential for dissimilatory sulfate/sulfite reduction. FEMS Microbiol Rev. 2023;47:fuad058. 37796897 10.1093/femsre/fuad058 PMC10591310

[2] Ferreira D, Barbosa ACC, Oliveira GP, Catarino T, Venceslau SS, Pereira IAC. The DsrD functional marker protein is an allosteric activator of the DsrAB dissimilatory sulfite reductase. Proc Natl Acad Sci. 2022;119:e2118880119. 35064091 10.1073/pnas.2118880119 PMC8794893

[3] Rabus R, Venceslau SS, Wöhlbrand L, Voordouw G, Wall JD, Pereira IAC. Chapter two - a post-genomic view of the ecophysiology, catabolism and biotechnological relevance of sulphate-reducing prokaryotes. In: Poole RK, editor. Advances in microbial physiology. Academic Press; 2015. p. 55–321. 10.1016/bs.ampbs.2015.05.002 26210106

[4] Brioukhanov A, Pieulle L, Dolla A. Antioxidative defense systems of anaerobic sulfate-reducing microorganisms. Curr Res Technol Educ Appl Microbiol Microb Biotechnol . 2010;1:148–59.

[5] Rabus R, Hansen TA, Widdel F. Dissimilatory sulfate- and sulfur-Reducing prokaryotes. In: Rosenberg E, DeLong EF, Lory S, Stackebrandt E, Thompson F. (eds) The Prokaryotes. Berlin: Springer; 2013. 10.1007/978-3-642-30141-4_70.

[6] Lu Z, Imlay JA. When anaerobes encounter oxygen: mechanisms of oxygen toxicity, tolerance and defence. Nat Rev Microbiol. 2021;19:774–85. 34183820 10.1038/s41579-021-00583-y PMC9191689

[7] Chen L, Liu MY, Legall J, Fareleira P, Santos H, Xavier AV. Rubredoxin oxidase, a new flavo-hemo-protein, is the site of oxygen reduction to water by the “strict anaerobe” *Desulfovibrio gigas*. Biochem Biophys Res Commun. 1993;193:100–5. 8503894 10.1006/bbrc.1993.1595

[8] Romão CV, Vicente JB, Borges PT, Frazão C, Teixeira M. The dual function of flavodiiron proteins: oxygen and/or nitric oxide reductases. J Biol Inorg Chem. 2016;21:39–52. 26767750 10.1007/s00775-015-1329-4

[9] Pereira IAC, Ramos A, Grein F, Marques M, Da Silva S, Venceslau S. A comparative genomic analysis of energy metabolism in sulfate reducing bacteria and archaea. Front Microbiol. 2011;2:69. 10.3389/fmicb.2011.00069. 21747791 10.3389/fmicb.2011.00069 PMC3119410

[10] Ramel F, Amrani A, Pieulle L, Lamrabet O, Voordouw G, Seddiki N, et al. Membrane-bound oxygen reductases of the anaerobic sulfate-reducing *Desulfovibrio vulgaris* Hildenborough: roles in oxygen defence and electron link with periplasmic hydrogen oxidation. Microbiology. 2013;159:2663–73. 24085836 10.1099/mic.0.071282-0

[11] Dolla A, Fournier M, Dermoun Z. Oxygen defense in sulfate-reducing bacteria. J Biotechnol. 2006;126:87–100. 16713001 10.1016/j.jbiotec.2006.03.041

[12] Voordouw JK, Voordouw G. Deletion of the *rbo* gene increases the oxygen sensitivity of the sulfate-reducing bacterium *Desulfovibrio vulgaris* Hildenborough. Appl Environ Microbiol. 1998;64:2882–7. 9687445 10.1128/aem.64.8.2882-2887.1998 PMC106787

[13] Dos Santos WG, Pacheco I, Liu M-Y, Teixeira M, Xavier AV, LeGall J. Purification and characterization of an iron superoxide dismutase and a catalase from the sulfate-reducing bacterium *Desulfovibrio gigas*. J Bacteriol. 2000;182:796–804. 10633116 10.1128/jb.182.3.796-804.2000 PMC94345

[14] Lumppio HL, Shenvi NV, Summers AO, Voordouw G, Kurtz DM. Rubrerythrin and rubredoxin oxidoreductase in *Desulfovibrio vulgaris*: a novel oxidative stress protection system. J Bacteriol. 2001;183:101–8. 11114906 10.1128/JB.183.1.101-108.2001 PMC94855

[15] Fournier M, Zhang Y, Wildschut JD, Dolla A, Voordouw JK, Schriemer DC, et al. Function of oxygen resistance proteins in the anaerobic, sulfate-reducing bacterium *Desulfovibrio vulgaris* Hildenborough. J Bacteriol. 2003;185:71–9. 12486042 10.1128/JB.185.1.71-79.2003 PMC141827

[16] Pinto AF, Rodrigues JV, Teixeira M. Reductive elimination of superoxide: Structure and mechanism of superoxide reductases. Biochimica et Biophysica Acta (BBA) - Proteins and Proteomics. 2010;1804:285–97. 19857607 10.1016/j.bbapap.2009.10.011

[17] Sheng Y, Abreu IA, Cabelli DE, Maroney MJ, Miller A-F, Teixeira M, et al. Superoxide dismutases and superoxide reductases. Chem Rev. 2014;114:3854–918. 24684599 10.1021/cr4005296 PMC4317059

[18] Zhang W, Culley DE, Hogan M, Vitiritti L, Brockman FJ. Oxidative stress and heat-shock responses in *Desulfovibrio vulgaris* by genome-wide transcriptomic analysis. Antonie Van Leeuwenhoek. 2006;90:41–55. 16680520 10.1007/s10482-006-9059-9

[19] Pereira PM, He Q, Valente FMA, Xavier AV, Zhou J, Pereira IAC, et al. Energy metabolism in *Desulfovibrio vulgaris* Hildenborough: insights from transcriptome analysis. Antonie Van Leeuwenhoek. 2008;93:347–62. 18060515 10.1007/s10482-007-9212-0

[20] Mukhopadhyay A, Redding AM, Joachimiak MP, Arkin AP, Borglin SE, Dehal PS, et al. Cell-wide responses to low-oxygen exposure in *Desulfovibrio vulgaris* Hildenborough. J Bacteriol. 2007;189:5996–6010. 17545284 10.1128/JB.00368-07 PMC1952033

[21] Wickner S, Nguyen T-LL, Genest O. The bacterial Hsp90 chaperone: cellular functions and mechanism of action. Annu Rev Microbiol. 2021;75:719–39. 34375543 10.1146/annurev-micro-032421-035644

[22] Genest O, Hoskins JR, Camberg JL, Doyle SM, Wickner S. Heat shock protein 90 from *Escherichia coli* collaborates with the DnaK chaperone system in client protein remodeling. Proc Natl Acad Sci. 2011;108:8206–11. 21525416 10.1073/pnas.1104703108 PMC3100916

[23] Rosenzweig R, Moradi S, Zarrine-Afsar A, Glover JR, Kay LE. Unraveling the mechanism of protein disaggregation through a ClpB-DnaK interaction. Science. 2013;339:1080–3. 23393091 10.1126/science.1233066

[24] Lund PA. Multiple chaperonins in bacteria – why so many? FEMS Microbiol Rev. 2009;33:785–800. 19416363 10.1111/j.1574-6976.2009.00178.x

[25] Susin MF, Baldini RL, Gueiros-Filho F, Gomes SL. GroES/GroEL and DnaK/DnaJ have distinct roles in stress responses and during cell cycle progression in *Caulobacter crescentus*. J Bacteriol. 2006;188:8044–53. 16980445 10.1128/JB.00824-06 PMC1698207

[26] Holmgren A. Thioredoxin. Annu Rev Biochem. 1985;54:237–71. 3896121 10.1146/annurev.bi.54.070185.001321

[27] Sarin R, Sharma YD. Thioredoxin system in obligate anaerobe *Desulfovibrio desulfuricans*: Identification and characterization of a novel thioredoxin 2. Gene. 2006;376:107–15. 16580795 10.1016/j.gene.2006.02.012

[28] Ezraty B, Gennaris A, Barras F, Collet J-F. Oxidative stress, protein damage and repair in bacteria. Nat Rev Microbiol. 2017;15:385–96. 28420885 10.1038/nrmicro.2017.26

[29] Cypionka H. Oxygen respiration by *Desulfovibrio* species. Annu Rev Microbiol. 2000;54:827–48. 11018146 10.1146/annurev.micro.54.1.827

[30] Limpens J, Berendse F, Blodau C, Canadell JG, Freeman C, Holden J, et al. Peatlands and the carbon cycle: from local processes to global implications – a synthesis. Biogeosciences. 2008;5:1475–91.

[31] Köchy M, Hiederer R, Freibauer A. Global distribution of soil organic carbon – Part 1: Masses and frequency distributions of SOC stocks for the tropics, permafrost regions, wetlands, and the world. SOIL. 2015;1:351–65.

[32] Kirschke S, Bousquet P, Ciais P, Saunois M, Canadell JG, Dlugokencky EJ, et al. Three decades of global methane sources and sinks. Nature Geosci. 2013;6:813–23.

[33] Saunois M, Bousquet P, Poulter B, Peregon A, Ciais P, Canadell JG, et al. The global methane budget 2000–2012. Earth Syst Sci Data. 2016;8:697–751.

[34] Loy A, Küsel K, Lehner A, Drake HL, Wagner M. Microarray and functional gene analyses of sulfate-reducing prokaryotes in low-sulfate, acidic fens reveal cooccurrence of recognized genera and novel lineages. Appl Environ Microbiol. 2004;70:6998–7009. 15574893 10.1128/AEM.70.12.6998-7009.2004 PMC535148

[35] Frei S, Knorr KH, Peiffer S, Fleckenstein JH. Surface micro-topography causes hot spots of biogeochemical activity in wetland systems: a virtual modeling experiment. J Geophys Res Biogeosci. 2012. 10.1029/2012JG002012.

[36] Pester M, Knorr K-H, Friedrich M, Wagner M, Loy A. Sulfate-reducing microorganisms in wetlands – fameless actors in carbon cycling and climate change. Front Microbiol. 2012;3:72. 10.3389/fmicb.2012.00072. 22403575 10.3389/fmicb.2012.00072 PMC3289269

[37] Knorr K-H, Blodau C. Impact of experimental drought and rewetting on redox transformations and methanogenesis in mesocosms of a northern fen soil. Soil Biol Biochem. 2009;41:1187–98.

[38] Helbig M, Waddington JM, Alekseychik P, Amiro BD, Aurela M, Barr AG, et al. Increasing contribution of peatlands to boreal evapotranspiration in a warming climate. Nat Clim Chang. 2020;10:555–60.

[39] Bertrand G, Ponçot A, Pohl B, Lhosmot A, Steinmann M, Johannet A, et al. Statistical hydrology for evaluating peatland water table sensitivity to simple environmental variables and climate changes application to the mid-latitude/altitude Frasne peatland (Jura Mountains, France). Sci Total Environ. 2021;754:141931. 33254862 10.1016/j.scitotenv.2020.141931

[40] Hamberger A, Horn MA, Dumont MG, Murrell JC, Drake HL. Anaerobic consumers of monosaccharides in a moderately acidic fen. Appl Environ Microbiol. 2008;74:3112–20. 18378662 10.1128/AEM.00193-08 PMC2394944

[41] Küsel K, Blöthe M, Schulz D, Reiche M, Drake HL. Microbial reduction of iron and porewater biogeochemistry in acidic peatlands. Biogeosciences. 2008;5:1537–49.

[42] Reiche M, Hädrich A, Lischeid G, Küsel K. Impact of manipulated drought and heavy rainfall events on peat mineralization processes and source-sink functions of an acidic fen. J Geophys Res Biogeosci. 2009. 10.1029/2008JG000853.

[43] Wüst PK, Horn MA, Drake HL. Trophic links between fermenters and methanogens in a moderately acidic fen soil. Environ Microbiol. 2009;11:1395–409. 19222542 10.1111/j.1462-2920.2009.01867.x

[44] Conrad R. Contribution of hydrogen to methane production and control of hydrogen concentrations in methanogenic soils and sediments. FEMS Microbiol Ecol. 1999;28:193–202.

[45] Gauci V, Matthews E, Dise N, Walter B, Koch D, Granberg G, et al. Sulfur pollution suppression of the wetland methane source in the 20th and 21st centuries. Proc Natl Acad Sci. 2004;101:12583–7. 15297612 10.1073/pnas.0404412101 PMC515100

[46] Hausmann B, Knorr K-H, Schreck K, Tringe SG, Glavina del Rio T, Loy A, et al. Consortia of low-abundance bacteria drive sulfate reduction-dependent degradation of fermentation products in peat soil microcosms. ISME J. 2016;10:2365–75. 27015005 10.1038/ismej.2016.42 PMC4930147

[47] Schmalenberger A, Drake HL, Küsel K. High unique diversity of sulfate-reducing prokaryotes characterized in a depth gradient in an acidic fen. Environ Microbiol. 2007;9:1317–28. 17472643 10.1111/j.1462-2920.2007.01251.x

[48] Steger D, Wentrup C, Braunegger C, Deevong P, Hofer M, Richter A, et al. Microorganisms with novel dissimilatory (bi)sulfite reductase genes are widespread and part of the core microbiota in low-sulfate peatlands. Appl Environ Microbiol. 2011;77:1231–42. 21169452 10.1128/AEM.01352-10 PMC3067207

[49] Schmidt O, Hink L, Horn MA, Drake HL. Peat: home to novel syntrophic species that feed acetate- and hydrogen-scavenging methanogens. ISME J. 2016;10:1954–66. 26771931 10.1038/ismej.2015.256 PMC5029166

[50] Pedrós-Alió C. The rare bacterial biosphere. Ann Rev Mar Sci. 2012;4:449–66. 22457983 10.1146/annurev-marine-120710-100948

[51] Lynch MDJ, Neufeld JD. Ecology and exploration of the rare biosphere. Nat Rev Microbiol. 2015;13:217–29. 25730701 10.1038/nrmicro3400

[52] Jousset A, Bienhold C, Chatzinotas A, Gallien L, Gobet A, Kurm V, et al. Where less may be more: how the rare biosphere pulls ecosystems strings. ISME J. 2017;11:853–62. 28072420 10.1038/ismej.2016.174 PMC5364357

[53] Pester M, Bittner N, Deevong P, Wagner M, Loy A. A ‘rare biosphere’ microorganism contributes to sulfate reduction in a peatland. ISME J. 2010;4:1591–602. 20535221 10.1038/ismej.2010.75 PMC4499578

[54] Hausmann B, Pelikan C, Rattei T, Loy A, Pester M. Long-term transcriptional activity at zero growth of a cosmopolitan rare biosphere member. mBio. 2019;10:e02189-18. 30755506 10.1128/mBio.02189-18 PMC6372793

[55] Dyksma S, Pester M. Oxygen respiration and polysaccharide degradation by a sulfate-reducing acidobacterium. Nat Commun. 2023;14:6337. 37816749 10.1038/s41467-023-42074-z PMC10564751

[56] Stoddard SF, Smith BJ, Hein R, Roller BRK, Schmidt TM. rrnDB: improved tools for interpreting rRNA gene abundance in bacteria and archaea and a new foundation for future development. Nucleic Acids Res. 2015;43:D593–8. 25414355 10.1093/nar/gku1201 PMC4383981

[57] Jain C, Rodriguez-R LM, Phillippy AM, Konstantinidis KT, Aluru S. High throughput ANI analysis of 90K prokaryotic genomes reveals clear species boundaries. Nat Commun. 2018;9:5114. 30504855 10.1038/s41467-018-07641-9 PMC6269478

[58] Zhao S, Ye Z, Stanton R. Misuse of RPKM or TPM normalization when comparing across samples and sequencing protocols. RNA. 2020;26:903–9. 32284352 10.1261/rna.074922.120 PMC7373998

[59] Sedano-Núñez VT, Boeren S, Stams AJM, Plugge CM. Comparative proteome analysis of propionate degradation by *Syntrophobacter fumaroxidans* in pure culture and in coculture with methanogens. Environ Microbiol. 2018;20:1842–56. 29611893 10.1111/1462-2920.14119 PMC5947623

[60] Ferreira D, Venceslau SS, Bernardino R, Preto A, Zhang L, Waldbauer JR, et al. DsrC is involved in fermentative growth and interacts directly with the FlxABCD–HdrABC complex in *Desulfovibrio vulgaris* Hildenborough. Environ Microbiol. 2023;25:962–76. 36602077 10.1111/1462-2920.16335

[61] Christensen GA, Zane GM, Kazakov AE, Li X, Rodionov DA, Novichkov PS, et al. Rex (encoded by DVU_0916) in *Desulfovibrio vulgaris* Hildenborough is a repressor of sulfate adenylyl transferase and is regulated by NADH. J Bacteriol. 2014;197:29–39. 25313388 10.1128/JB.02083-14 PMC4288696

[62] Cypionka H, Widdel F, Pfennig N. Survival of sulfate-reducing bacteria after oxygen stress, and growth in sulfate-free oxygen-sulfide gradients. FEMS Microbiol Ecol. 1985;1:39–45.

[63] Abdollahi H, Wimpenny JWT. Effects of oxygen on the growth of *Desulfovibrio desulfuricans*. Microbiology. 1990;136:1025–30.

[64] Marschall C, Frenzel P, Cypionka H. Influence of oxygen on sulfate reduction and growth of sulfate-reducing bacteria. Arch Microbiol. 1993;159:168–73.

[65] Johnson MS, Zhulin IB, Gapuzan ME, Taylor BL. Oxygen-dependent growth of the obligate anaerobe *Desulfovibrio vulgaris* Hildenborough. J Bacteriol. 1997;179:5598–601. 9287020 10.1128/jb.179.17.5598-5601.1997 PMC179436

[66] Lefèvre CT, Howse PA, Schmidt ML, Sabaty M, Menguy N, Luther GW III, et al. Growth of magnetotactic sulfate-reducing bacteria in oxygen concentration gradient medium. Environ Microbiol Rep. 2016;8:1003–15. 27701830 10.1111/1758-2229.12479

[67] Schoeffler M, Gaudin A-L, Ramel F, Valette O, Denis Y, Hania WB, et al. Growth of an anaerobic sulfate-reducing bacterium sustained by oxygen respiratory energy conservation after O_2_-driven experimental evolution. Environ Microbiol. 2019;21:360–73. 30394641 10.1111/1462-2920.14466

[68] Sigalevich P, Baev MV, Teske A, Cohen Y. Sulfate reduction and possible aerobic metabolism of the sulfate-reducing bacterium Desulfovibrio oxyclinae in a chemostat coculture with Marinobacter sp. strain MB under exposure to increasing oxygen concentrations. Applied and Environmental Microbiology. 2000;66:5013–8. 11055957 10.1128/aem.66.11.5013-5018.2000 PMC92413

[69] Silaghi-Dumitrescu R, Ng KY, Viswanathan R, Kurtz DM. A flavo-diiron protein from Desulfovibrio vulgaris with oxidase and nitric oxide reductase activities. Evidence for an in vivo nitric oxide scavenging function. Biochemistry. 2005;44:3572–9. 15736966 10.1021/bi0477337

[70] Dannenberg S, Kroder M, Dilling W, Cypionka H. Oxidation of H_2_, organic compounds and inorganic sulfur compounds coupled to reduction of O_2_ or nitrate by sulfate-reducing bacteria. Arch Microbiol. 1992;158:93–9.

[71] Lamrabet O, Pieulle L, Aubert C, Mouhamar F, Stocker P, Dolla A, et al. Oxygen reduction in the strict anaerobe *Desulfovibrio vulgaris* Hildenborough: characterization of two membrane-bound oxygen reductases. Microbiology. 2011;157:2720–32. 21737501 10.1099/mic.0.049171-0

[72] Lobo SAL, Almeida CC, Carita JN, Teixeira M, Saraiva LM. The haem–copper oxygen reductase of Desulfovibrio vulgaris contains a dihaem cytochrome c in subunit II. Biochim Biophys Acta. 2008;1777:1528–34. 18930018 10.1016/j.bbabio.2008.09.007

[73] Fournier M, Aubert C, Dermoun Z, Durand M-C, Moinier D, Dolla A. Response of the anaerobe *Desulfovibrio vulgaris* Hildenborough to oxidative conditions: proteome and transcript analysis. Biochimie. 2006;88:85–94. 16040186 10.1016/j.biochi.2005.06.012

[74] Zeller T, Klug G. Thioredoxins in bacteria: functions in oxidative stress response and regulation of thioredoxin genes. Naturwissenschaften. 2006;93:259–66. 16555095 10.1007/s00114-006-0106-1

[75] Marchant HK, Ahmerkamp S, Lavik G, Tegetmeyer HE, Graf J, Klatt JM, et al. Denitrifying community in coastal sediments performs aerobic and anaerobic respiration simultaneously. ISME J. 2017;11:1799–812. 28463234 10.1038/ismej.2017.51 PMC5520038

[76] Nguyen J, Lara-Gutiérrez J, Stocker R. Environmental fluctuations and their effects on microbial communities, populations and individuals. FEMS Microbiol Rev. 2021;45:fuaa068. 33338228 10.1093/femsre/fuaa068 PMC8371271

[77] Chen J, Hanke A, Tegetmeyer HE, Kattelmann I, Sharma R, Hamann E, et al. Impacts of chemical gradients on microbial community structure. ISME J. 2017;11:920–31. 28094795 10.1038/ismej.2016.175 PMC5363838

[78] Weiss RF. The solubility of nitrogen, oxygen and argon in water and seawater. Deep-Sea Res Oceanogr Abstr. 1970;17:721–35.

[79] Caporaso JG, Lauber CL, Walters WA, Berg-Lyons D, Lozupone CA, Turnbaugh PJ, et al. Global patterns of 16S rRNA diversity at a depth of millions of sequences per sample. Proc Natl Acad Sci. 2011;108:4516–22. 20534432 10.1073/pnas.1000080107 PMC3063599

[80] Apprill A, McNally S, Parsons R, Weber L. Minor revision to V4 region SSU rRNA 806R gene primer greatly increases detection of SAR11 bacterioplankton. Aquat Microb Ecol. 2015;75:129–37.

[81] Parada AE, Needham DM, Fuhrman JA. Every base matters: assessing small subunit rRNA primers for marine microbiomes with mock communities, time series and global field samples. Environ Microbiol. 2016;18:1403–14. 26271760 10.1111/1462-2920.13023

[82] Callahan BJ, McMurdie PJ, Rosen MJ, Han AW, Johnson AJA, Holmes SP. DADA2: High-resolution sample inference from Illumina amplicon data. Nat Methods. 2016;13:581–3. 27214047 10.1038/nmeth.3869 PMC4927377

[83] Bolyen E, Rideout JR, Dillon MR, Bokulich NA, Abnet CC, Al-Ghalith GA, et al. Reproducible, interactive, scalable and extensible microbiome data science using QIIME 2. Nat Biotechnol. 2019;37:852–7. 31341288 10.1038/s41587-019-0209-9 PMC7015180

[84] Pruesse E, Quast C, Knittel K, Fuchs BM, Ludwig W, Peplies J, et al. SILVA: a comprehensive online resource for quality checked and aligned ribosomal RNA sequence data compatible with ARB. Nucleic Acids Res. 2007;35:7188–96. 17947321 10.1093/nar/gkm864 PMC2175337

[85] Pruesse E, Peplies J, Glöckner FO. SINA: Accurate high-throughput multiple sequence alignment of ribosomal RNA genes. Bioinformatics. 2012;28:1823–9. 22556368 10.1093/bioinformatics/bts252 PMC3389763

[86] Bushnell B. BBTools software package. 2014.

[87] Li D, Liu C-M, Luo R, Sadakane K, Lam T-W. MEGAHIT: an ultra-fast single-node solution for large and complex metagenomics assembly via succinct de Bruijn graph. Bioinformatics. 2015;31:1674–6. 25609793 10.1093/bioinformatics/btv033

[88] Kang DD, Li F, Kirton E, Thomas A, Egan R, An H, et al. MetaBAT 2: an adaptive binning algorithm for robust and efficient genome reconstruction from metagenome assemblies. PeerJ. 2019;7:e7359. 31388474 10.7717/peerj.7359 PMC6662567

[89] Wu Y-W, Simmons BA, Singer SW. MaxBin 2.0: an automated binning algorithm to recover genomes from multiple metagenomic datasets. Bioinformatics. 2016;32:605–7. 26515820 10.1093/bioinformatics/btv638

[90] Mallawaarachchi V, Lin Y. MetaCoAG: binning metagenomic contigs via composition, coverage and assembly graphs. In: Pe’er I, editor. Research in computational molecular biology. Cham: Springer International Publishing; 2022. p. 70–85. 10.1089/cmb.2022.0262 36367700

[91] Sieber CMK, Probst AJ, Sharrar A, Thomas BC, Hess M, Tringe SG, et al. Recovery of genomes from metagenomes via a dereplication, aggregation and scoring strategy. Nat Microbiol. 2018;3:836–43. 29807988 10.1038/s41564-018-0171-1 PMC6786971

[92] Parks DH, Imelfort M, Skennerton CT, Hugenholtz P, Tyson GW. CheckM: assessing the quality of microbial genomes recovered from isolates, single cells, and metagenomes. Genome Res. 2015;25:1043–55. 25977477 10.1101/gr.186072.114 PMC4484387

[93] Chaumeil P-A, Mussig AJ, Hugenholtz P, Parks DH. GTDB-Tk: a toolkit to classify genomes with the Genome Taxonomy Database. Bioinformatics. 2020;36:1925–7. 10.1093/bioinformatics/btz848 PMC7703759 31730192

[94] Dong X, Strous M. An integrated pipeline for annotation and visualization of metagenomic contigs. Front Genet. 2019;10: 999. 31681429 10.3389/fgene.2019.00999 PMC6803454

[95] Cantalapiedra CP, Hernández-Plaza A, Letunic I, Bork P, Huerta-Cepas J. eggNOG-mapper v2: functional annotation, orthology assignments, and domain prediction at the metagenomic scale. Mol Biol Evol. 2021;38:5825–9. 34597405 10.1093/molbev/msab293 PMC8662613

[96] Zhou Z, Tran PQ, Breister AM, Liu Y, Kieft K, Cowley ES, et al. METABOLIC: high-throughput profiling of microbial genomes for functional traits, metabolism, biogeochemistry, and community-scale functional networks. Microbiome. 2022;10:33. 35172890 10.1186/s40168-021-01213-8 PMC8851854

[97] Neukirchen S, Sousa FL. DiSCo: a sequence-based type-specific predictor of Dsr-dependent dissimilatory sulphur metabolism in microbial data. Microb Genom. 2021;7:000603. 34241589 10.1099/mgen.0.000603 PMC8477390

[98] Altschul SF, Gish W, Miller W, Myers EW, Lipman DJ. Basic local alignment search tool. J Mol Biol. 1990;215:403–10. 2231712 10.1016/S0022-2836(05)80360-2

[99] Bairoch A, Apweiler R. The SWISS-PROT protein sequence database and its supplement TrEMBL in 2000. Nucleic Acids Res. 2000;28:45–8. 10592178 10.1093/nar/28.1.45 PMC102476

[100] Pruitt KD, Tatusova T, Maglott DR. NCBI Reference Sequence (RefSeq): a curated non-redundant sequence database of genomes, transcripts and proteins. Nucleic Acids Res. 2005;33:D501–4. 15608248 10.1093/nar/gki025 PMC539979

[101] Katoh K, Misawa K, Kuma K, Miyata T. MAFFT: a novel method for rapid multiple sequence alignment based on fast Fourier transform. Nucleic Acids Res. 2002;30:3059–66. 12136088 10.1093/nar/gkf436 PMC135756

[102] Minh BQ, Schmidt HA, Chernomor O, Schrempf D, Woodhams MD, von Haeseler A, et al. IQ-TREE 2: new models and efficient methods for phylogenetic inference in the genomic era. Mol Biol Evol. 2020;37:1530–4. 32011700 10.1093/molbev/msaa015 PMC7182206

[103] Sunagawa S, Mende DR, Zeller G, Izquierdo-Carrasco F, Berger SA, Kultima JR, et al. Metagenomic species profiling using universal phylogenetic marker genes. Nat Methods. 2013;10:1196–9. 24141494 10.1038/nmeth.2693

[104] Parks DH, Chuvochina M, Waite DW, Rinke C, Skarshewski A, Chaumeil P-A, et al. A standardized bacterial taxonomy based on genome phylogeny substantially revises the tree of life. Nat Biotechnol. 2018;36:996–1004. 30148503 10.1038/nbt.4229

[105] Deng Z-L, Münch PC, Mreches R, McHardy AC. Rapid and accurate identification of ribosomal RNA sequences via deep learning. Nucleic Acids Res. 2022;50:e60. 35188571 10.1093/nar/gkac112 PMC9177968

[106] Robinson MD, McCarthy DJ, Smyth GK. edgeR: a Bioconductor package for differential expression analysis of digital gene expression data. Bioinformatics. 2010;26:139–40. 19910308 10.1093/bioinformatics/btp616 PMC2796818

[107] Love MI, Huber W, Anders S. Moderated estimation of fold change and dispersion for RNA-seq data with DESeq2. Genome Biol. 2014;15:550. 25516281 10.1186/s13059-014-0550-8 PMC4302049

