# Supplementary figures and images for: Growth of sulfate-reducing Desulfobacterota and Bacillota at periodic oxygen stress of 50% air-O2 saturation
Source: Microbiome. 2024 Oct 4;12:191. doi: 10.1186/s40168-024-01909-7 (PMC11451228; doi:10.1186/s40168-024-01909-7)

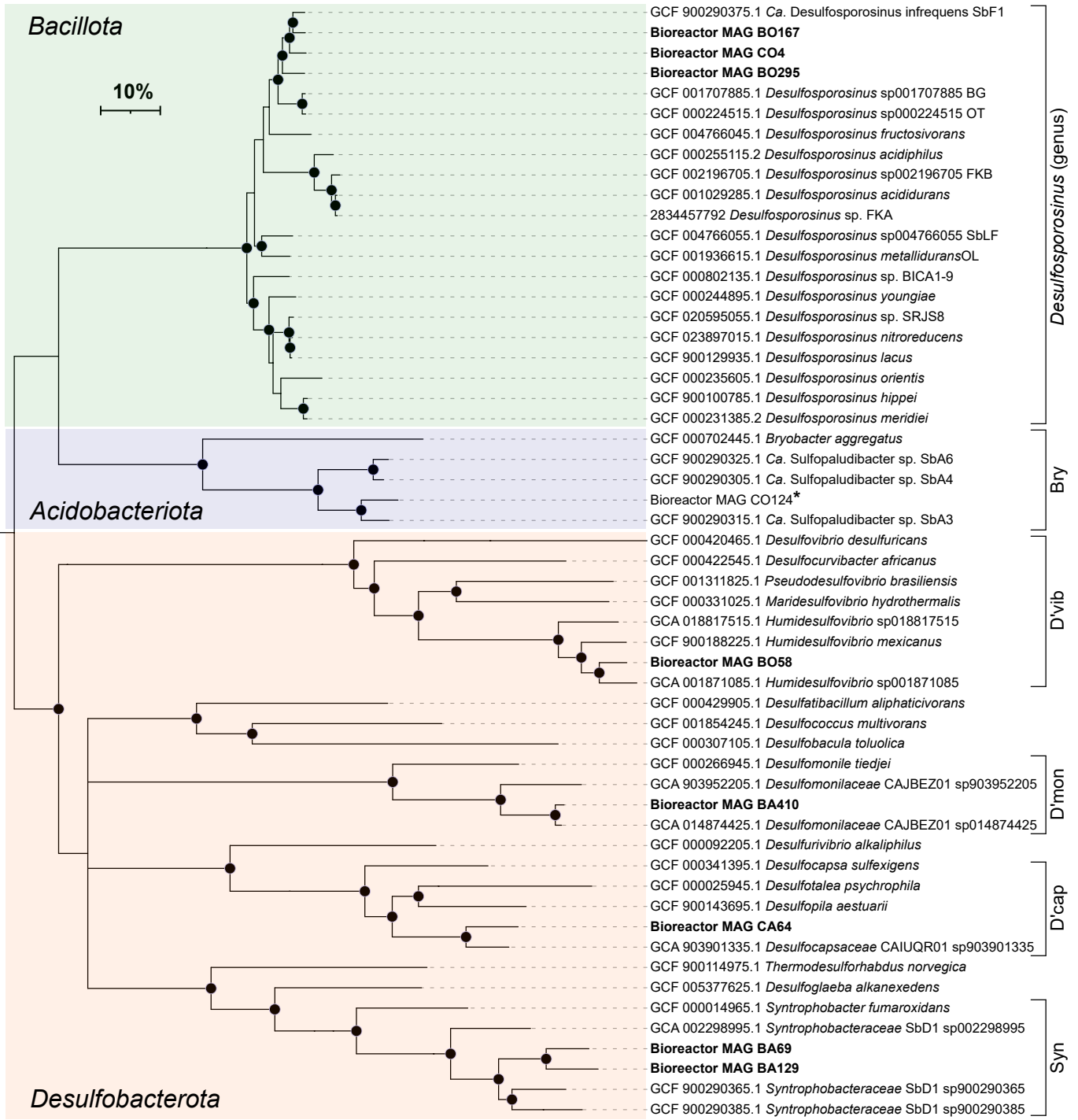

Supplement: Supplementary file 2 — Supplementary Material 1: Supplementary Figure S1. Maximum likelihood phylogenomic reconstruction of all metagenome assembled genomes (MAGs) that affiliated with sulfate-reducing bacteria recovered from the bioreactor (bold). The tree was constructed based on concatenated amino acid alignment deduced from 120 single copy marker genes [104]. Bootstrap support ≥90% is indicated by black dots. The asterisk denotes a facultatively anaerobic, sulfate-reducing Acidobacteriota representative that has been published elsewhere [55]. Abbreviations of the families are as follows: Bry, Bryobacteraceae; D’vib, Desulfovibrionaceae; D’mon, Desulfomonilaceae; D’cap, Desulfocapsaceae; Syn, Syntrophobacteraceae. [file 40168_2024_1909_MOESM1_ESM.pdf]
