# Supplementary material for: Growth of sulfate-reducing Desulfobacterota and Bacillota at periodic oxygen stress of 50% air-O2 saturation
Source: Microbiome. 2024 Oct 4;12:191. doi: 10.1186/s40168-024-01909-7 (PMC11451228; doi:10.1186/s40168-024-01909-7)
Supplement: Supplementary file 3 — Supplementary Material 2: Supplementary Figure S2. Maximum likelihood phylogeny of selected oxygen defense proteins identified in the eight SRB-MAGs. MAG-derived sequences are highlighted in blue. Manually annotated and reviewed reference protein sequences collected from the Swiss-Prot database are highlighted in red. All trees were midpoint rooted and bootstrap support ≥ 90% is indicated by black dots. [file 40168_2024_1909_MOESM2_ESM.pdf]

Roo/NorV

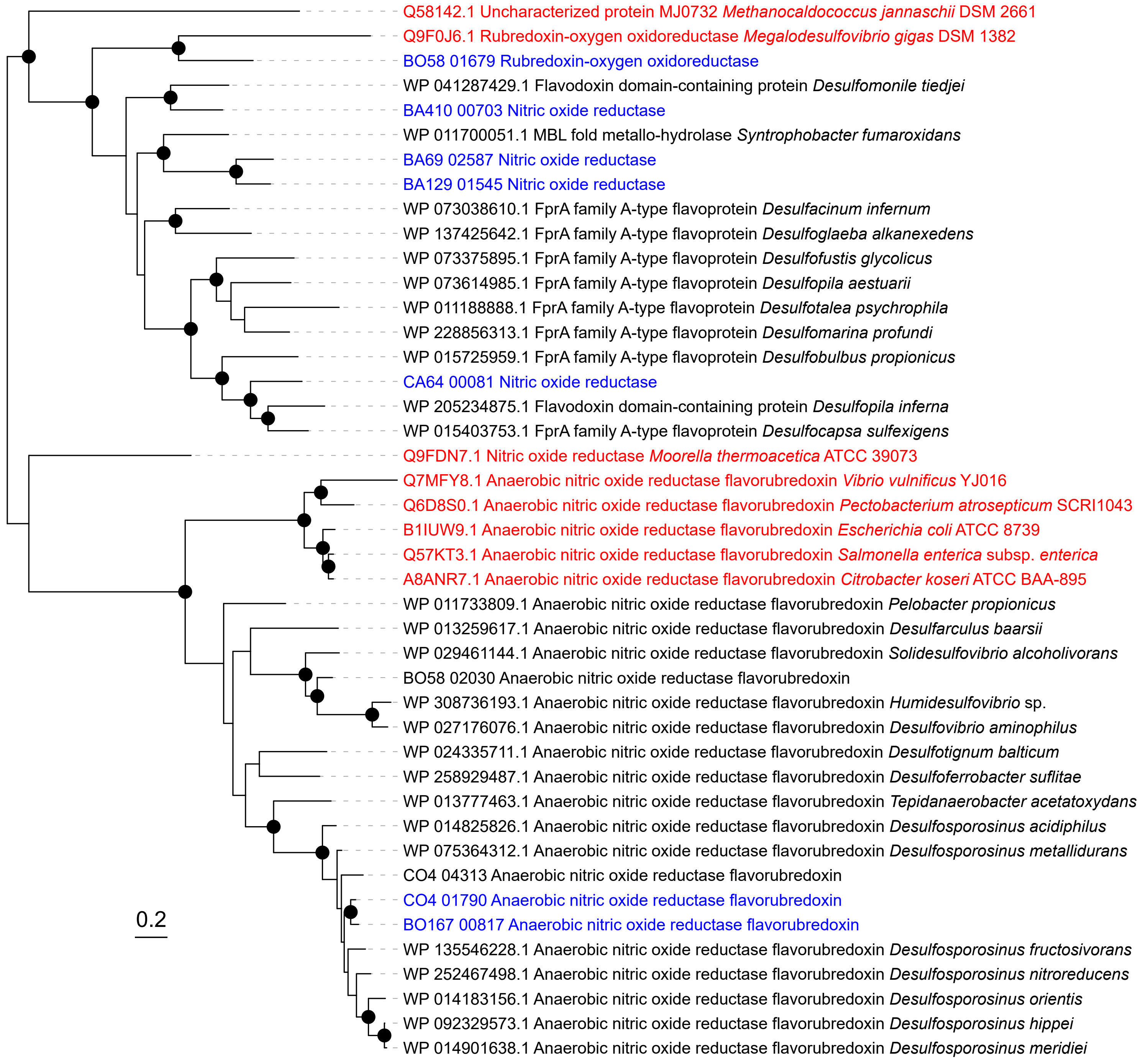
Q58142.1 Uncharacterized protein MJ0732 *Methanocaldococcus jannaschii* DSM 2661
Q9F0J6.1 Rubredoxin-oxygen oxidoreductase *Megalodesulfovibrio gigas* DSM 1382
BO58 01679 Rubredoxin-oxygen oxidoreductase
WP 041287429.1 Flavodoxin domain-containing protein *Desulfomonile tiedjei*
BA410 00703 Nitric oxide reductase
WP 011700051.1 MBL fold metallo-hydrolase *Syntrophobacter fumaroxidans*
BA69 02587 Nitric oxide reductase
BA129 01545 Nitric oxide reductase
WP 073038610.1 FprA family A-type flavoprotein *Desulfacinum infernum*
WP 137425642.1 FprA family A-type flavoprotein *Desulfoglaeba alkanexedens*
WP 073375895.1 FprA family A-type flavoprotein *Desulfofustis glycolicus*
WP 073614985.1 FprA family A-type flavoprotein *Desulfopila aestuarii*
WP 011188888.1 FprA family A-type flavoprotein *Desulfotalea psychrophila*
WP 228856313.1 FprA family A-type flavoprotein *Desulfomarina profundi*
WP 015725959.1 FprA family A-type flavoprotein *Desulfobulbus propionicus*
CA64 00081 Nitric oxide reductase
WP 205234875.1 Flavodoxin domain-containing protein *Desulfopila inferna*
WP 015403753.1 FprA family A-type flavoprotein *Desulfocapsa sulfexigens*
Q9FDN7.1 Nitric oxide reductase *Moorella thermoacetica* ATCC 39073
Q7MFY8.1 Anaerobic nitric oxide reductase flavorubredoxin *Vibrio vulnificus* YJ016
Q6D8S0.1 Anaerobic nitric oxide reductase flavorubredoxin *Pectobacterium atrosepticum* SCRI1043
B1IUW9.1 Anaerobic nitric oxide reductase flavorubredoxin *Escherichia coli* ATCC 8739
Q57KT3.1 Anaerobic nitric oxide reductase flavorubredoxin *Salmonella enterica* subsp. *enterica*
A8ANR7.1 Anaerobic nitric oxide reductase flavorubredoxin *Citrobacter koseri* ATCC BAA-895
WP 011733809.1 Anaerobic nitric oxide reductase flavorubredoxin *Pelobacter propionicus*
WP 013259617.1 Anaerobic nitric oxide reductase flavorubredoxin *Desulfarculus baarsii*
WP 029461144.1 Anaerobic nitric oxide reductase flavorubredoxin *Solidesulfovibrio alcoholivorans*
BO58 02030 Anaerobic nitric oxide reductase flavorubredoxin
WP 308736193.1 Anaerobic nitric oxide reductase flavorubredoxin *Humidesulfovibrio* sp.
WP 027176076.1 Anaerobic nitric oxide reductase flavorubredoxin *Desulfovibrio aminophilus*
WP 024335711.1 Anaerobic nitric oxide reductase flavorubredoxin *Desulfotignum balticum*
WP 258929487.1 Anaerobic nitric oxide reductase flavorubredoxin *Desulfoferrobacter suflitae*
WP 013777463.1 Anaerobic nitric oxide reductase flavorubredoxin *Tepidanaerobacter acetatoxydans*
WP 014825826.1 Anaerobic nitric oxide reductase flavorubredoxin *Desulfosporosinus acidiphilus*
WP 075364312.1 Anaerobic nitric oxide reductase flavorubredoxin *Desulfosporosinus metallidurans*
CO4 04313 Anaerobic nitric oxide reductase flavorubredoxin
CO4 01790 Anaerobic nitric oxide reductase flavorubredoxin
BO167 00817 Anaerobic nitric oxide reductase flavorubredoxin
WP 135546228.1 Anaerobic nitric oxide reductase flavorubredoxin *Desulfosporosinus fructosivorans*
WP 252467498.1 Anaerobic nitric oxide reductase flavorubredoxin *Desulfosporosinus nitroreducens*
WP 014183156.1 Anaerobic nitric oxide reductase flavorubredoxin *Desulfosporosinus orientis*
WP 092329573.1 Anaerobic nitric oxide reductase flavorubredoxin *Desulfosporosinus hippei*
WP 014901638.1 Anaerobic nitric oxide reductase flavorubredoxin *Desulfosporosinus meridiei*
0.2

CydA

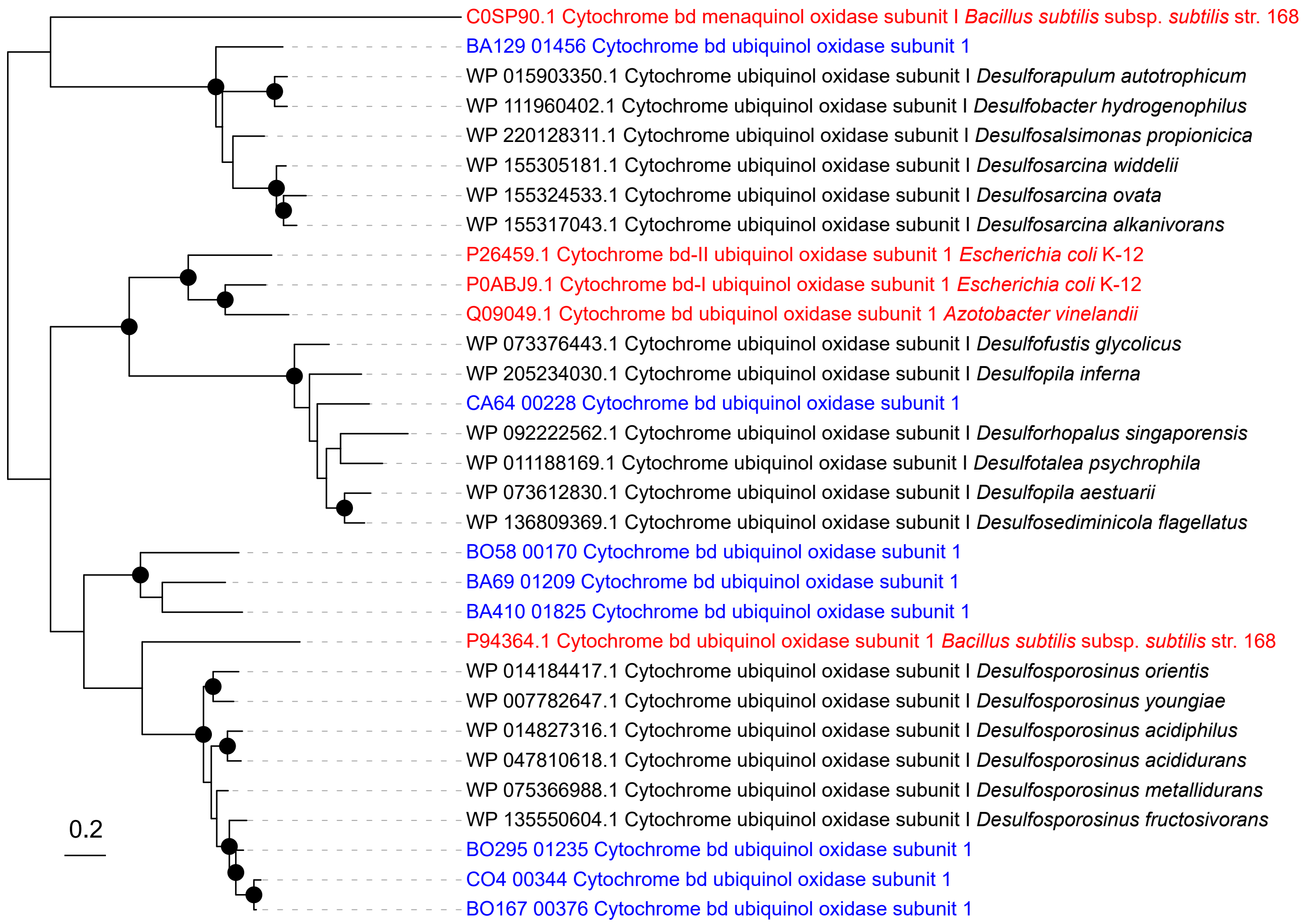

C0SP90.1 Cytochrome bd menaquinol oxidase subunit I *Bacillus subtilis* subsp. *subtilis* str. 168
BA129 01456 Cytochrome bd ubiquinol oxidase subunit 1
WP 015903350.1 Cytochrome ubiquinol oxidase subunit I *Desulforapulum autotrophicum*
WP 111960402.1 Cytochrome ubiquinol oxidase subunit I *Desulfobacter hydrogenophilus*
WP 220128311.1 Cytochrome ubiquinol oxidase subunit I *Desulfosalsimonas propionicica*
WP 155305181.1 Cytochrome ubiquinol oxidase subunit I *Desulfosarcina widdelii*
WP 155324533.1 Cytochrome ubiquinol oxidase subunit I *Desulfosarcina ovata*
WP 155317043.1 Cytochrome ubiquinol oxidase subunit I *Desulfosarcina alkanivorans*
P26459.1 Cytochrome bd-II ubiquinol oxidase subunit 1 *Escherichia coli* K-12
P0ABJ9.1 Cytochrome bd-I ubiquinol oxidase subunit 1 *Escherichia coli* K-12
Q09049.1 Cytochrome bd ubiquinol oxidase subunit 1 *Azotobacter vinelandii*
WP 073376443.1 Cytochrome ubiquinol oxidase subunit I *Desulfofustis glycolicus*
WP 205234030.1 Cytochrome ubiquinol oxidase subunit I *Desulfopila inferna*
CA64 00228 Cytochrome bd ubiquinol oxidase subunit 1
WP 092222562.1 Cytochrome ubiquinol oxidase subunit I *Desulforhopalus singaporensis*
WP 011188169.1 Cytochrome ubiquinol oxidase subunit I *Desulfotalea psychrophila*
WP 073612830.1 Cytochrome ubiquinol oxidase subunit I *Desulfopila aestuarii*
WP 136809369.1 Cytochrome ubiquinol oxidase subunit I *Desulfosediminicola flagellatus*
BO58 00170 Cytochrome bd ubiquinol oxidase subunit 1
BA69 01209 Cytochrome bd ubiquinol oxidase subunit 1
BA410 01825 Cytochrome bd ubiquinol oxidase subunit 1
P94364.1 Cytochrome bd ubiquinol oxidase subunit 1 *Bacillus subtilis* subsp. *subtilis* str. 168
WP 014184417.1 Cytochrome ubiquinol oxidase subunit I *Desulfosporosinus orientis*
WP 007782647.1 Cytochrome ubiquinol oxidase subunit I *Desulfosporosinus youngiae*
WP 014827316.1 Cytochrome ubiquinol oxidase subunit I *Desulfosporosinus acidiphilus*
WP 047810618.1 Cytochrome ubiquinol oxidase subunit I *Desulfosporosinus acididurans*
WP 075366988.1 Cytochrome ubiquinol oxidase subunit I *Desulfosporosinus metallidurans*
WP 135550604.1 Cytochrome ubiquinol oxidase subunit I *Desulfosporosinus fructosivorans*
BO295 01235 Cytochrome bd ubiquinol oxidase subunit 1
CO4 00344 Cytochrome bd ubiquinol oxidase subunit 1
BO167 00376 Cytochrome bd ubiquinol oxidase subunit 1
0.2

CydB

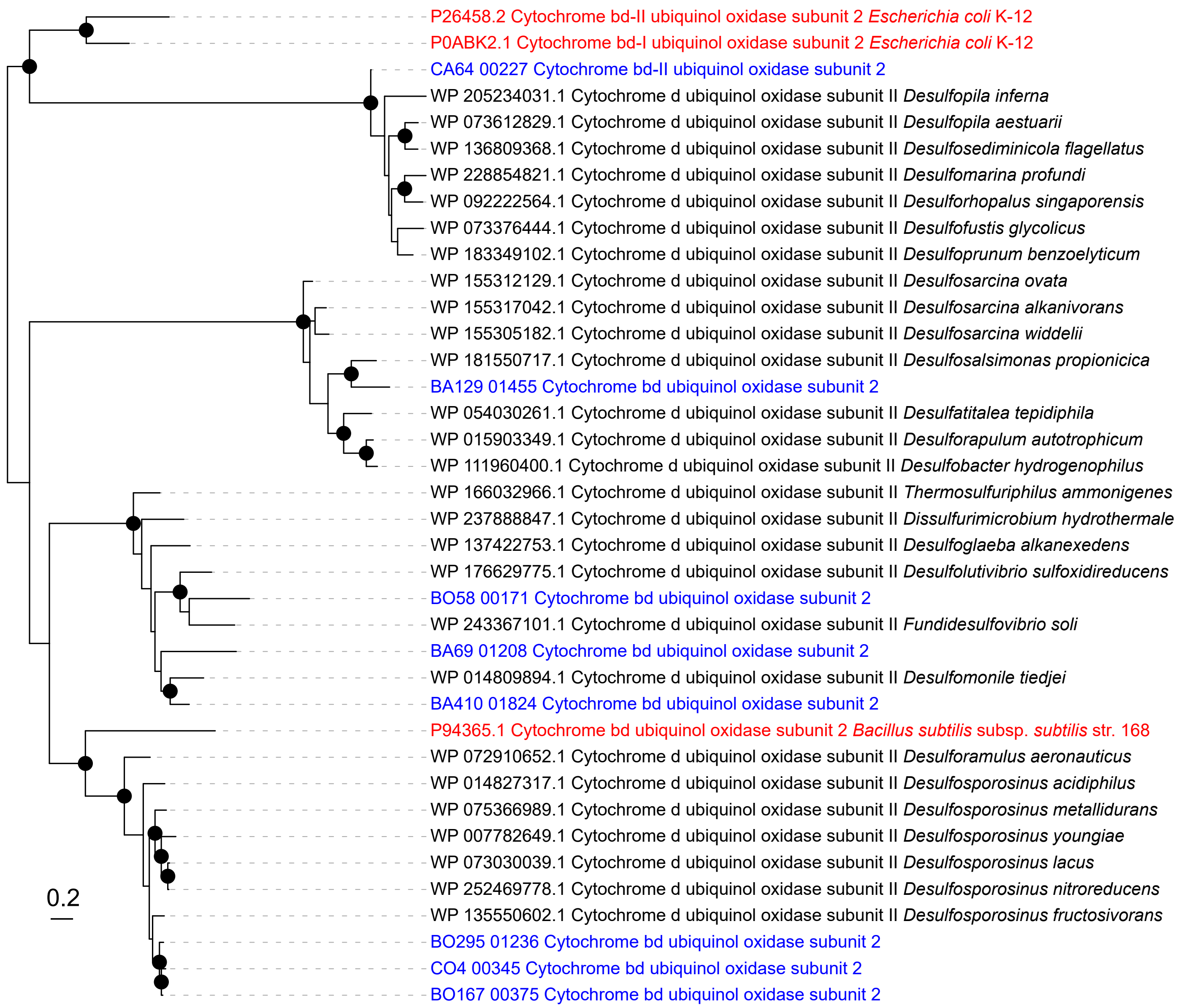
P26458.2 Cytochrome bd-II ubiquinol oxidase subunit 2 *Escherichia coli* K-12
P0ABK2.1 Cytochrome bd-I ubiquinol oxidase subunit 2 *Escherichia coli* K-12
CA64 00227 Cytochrome bd-II ubiquinol oxidase subunit 2
WP 205234031.1 Cytochrome d ubiquinol oxidase subunit II *Desulfopila inferna*
WP 073612829.1 Cytochrome d ubiquinol oxidase subunit II *Desulfopila aestuarii*
WP 136809368.1 Cytochrome d ubiquinol oxidase subunit II *Desulfosediminicola flagellatus*
WP 228854821.1 Cytochrome d ubiquinol oxidase subunit II *Desulfomarina profundi*
WP 092222564.1 Cytochrome d ubiquinol oxidase subunit II *Desulforhopalus singaporensis*
WP 073376444.1 Cytochrome d ubiquinol oxidase subunit II *Desulfofustis glycolicus*
WP 183349102.1 Cytochrome d ubiquinol oxidase subunit II *Desulfoprunum benzoelyticum*
WP 155312129.1 Cytochrome d ubiquinol oxidase subunit II *Desulfosarcina ovata*
WP 155317042.1 Cytochrome d ubiquinol oxidase subunit II *Desulfosarcina alkanivorans*
WP 155305182.1 Cytochrome d ubiquinol oxidase subunit II *Desulfosarcina widdelii*
WP 181550717.1 Cytochrome d ubiquinol oxidase subunit II *Desulfosalsimonas propionicica*
BA129 01455 Cytochrome bd ubiquinol oxidase subunit 2
WP 054030261.1 Cytochrome d ubiquinol oxidase subunit II *Desulfatitalea tepidiphila*
WP 015903349.1 Cytochrome d ubiquinol oxidase subunit II *Desulforapulum autotrophicum*
WP 111960400.1 Cytochrome d ubiquinol oxidase subunit II *Desulfobacter hydrogenophilus*
WP 166032966.1 Cytochrome d ubiquinol oxidase subunit II *Thermosulfuriphilus ammonigenes*
WP 237888847.1 Cytochrome d ubiquinol oxidase subunit II *Dissulfurimicrobium hydrothermale*
WP 137422753.1 Cytochrome d ubiquinol oxidase subunit II *Desulfoglaeba alkanexedens*
WP 176629775.1 Cytochrome d ubiquinol oxidase subunit II *Desulfolutivibrio sulfoxidireducens*
BO58 00171 Cytochrome bd ubiquinol oxidase subunit 2
WP 243367101.1 Cytochrome d ubiquinol oxidase subunit II *Fundidesulfovibrio soli*
BA69 01208 Cytochrome bd ubiquinol oxidase subunit 2
WP 014809894.1 Cytochrome d ubiquinol oxidase subunit II *Desulfomonile tiedjei*
BA410 01824 Cytochrome bd ubiquinol oxidase subunit 2
P94365.1 Cytochrome bd ubiquinol oxidase subunit 2 *Bacillus subtilis* subsp. *subtilis* str. 168
WP 072910652.1 Cytochrome d ubiquinol oxidase subunit II *Desulforamulus aeronauticus*
WP 014827317.1 Cytochrome d ubiquinol oxidase subunit II *Desulfosporosinus acidiphilus*
WP 075366989.1 Cytochrome d ubiquinol oxidase subunit II *Desulfosporosinus metallidurans*
WP 007782649.1 Cytochrome d ubiquinol oxidase subunit II *Desulfosporosinus youngiae*
WP 073030039.1 Cytochrome d ubiquinol oxidase subunit II *Desulfosporosinus lacus*
WP 252469778.1 Cytochrome d ubiquinol oxidase subunit II *Desulfosporosinus nitroreducens*
WP 135550602.1 Cytochrome d ubiquinol oxidase subunit II *Desulfosporosinus fructosivorans*
BO295 01236 Cytochrome bd ubiquinol oxidase subunit 2
CO4 00345 Cytochrome bd ubiquinol oxidase subunit 2
BO167 00375 Cytochrome bd ubiquinol oxidase subunit 2
0.2

SodC

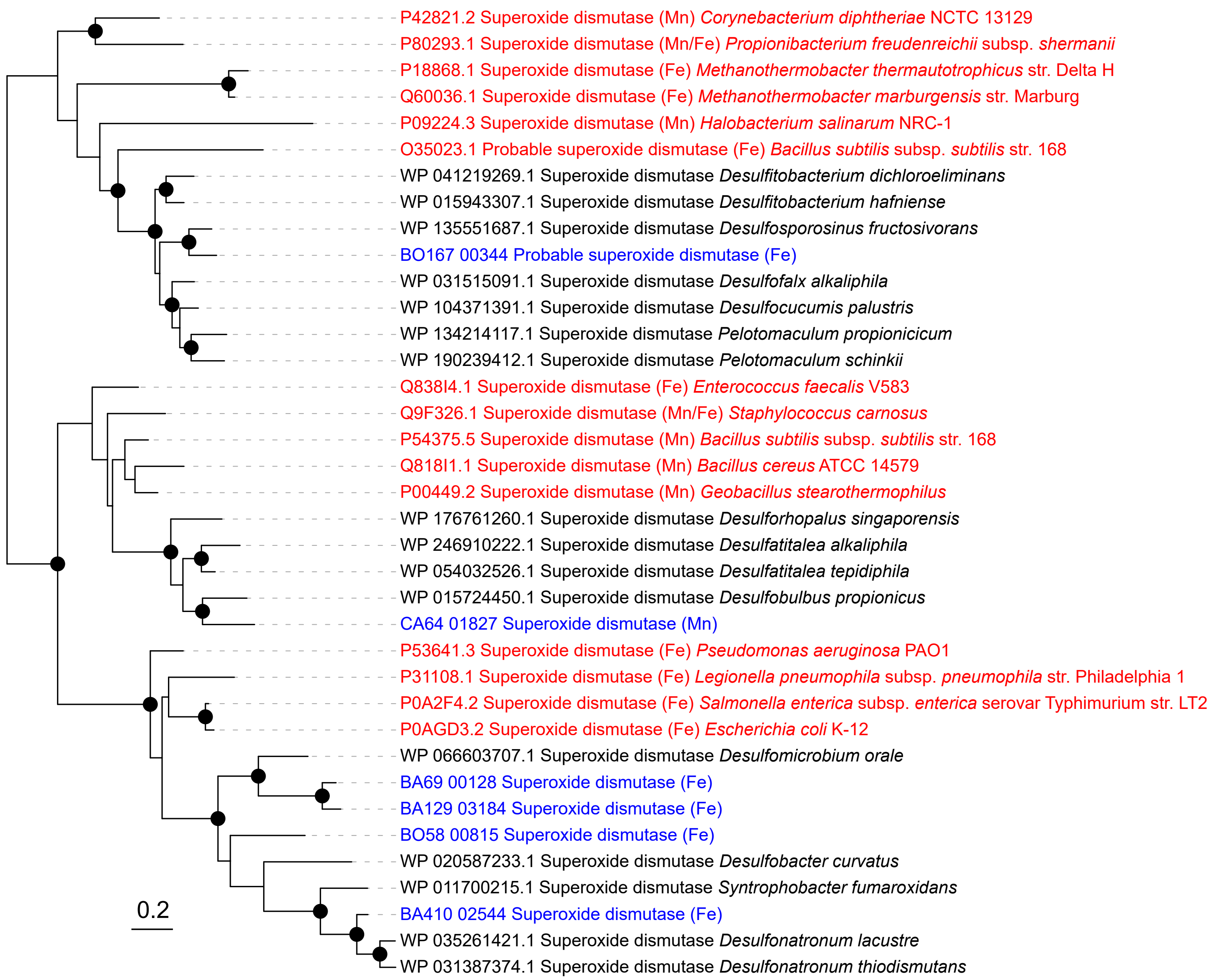

P42821.2 Superoxide dismutase (Mn) *Corynebacterium diphtheriae* NCTC 13129
P80293.1 Superoxide dismutase (Mn/Fe) *Propionibacterium freudenreichii* subsp. *shermanii*
P18868.1 Superoxide dismutase (Fe) *Methanothermobacter thermautotrophicus* str. Delta H
Q60036.1 Superoxide dismutase (Fe) *Methanothermobacter marburgensis* str. Marburg
P09224.3 Superoxide dismutase (Mn) *Halobacterium salinarum* NRC-1
O35023.1 Probable superoxide dismutase (Fe) *Bacillus subtilis* subsp. *subtilis* str. 168
WP 041219269.1 Superoxide dismutase *Desulfitobacterium dichloroeliminans*
WP 015943307.1 Superoxide dismutase *Desulfitobacterium hafniense*
WP 135551687.1 Superoxide dismutase *Desulfosporosinus fructosivorans*
BO167 00344 Probable superoxide dismutase (Fe)
WP 031515091.1 Superoxide dismutase *Desulfofalx alkaliphila*
WP 104371391.1 Superoxide dismutase *Desulfocucumis palustris*
WP 134214117.1 Superoxide dismutase *Pelotomaculum propionicicum*
WP 190239412.1 Superoxide dismutase *Pelotomaculum schinkii*
Q838I4.1 Superoxide dismutase (Fe) *Enterococcus faecalis* V583
Q9F326.1 Superoxide dismutase (Mn/Fe) *Staphylococcus carnosus*
P54375.5 Superoxide dismutase (Mn) *Bacillus subtilis* subsp. *subtilis* str. 168
Q818I1.1 Superoxide dismutase (Mn) *Bacillus cereus* ATCC 14579
P00449.2 Superoxide dismutase (Mn) *Geobacillus stearothermophilus*
WP 176761260.1 Superoxide dismutase *Desulforhopalus singaporensis*
WP 246910222.1 Superoxide dismutase *Desulfatitalea alkaliphila*
WP 054032526.1 Superoxide dismutase *Desulfatitalea tepidiphila*
WP 015724450.1 Superoxide dismutase *Desulfobulbus propionicus*
CA64 01827 Superoxide dismutase (Mn)
P53641.3 Superoxide dismutase (Fe) *Pseudomonas aeruginosa* PAO1
P31108.1 Superoxide dismutase (Fe) *Legionella pneumophila* subsp. *pneumophila* str. Philadelphia 1
P0A2F4.2 Superoxide dismutase (Fe) *Salmonella enterica* subsp. *enterica* serovar Typhimurium str. LT2
P0AGD3.2 Superoxide dismutase (Fe) *Escherichia coli* K-12
WP 066603707.1 Superoxide dismutase *Desulfomicrobium orale*
BA69 00128 Superoxide dismutase (Fe)
BA129 03184 Superoxide dismutase (Fe)
BO58 00815 Superoxide dismutase (Fe)
WP 020587233.1 Superoxide dismutase *Desulfobacter curvatus*
WP 011700215.1 Superoxide dismutase *Syntrophobacter fumaroxidans*
BA410 02544 Superoxide dismutase (Fe)
WP 035261421.1 Superoxide dismutase *Desulfonatronum lacustre*
WP 031387374.1 Superoxide dismutase *Desulfonatronum thiodismutans*
0.2

KatG

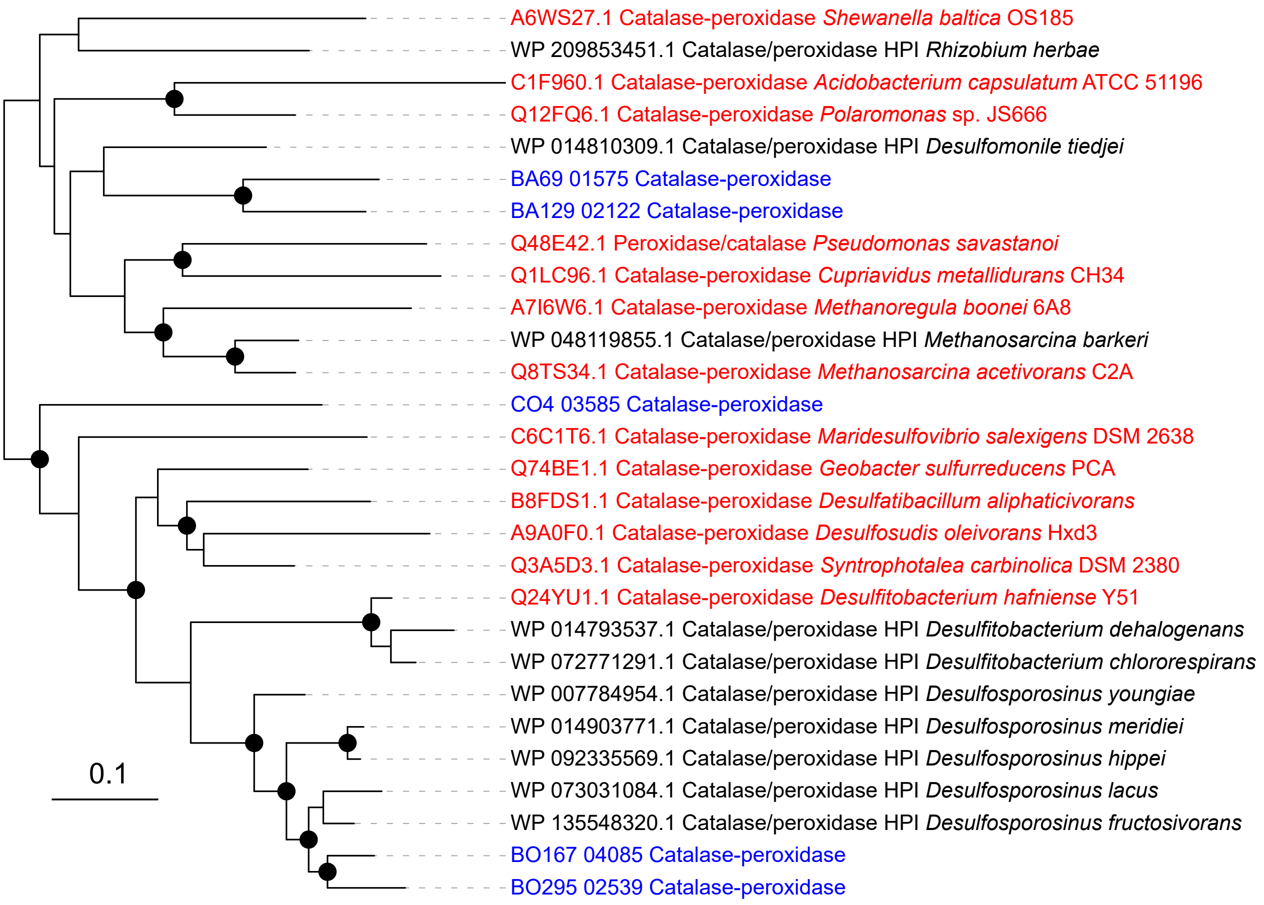
A6WS27.1 Catalase-peroxidase Shewanella baltica OS185
WP 209853451.1 Catalase/peroxidase HPI Rhizobium herbae
C1F960.1 Catalase-peroxidase Acidobacterium capsulatum ATCC 51196
Q12FQ6.1 Catalase-peroxidase Polaromonas sp. JS666
WP 014810309.1 Catalase/peroxidase HPI Desulfomonile tiedjei
BA69 01575 Catalase-peroxidase
BA129 02122 Catalase-peroxidase
Q48E42.1 Peroxidase/catalase Pseudomonas savastanoi
Q1LC96.1 Catalase-peroxidase Cupriavidus metallidurans CH34
A7I6W6.1 Catalase-peroxidase Methanoregula boonei 6A8
WP 048119855.1 Catalase/peroxidase HPI Methanosarcina barkeri
Q8TS34.1 Catalase-peroxidase Methanosarcina acetivorans C2A
CO4 03585 Catalase-peroxidase
C6C1T6.1 Catalase-peroxidase Maridesulfovibrio salexigens DSM 2638
Q74BE1.1 Catalase-peroxidase Geobacter sulfurreducens PCA
B8FDS1.1 Catalase-peroxidase Desulfatibacillum aliphaticivorans
A9A0F0.1 Catalase-peroxidase Desulfosudis oleivorans Hxd3
Q3A5D3.1 Catalase-peroxidase Syntrophotalea carbinolica DSM 2380
Q24YU1.1 Catalase-peroxidase Desulfitobacterium hafniense Y51
WP 014793537.1 Catalase/peroxidase HPI Desulfitobacterium dehalogenans
WP 072771291.1 Catalase/peroxidase HPI Desulfitobacterium chlororespirans
WP 007784954.1 Catalase/peroxidase HPI Desulfosporosinus youngiae
WP 014903771.1 Catalase/peroxidase HPI Desulfosporosinus meridiei
WP 092335569.1 Catalase/peroxidase HPI Desulfosporosinus hippei
WP 073031084.1 Catalase/peroxidase HPI Desulfosporosinus lacus
WP 135548320.1 Catalase/peroxidase HPI Desulfosporosinus fructosivorans
BO167 04085 Catalase-peroxidase
BO295 02539 Catalase-peroxidase
0.1

KatA

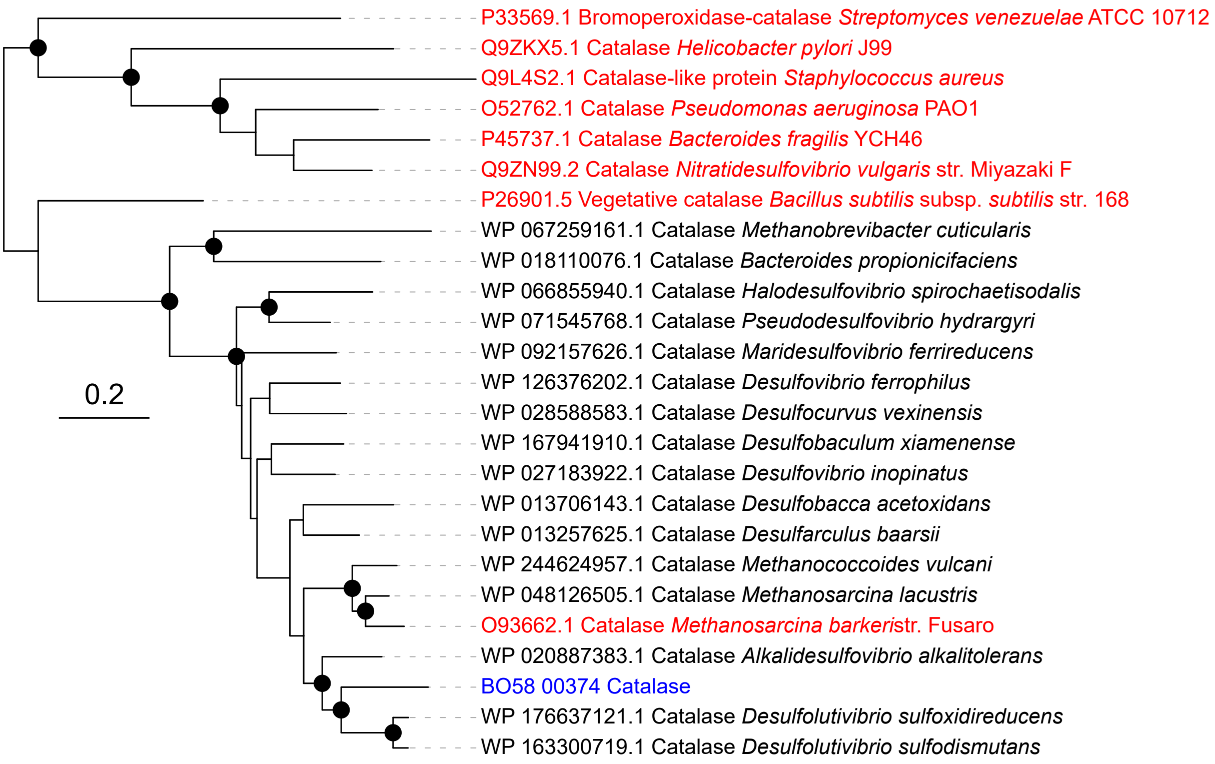
P33569.1 Bromoperoxidase-catalase Streptomyces venezuelae ATCC 10712
Q9ZKX5.1 Catalase Helicobacter pylori J99
Q9L4S2.1 Catalase-like protein Staphylococcus aureus
O52762.1 Catalase Pseudomonas aeruginosa PAO1
P45737.1 Catalase Bacteroides fragilis YCH46
Q9ZN99.2 Catalase Nitratidesulfovibrio vulgaris str. Miyazaki F
P26901.5 Vegetative catalase Bacillus subtilis subsp. subtilis str. 168
WP 067259161.1 Catalase Methanobrevibacter cuticularis
WP 018110076.1 Catalase Bacteroides propionicifaciens
WP 066855940.1 Catalase Halodesulfovibrio spirochaetisodalis
WP 071545768.1 Catalase Pseudodesulfovibrio hydrargyri
WP 092157626.1 Catalase Maridesulfovibrio ferrireducens
WP 126376202.1 Catalase Desulfovibrio ferrophilus
WP 028588583.1 Catalase Desulfocurvus vexinensis
WP 167941910.1 Catalase Desulfobaculum xiamenense
WP 027183922.1 Catalase Desulfovibrio inopinatus
WP 013706143.1 Catalase Desulfobacca acetoxidans
WP 013257625.1 Catalase Desulfarculus baarsii
WP 244624957.1 Catalase Methanococcoides vulcani
WP 048126505.1 Catalase Methanosarcina lacustris
O93662.1 Catalase Methanosarcina barkeristr. Fusaro
WP 020887383.1 Catalase Alkalidesulfovibrio alkalitolerans
BO58 00374 Catalase
WP 176637121.1 Catalase Desulfolutivibrio sulfoxidireducens
WP 163300719.1 Catalase Desulfolutivibrio sulfodismutans
0.2

PerR

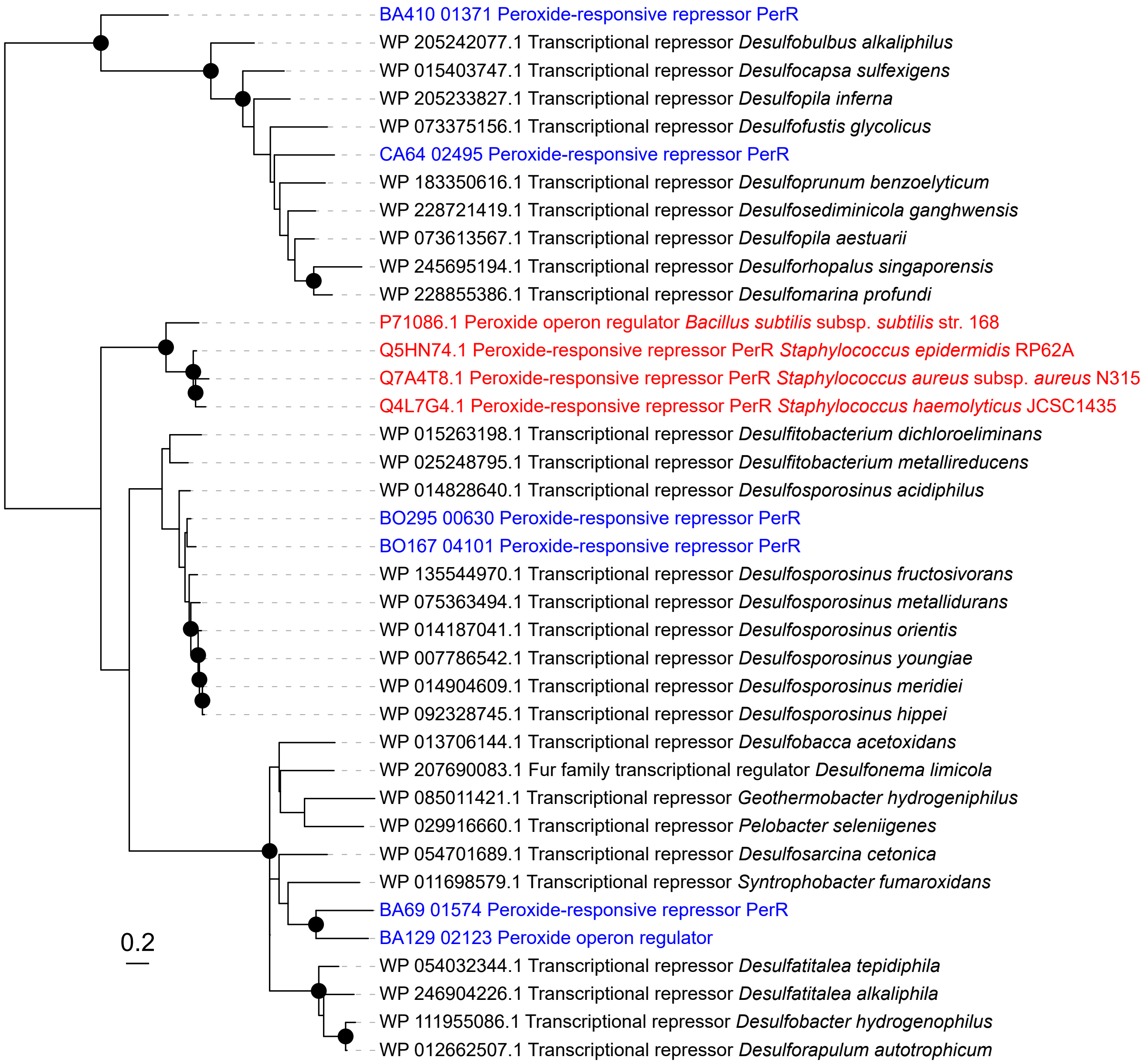
BA410 01371 Peroxide-responsive repressor PerR
WP 205242077.1 Transcriptional repressor *Desulfobulbus alkaliphilus*
WP 015403747.1 Transcriptional repressor *Desulfocapsa sulfexigens*
WP 205233827.1 Transcriptional repressor *Desulfopila inferna*
WP 073375156.1 Transcriptional repressor *Desulfofustis glycolicus*
CA64 02495 Peroxide-responsive repressor PerR
WP 183350616.1 Transcriptional repressor *Desulfoprunum benzoelyticum*
WP 228721419.1 Transcriptional repressor *Desulfosediminicola ganghwensis*
WP 073613567.1 Transcriptional repressor *Desulfopila aestuarii*
WP 245695194.1 Transcriptional repressor *Desulforhopalus singaporensis*
WP 228855386.1 Transcriptional repressor *Desulfomarina profundi*
P71086.1 Peroxide operon regulator *Bacillus subtilis* subsp. *subtilis* str. 168
Q5HN74.1 Peroxide-responsive repressor PerR *Staphylococcus epidermidis* RP62A
Q7A4T8.1 Peroxide-responsive repressor PerR *Staphylococcus aureus* subsp. *aureus* N315
Q4L7G4.1 Peroxide-responsive repressor PerR *Staphylococcus haemolyticus* JCSC1435
WP 015263198.1 Transcriptional repressor *Desulfitobacterium dichloroeliminans*
WP 025248795.1 Transcriptional repressor *Desulfitobacterium metallireducens*
WP 014828640.1 Transcriptional repressor *Desulfosporosinus acidiphilus*
BO295 00630 Peroxide-responsive repressor PerR
BO167 04101 Peroxide-responsive repressor PerR
WP 135544970.1 Transcriptional repressor *Desulfosporosinus fructosivorans*
WP 075363494.1 Transcriptional repressor *Desulfosporosinus metallidurans*
WP 014187041.1 Transcriptional repressor *Desulfosporosinus orientis*
WP 007786542.1 Transcriptional repressor *Desulfosporosinus youngiae*
WP 014904609.1 Transcriptional repressor *Desulfosporosinus meridiei*
WP 092328745.1 Transcriptional repressor *Desulfosporosinus hippei*
WP 013706144.1 Transcriptional repressor *Desulfobacca acetoxidans*
WP 207690083.1 Fur family transcriptional regulator *Desulfonema limicola*
WP 085011421.1 Transcriptional repressor *Geothermobacter hydrogeniphilus*
WP 029916660.1 Transcriptional repressor *Pelobacter seleniigenes*
WP 054701689.1 Transcriptional repressor *Desulfosarcina cetonica*
WP 011698579.1 Transcriptional repressor *Syntrophobacter fumaroxidans*
BA69 01574 Peroxide-responsive repressor PerR
BA129 02123 Peroxide operon regulator
WP 054032344.1 Transcriptional repressor *Desulfatitalea tepidiphila*
WP 246904226.1 Transcriptional repressor *Desulfatitalea alkaliphila*
WP 111955086.1 Transcriptional repressor *Desulfobacter hydrogenophilus*
WP 012662507.1 Transcriptional repressor *Desulforapulum autotrophicum*
0.2

Ahp

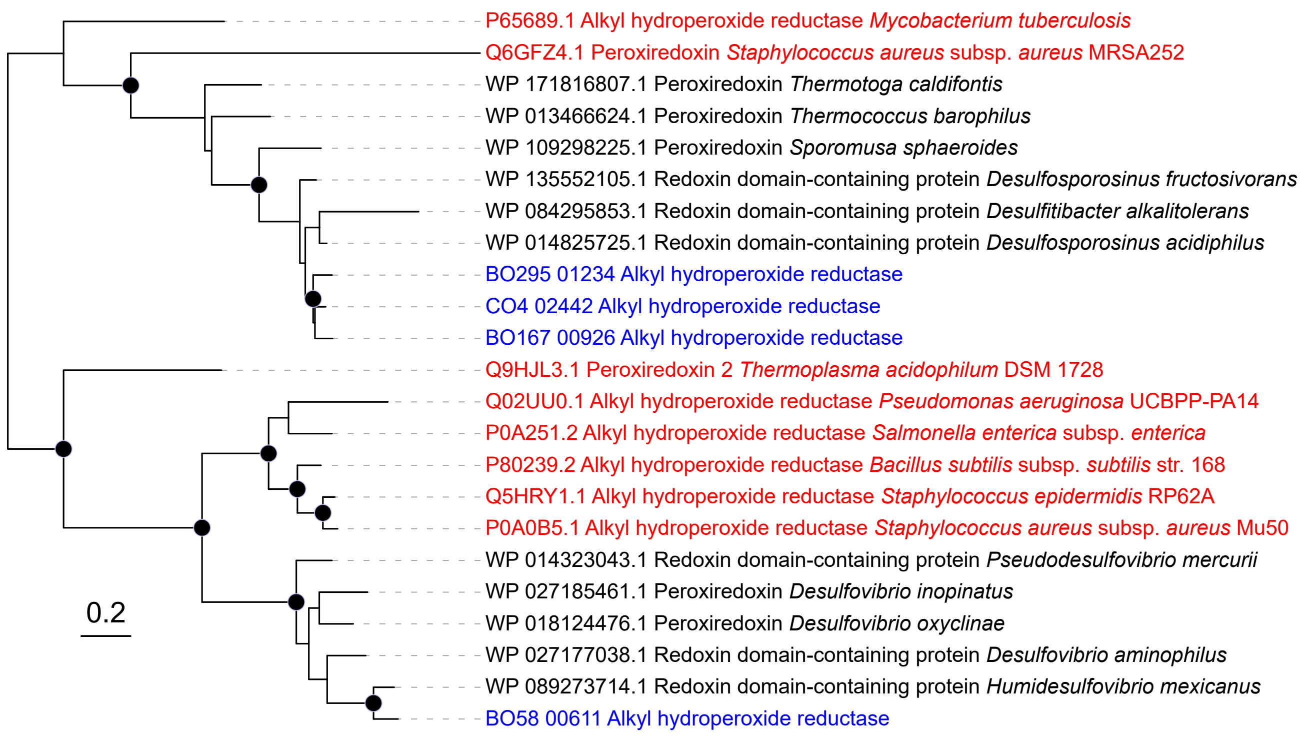
P65689.1 Alkyl hydroperoxide reductase Mycobacterium tuberculosis
Q6GFZ4.1 Peroxiredoxin Staphylococcus aureus subsp. aureus MRSA252
WP 171816807.1 Peroxiredoxin Thermotoga caldifontis
WP 013466624.1 Peroxiredoxin Thermococcus barophilus
WP 109298225.1 Peroxiredoxin Sporomusa sphaeroides
WP 135552105.1 Redoxin domain-containing protein Desulfosporosinus fructosivorans
WP 084295853.1 Redoxin domain-containing protein Desulfitibacter alkalitolerans
WP 014825725.1 Redoxin domain-containing protein Desulfosporosinus acidiphilus
BO295 01234 Alkyl hydroperoxide reductase
CO4 02442 Alkyl hydroperoxide reductase
BO167 00926 Alkyl hydroperoxide reductase
Q9HJL3.1 Peroxiredoxin 2 Thermoplasma acidophilum DSM 1728
Q02UU0.1 Alkyl hydroperoxide reductase Pseudomonas aeruginosa UCBPP-PA14
P0A251.2 Alkyl hydroperoxide reductase Salmonella enterica subsp. enterica
P80239.2 Alkyl hydroperoxide reductase Bacillus subtilis subsp. subtilis str. 168
Q5HRY1.1 Alkyl hydroperoxide reductase Staphylococcus epidermidis RP62A
P0A0B5.1 Alkyl hydroperoxide reductase Staphylococcus aureus subsp. aureus Mu50
WP 014323043.1 Redoxin domain-containing protein Pseudodesulfovibrio mercurii
WP 027185461.1 Peroxiredoxin Desulfovibrio inopinatus
WP 018124476.1 Peroxiredoxin Desulfovibrio oxyclinae
WP 027177038.1 Redoxin domain-containing protein Desulfovibrio aminophilus
WP 089273714.1 Redoxin domain-containing protein Humidesulfovibrio mexicanus
BO58 00611 Alkyl hydroperoxide reductase
0.2

Rub

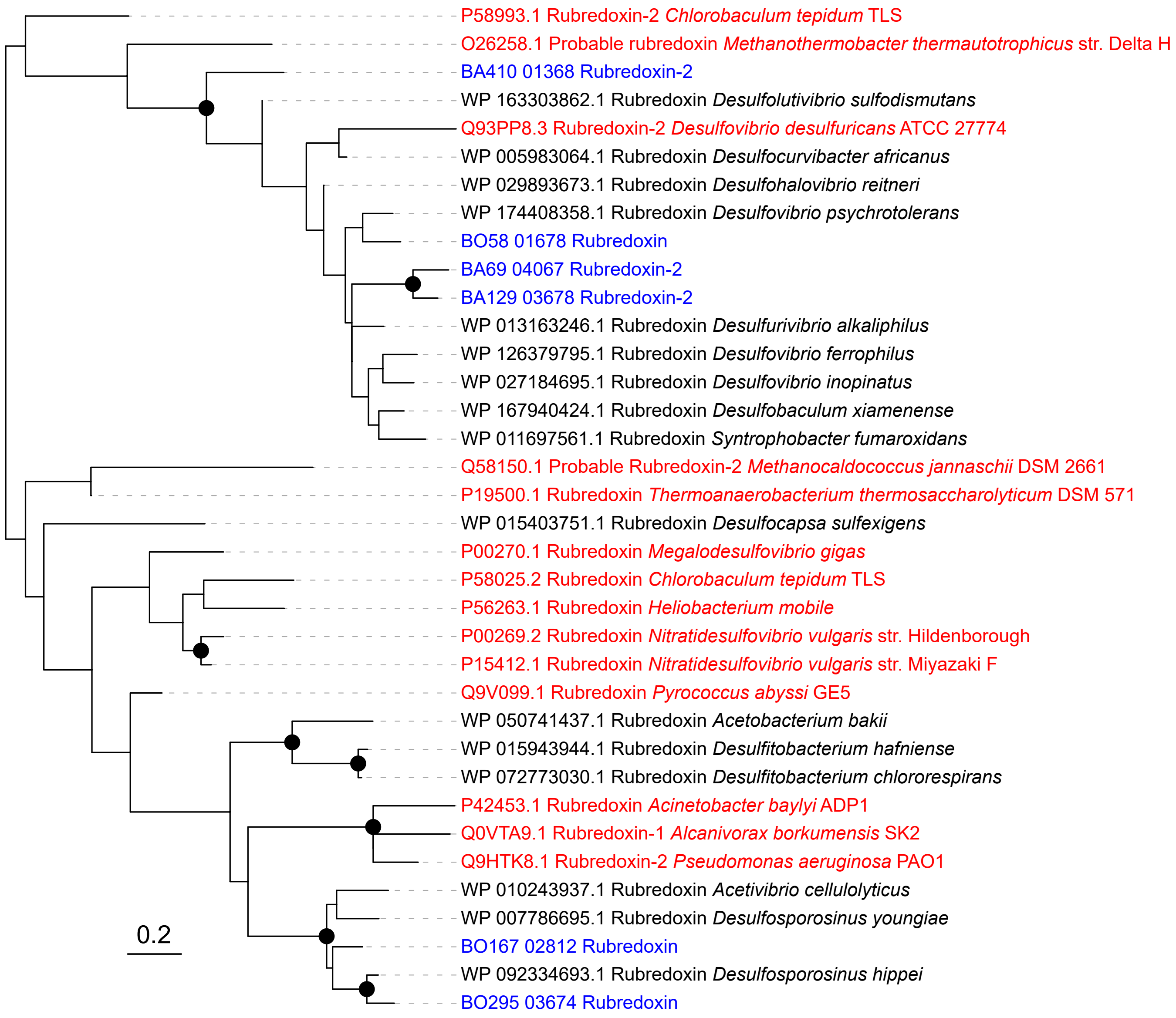
P58993.1 Rubredoxin-2 Chlorobaculum tepidum TLS
O26258.1 Probable rubredoxin Methanothermobacter thermautotrophicus str. Delta H
BA410 01368 Rubredoxin-2
WP 163303862.1 Rubredoxin Desulfolutivibrio sulfodismutans
Q93PP8.3 Rubredoxin-2 Desulfovibrio desulfuricans ATCC 27774
WP 005983064.1 Rubredoxin Desulfocurvibacter africanus
WP 029893673.1 Rubredoxin Desulfohalovibrio reitneri
WP 174408358.1 Rubredoxin Desulfovibrio psychrotolerans
BO58 01678 Rubredoxin
BA69 04067 Rubredoxin-2
BA129 03678 Rubredoxin-2
WP 013163246.1 Rubredoxin Desulfurivibrio alkaliphilus
WP 126379795.1 Rubredoxin Desulfovibrio ferrophilus
WP 027184695.1 Rubredoxin Desulfovibrio inopinatus
WP 167940424.1 Rubredoxin Desulfobaculum xiamenense
WP 011697561.1 Rubredoxin Syntrophobacter fumaroxidans
Q58150.1 Probable Rubredoxin-2 Methanocaldococcus jannaschii DSM 2661
P19500.1 Rubredoxin Thermoanaerobacterium thermosaccharolyticum DSM 571
WP 015403751.1 Rubredoxin Desulfocapsa sulfexigens
P00270.1 Rubredoxin Megalodesulfovibrio gigas
P58025.2 Rubredoxin Chlorobaculum tepidum TLS
P56263.1 Rubredoxin Heliobacterium mobile
P00269.2 Rubredoxin Nitratidesulfovibrio vulgaris str. Hildenborough
P15412.1 Rubredoxin Nitratidesulfovibrio vulgaris str. Miyazaki F
Q9V099.1 Rubredoxin Pyrococcus abyssi GE5
WP 050741437.1 Rubredoxin Acetobacterium bakii
WP 015943944.1 Rubredoxin Desulfitobacterium hafniense
WP 072773030.1 Rubredoxin Desulfitobacterium chlororespirans
P42453.1 Rubredoxin Acinetobacter baylyi ADP1
Q0VTA9.1 Rubredoxin-1 Alcanivorax borkumensis SK2
Q9HTK8.1 Rubredoxin-2 Pseudomonas aeruginosa PAO1
WP 010243937.1 Rubredoxin Acetivibrio cellulolyticus
WP 007786695.1 Rubredoxin Desulfosporosinus youngiae
BO167 02812 Rubredoxin
WP 092334693.1 Rubredoxin Desulfosporosinus hippei
BO295 03674 Rubredoxin
0.2

Rbr

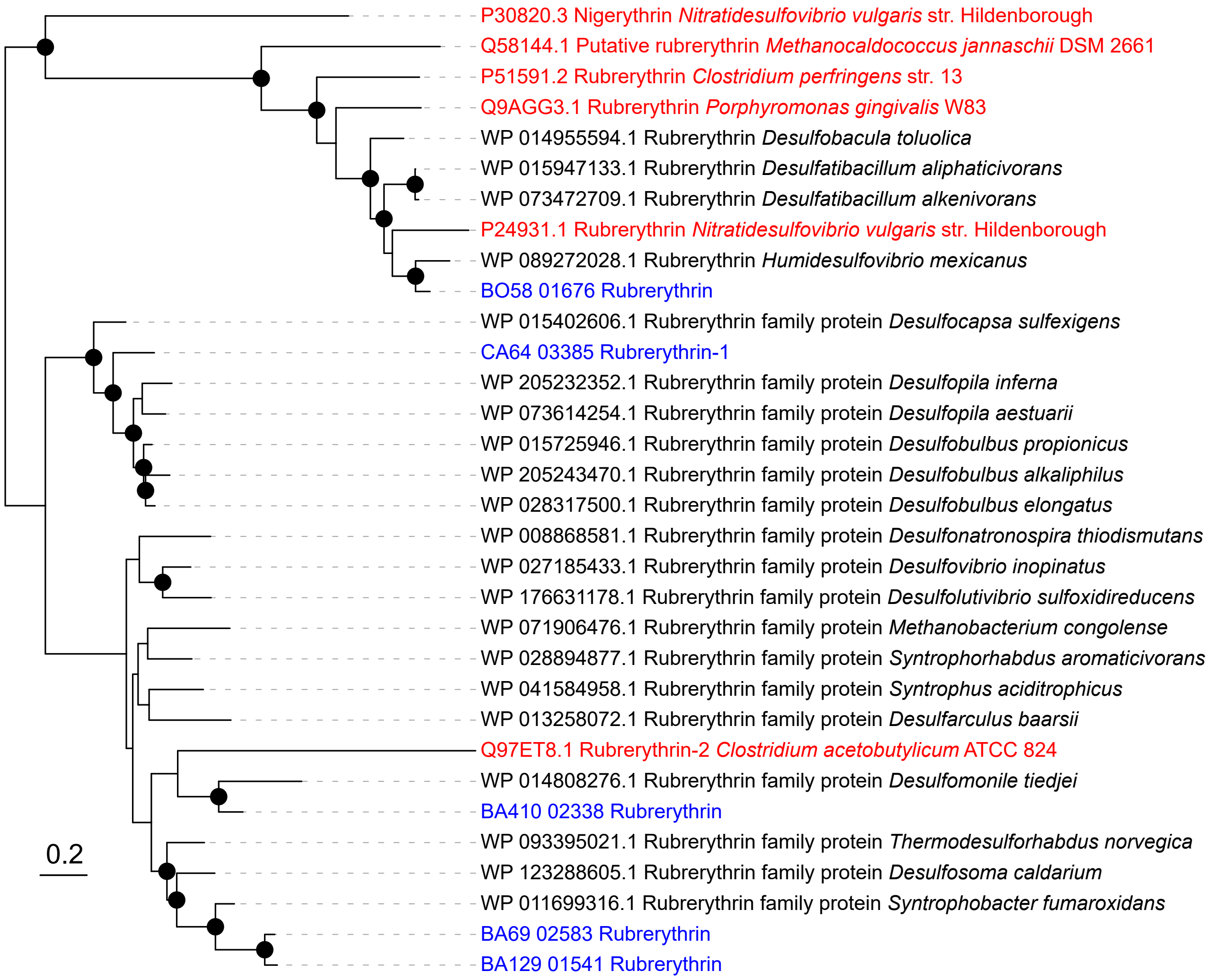
P30820.3 Nigerythrin Nitratidesulfovibrio vulgaris str. Hildenborough
Q58144.1 Putative rubrerythrin Methanocaldococcus jannaschii DSM 2661
P51591.2 Rubrerythrin Clostridium perfringens str. 13
Q9AGG3.1 Rubrerythrin Porphyromonas gingivalis W83
WP 014955594.1 Rubrerythrin Desulfobacula toluolica
WP 015947133.1 Rubrerythrin Desulfatibacillum aliphaticivorans
WP 073472709.1 Rubrerythrin Desulfatibacillum alkenivorans
P24931.1 Rubrerythrin Nitratidesulfovibrio vulgaris str. Hildenborough
WP 089272028.1 Rubrerythrin Humidesulfovibrio mexicanus
BO58 01676 Rubrerythrin
WP 015402606.1 Rubrerythrin family protein Desulfocapsa sulfexigens
CA64 03385 Rubrerythrin-1
WP 205232352.1 Rubrerythrin family protein Desulfopila inferna
WP 073614254.1 Rubrerythrin family protein Desulfopila aestuarii
WP 015725946.1 Rubrerythrin family protein Desulfobulbus propionicus
WP 205243470.1 Rubrerythrin family protein Desulfobulbus alkaliphilus
WP 028317500.1 Rubrerythrin family protein Desulfobulbus elongatus
WP 008868581.1 Rubrerythrin family protein Desulfonatronospira thiodismutans
WP 027185433.1 Rubrerythrin family protein Desulfovibrio inopinatus
WP 176631178.1 Rubrerythrin family protein Desulfolutivibrio sulfoxidireducens
WP 071906476.1 Rubrerythrin family protein Methanobacterium congolense
WP 028894877.1 Rubrerythrin family protein Syntrophorhabdus aromaticivorans
WP 041584958.1 Rubrerythrin family protein Syntrophus aciditrophicus
WP 013258072.1 Rubrerythrin family protein Desulfarculus baarsii
Q97ET8.1 Rubrerythrin-2 Clostridium acetobutylicum ATCC 824
WP 014808276.1 Rubrerythrin family protein Desulfomonile tiedjei
BA410 02338 Rubrerythrin
WP 093395021.1 Rubrerythrin family protein Thermodesulforhabdus norvegica
WP 123288605.1 Rubrerythrin family protein Desulfosoma caldarium
WP 011699316.1 Rubrerythrin family protein Syntrophobacter fumaroxidans
BA69 02583 Rubrerythrin
BA129 01541 Rubrerythrin
0.2

revRbr

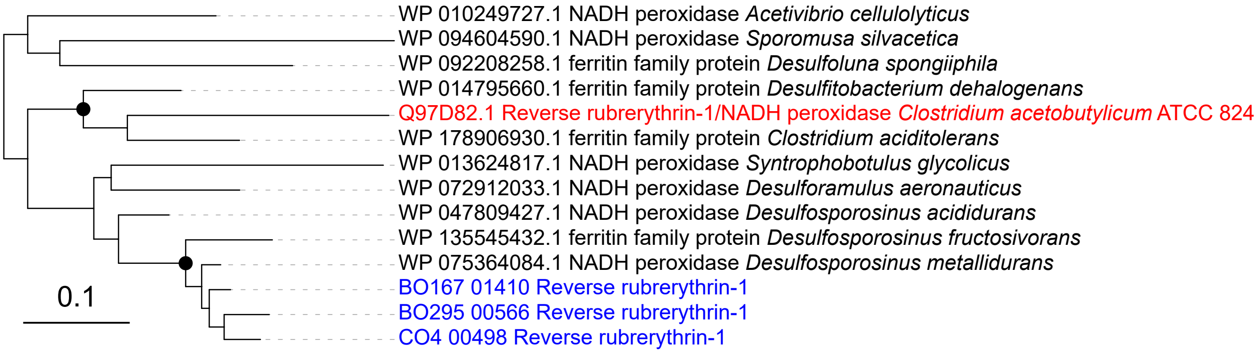
WP 010249727.1 NADH peroxidase Acetivibrio cellulolyticus
WP 094604590.1 NADH peroxidase Sporomusa silvacetica
WP 092208258.1 ferritin family protein Desulfoluna spongiiphila
WP 014795660.1 ferritin family protein Desulfitobacterium dehalogenans
Q97D82.1 Reverse rubrerythrin-1/NADH peroxidase Clostridium acetobutylicum ATCC 824
WP 178906930.1 ferritin family protein Clostridium aciditolerans
WP 013624817.1 NADH peroxidase Syntrophobotulus glycolicus
WP 072912033.1 NADH peroxidase Desulforamulus aeronauticus
WP 047809427.1 NADH peroxidase Desulfosporosinus acididurans
WP 135545432.1 ferritin family protein Desulfosporosinus fructosivorans
WP 075364084.1 NADH peroxidase Desulfosporosinus metallidurans
BO167 01410 Reverse rubrerythrin-1
BO295 00566 Reverse rubrerythrin-1
CO4 00498 Reverse rubrerythrin-1
0.1

Dfx

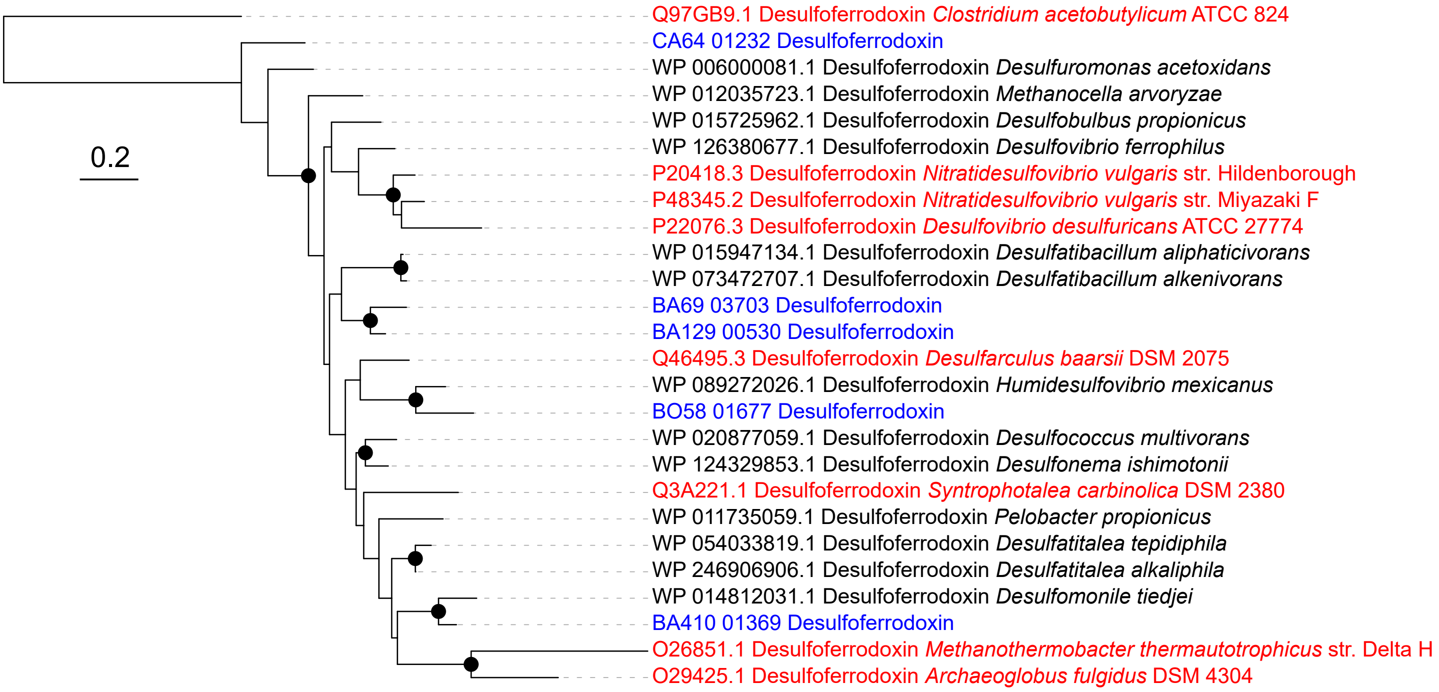

0.2
Q97GB9.1 Desulfoferrodoxin *Clostridium acetobutylicum* ATCC 824
CA64 01232 Desulfoferrodoxin
WP 006000081.1 Desulfoferrodoxin *Desulfuromonas acetoxidans*
WP 012035723.1 Desulfoferrodoxin *Methanocella arvoryzae*
WP 015725962.1 Desulfoferrodoxin *Desulfobulbus propionicus*
WP 126380677.1 Desulfoferrodoxin *Desulfovibrio ferrophilus*
P20418.3 Desulfoferrodoxin *Nitratidesulfovibrio vulgaris* str. Hildenborough
P48345.2 Desulfoferrodoxin *Nitratidesulfovibrio vulgaris* str. Miyazaki F
P22076.3 Desulfoferrodoxin *Desulfovibrio desulfuricans* ATCC 27774
WP 015947134.1 Desulfoferrodoxin *Desulfatibacillum aliphaticivorans*
WP 073472707.1 Desulfoferrodoxin *Desulfatibacillum alkenivorans*
BA69 03703 Desulfoferrodoxin
BA129 00530 Desulfoferrodoxin
Q46495.3 Desulfoferrodoxin *Desulfarculus baarsii* DSM 2075
WP 089272026.1 Desulfoferrodoxin *Humidesulfovibrio mexicanus*
BO58 01677 Desulfoferrodoxin
WP 020877059.1 Desulfoferrodoxin *Desulfococcus multivorans*
WP 124329853.1 Desulfoferrodoxin *Desulfonema ishimotonii*
Q3A221.1 Desulfoferrodoxin *Syntrophotalea carbinolica* DSM 2380
WP 011735059.1 Desulfoferrodoxin *Pelobacter propionicus*
WP 054033819.1 Desulfoferrodoxin *Desulfatitalea tepidiphila*
WP 246906906.1 Desulfoferrodoxin *Desulfatitalea alkaliphila*
WP 014812031.1 Desulfoferrodoxin *Desulfomonile tiedjei*
BA410 01369 Desulfoferrodoxin
O26851.1 Desulfoferrodoxin *Methanothermobacter thermautotrophicus* str. Delta H
O29425.1 Desulfoferrodoxin *Archaeoglobus fulgidus* DSM 4304

Tpx

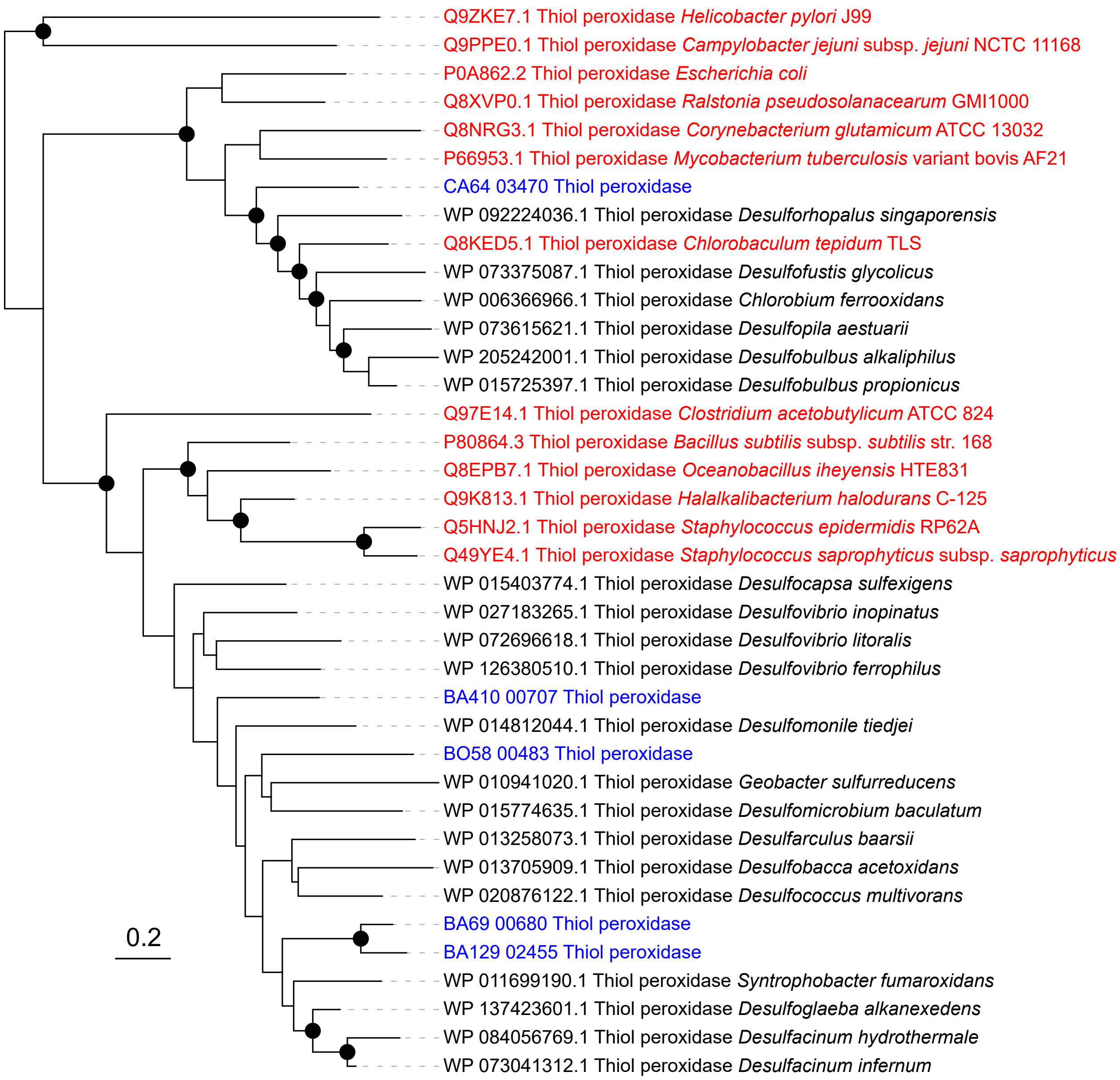

Q9ZKE7.1 Thiol peroxidase *Helicobacter pylori* J99
Q9PPE0.1 Thiol peroxidase *Campylobacter jejuni* subsp. *jejuni* NCTC 11168
P0A862.2 Thiol peroxidase *Escherichia coli*
Q8XVP0.1 Thiol peroxidase *Ralstonia pseudosolanacearum* GMI1000
Q8NRG3.1 Thiol peroxidase *Corynebacterium glutamicum* ATCC 13032
P66953.1 Thiol peroxidase *Mycobacterium tuberculosis* variant bovis AF21
CA64 03470 Thiol peroxidase
WP 092224036.1 Thiol peroxidase *Desulforhopalus singaporensis*
Q8KED5.1 Thiol peroxidase *Chlorobaculum tepidum* TLS
WP 073375087.1 Thiol peroxidase *Desulfofustis glycolicus*
WP 006366966.1 Thiol peroxidase *Chlorobium ferrooxidans*
WP 073615621.1 Thiol peroxidase *Desulfopila aestuarii*
WP 205242001.1 Thiol peroxidase *Desulfobulbus alkaliphilus*
WP 015725397.1 Thiol peroxidase *Desulfobulbus propionicus*
Q97E14.1 Thiol peroxidase *Clostridium acetobutylicum* ATCC 824
P80864.3 Thiol peroxidase *Bacillus subtilis* subsp. *subtilis* str. 168
Q8EPB7.1 Thiol peroxidase *Oceanobacillus iheyensis* HTE831
Q9K813.1 Thiol peroxidase *Halalkalibacterium halodurans* C-125
Q5HNJ2.1 Thiol peroxidase *Staphylococcus epidermidis* RP62A
Q49YE4.1 Thiol peroxidase *Staphylococcus saprophyticus* subsp. *saprophyticus*
WP 015403774.1 Thiol peroxidase *Desulfocapsa sulfexigens*
WP 027183265.1 Thiol peroxidase *Desulfovibrio inopinatus*
WP 072696618.1 Thiol peroxidase *Desulfovibrio litoralis*
WP 126380510.1 Thiol peroxidase *Desulfovibrio ferrophilus*
BA410 00707 Thiol peroxidase
WP 014812044.1 Thiol peroxidase *Desulfomonile tiedjei*
BO58 00483 Thiol peroxidase
WP 010941020.1 Thiol peroxidase *Geobacter sulfurreducens*
WP 015774635.1 Thiol peroxidase *Desulfomicrobium baculatum*
WP 013258073.1 Thiol peroxidase *Desulfarculus baarsii*
WP 013705909.1 Thiol peroxidase *Desulfobacca acetoxidans*
WP 020876122.1 Thiol peroxidase *Desulfococcus multivorans*
BA69 00680 Thiol peroxidase
BA129 02455 Thiol peroxidase
WP 011699190.1 Thiol peroxidase *Syntrophobacter fumaroxidans*
WP 137423601.1 Thiol peroxidase *Desulfoglaeba alkanexedens*
WP 084056769.1 Thiol peroxidase *Desulfacinum hydrothermale*
WP 073041312.1 Thiol peroxidase *Desulfacinum infernum*
0.2

TrxA

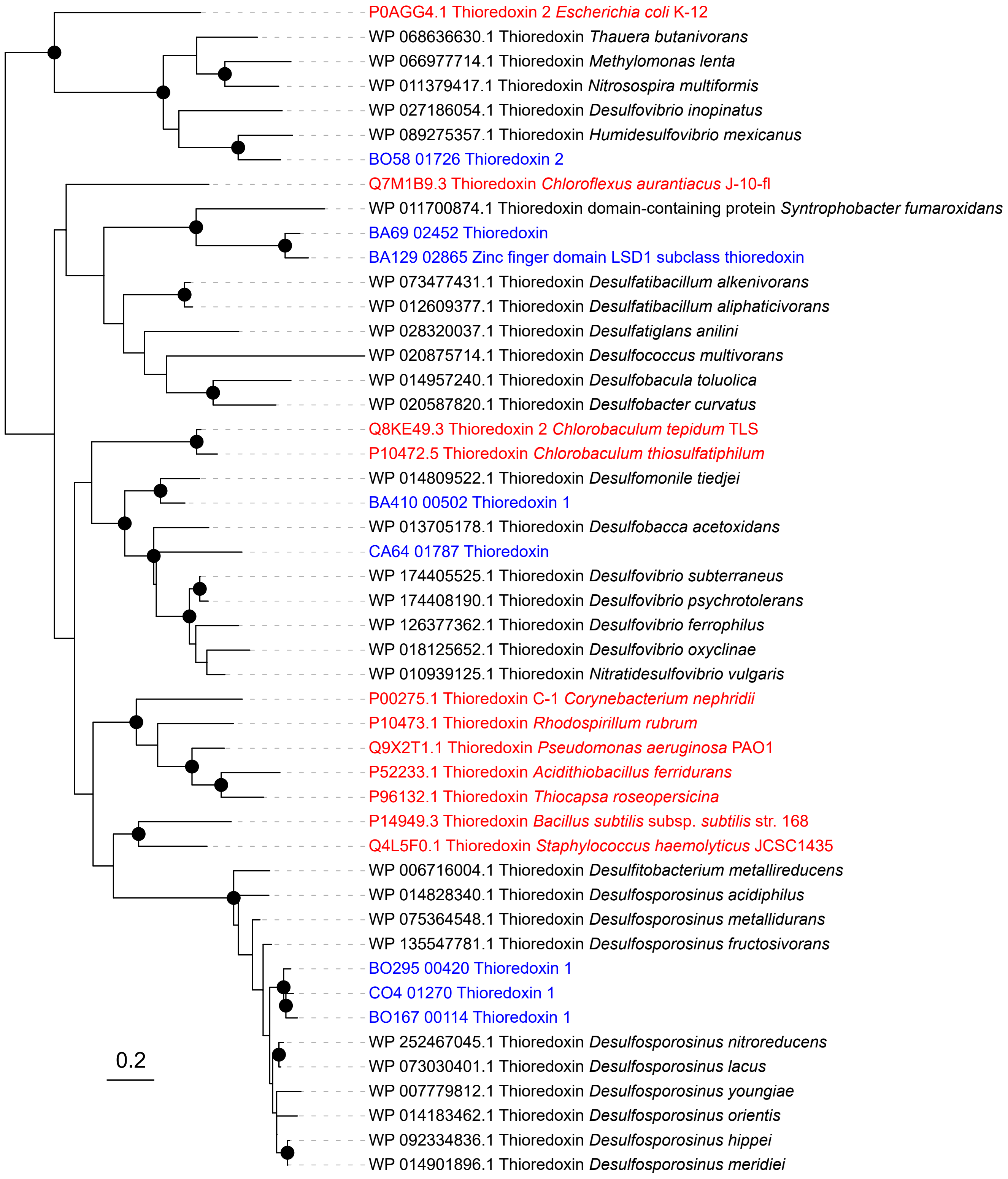
P0AGG4.1 Thioredoxin 2 Escherichia coli K-12
WP 068636630.1 Thioredoxin Thauera butanivorans
WP 066977714.1 Thioredoxin Methylomonas lenta
WP 011379417.1 Thioredoxin Nitrosospira multiformis
WP 027186054.1 Thioredoxin Desulfovibrio inopinatus
WP 089275357.1 Thioredoxin Humidesulfovibrio mexicanus
BO58 01726 Thioredoxin 2
Q7M1B9.3 Thioredoxin Chloroflexus aurantiacus J-10-fl
WP 011700874.1 Thioredoxin domain-containing protein Syntrophobacter fumaroxidans
BA69 02452 Thioredoxin
BA129 02865 Zinc finger domain LSD1 subclass thioredoxin
WP 073477431.1 Thioredoxin Desulfatibacillum alkenivorans
WP 012609377.1 Thioredoxin Desulfatibacillum aliphaticivorans
WP 028320037.1 Thioredoxin Desulfatiglans anilini
WP 020875714.1 Thioredoxin Desulfococcus multivorans
WP 014957240.1 Thioredoxin Desulfobacula toluolica
WP 020587820.1 Thioredoxin Desulfobacter curvatus
Q8KE49.3 Thioredoxin 2 Chlorobaculum tepidum TLS
P10472.5 Thioredoxin Chlorobaculum thiosulfatiphilum
WP 014809522.1 Thioredoxin Desulfomonile tiedjei
BA410 00502 Thioredoxin 1
WP 013705178.1 Thioredoxin Desulfobacca acetoxidans
CA64 01787 Thioredoxin
WP 174405525.1 Thioredoxin Desulfovibrio subterraneus
WP 174408190.1 Thioredoxin Desulfovibrio psychrotolerans
WP 126377362.1 Thioredoxin Desulfovibrio ferrophilus
WP 018125652.1 Thioredoxin Desulfovibrio oxyclinae
WP 010939125.1 Thioredoxin Nitratidesulfovibrio vulgaris
P00275.1 Thioredoxin C-1 Corynebacterium nephridii
P10473.1 Thioredoxin Rhodospirillum rubrum
Q9X2T1.1 Thioredoxin Pseudomonas aeruginosa PAO1
P52233.1 Thioredoxin Acidithiobacillus ferridurans
P96132.1 Thioredoxin Thiocapsa roseopersicina
P14949.3 Thioredoxin Bacillus subtilis subsp. subtilis str. 168
Q4L5F0.1 Thioredoxin Staphylococcus haemolyticus JCSC1435
WP 006716004.1 Thioredoxin Desulfitobacterium metallireducens
WP 014828340.1 Thioredoxin Desulfosporosinus acidiphilus
WP 075364548.1 Thioredoxin Desulfosporosinus metallidurans
WP 135547781.1 Thioredoxin Desulfosporosinus fructosivorans
BO295 00420 Thioredoxin 1
CO4 01270 Thioredoxin 1
BO167 00114 Thioredoxin 1
WP 252467045.1 Thioredoxin Desulfosporosinus nitroreducens
WP 073030401.1 Thioredoxin Desulfosporosinus lacus
WP 007779812.1 Thioredoxin Desulfosporosinus youngiae
WP 014183462.1 Thioredoxin Desulfosporosinus orientis
WP 092334836.1 Thioredoxin Desulfosporosinus hippei
WP 014901896.1 Thioredoxin Desulfosporosinus meridiei
0.2

TrxB

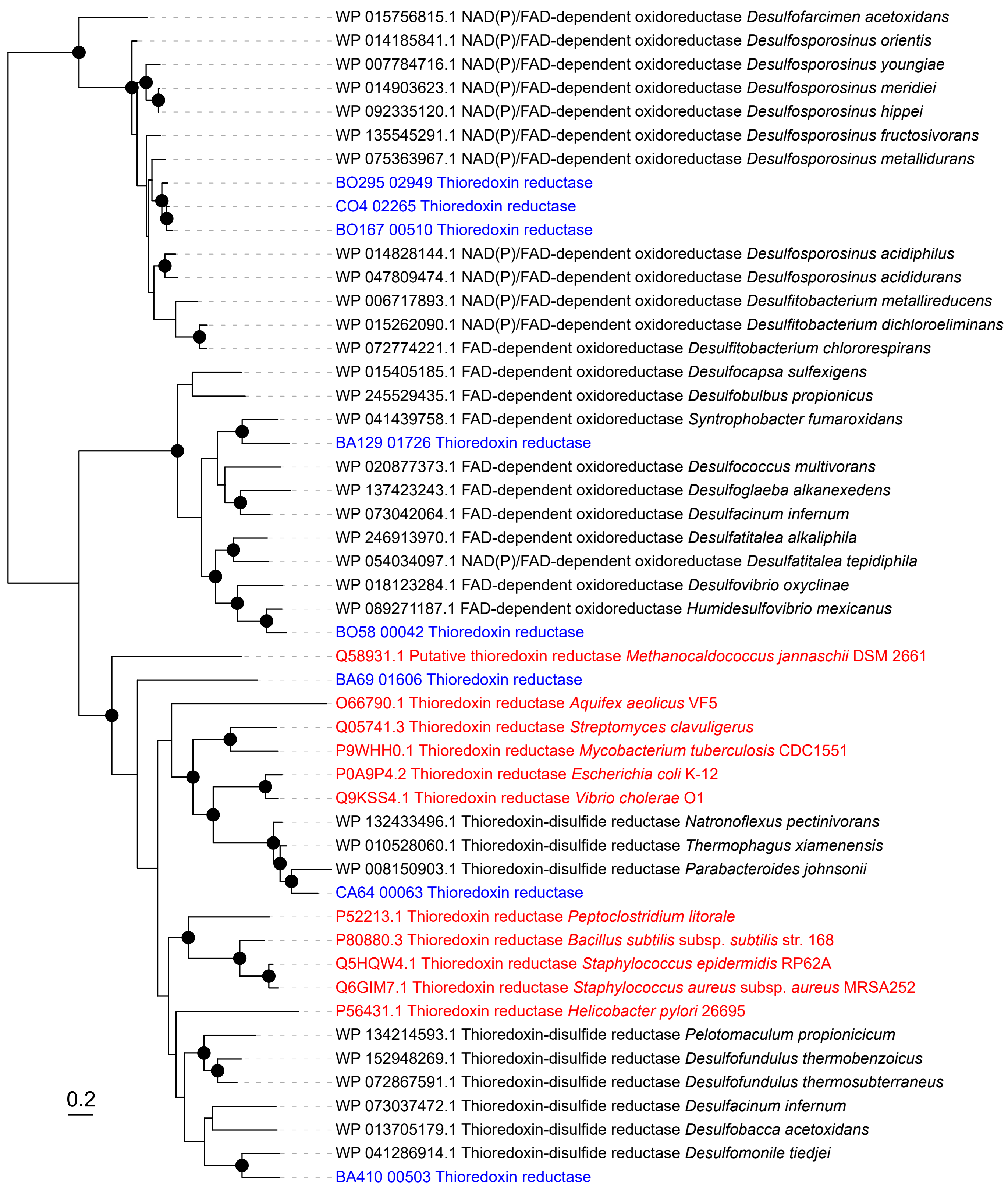

WP 015756815.1 NAD(P)/FAD-dependent oxidoreductase Desulfofarcimen acetoxidans
WP 014185841.1 NAD(P)/FAD-dependent oxidoreductase Desulfosporosinus orientis
WP 007784716.1 NAD(P)/FAD-dependent oxidoreductase Desulfosporosinus youngiae
WP 014903623.1 NAD(P)/FAD-dependent oxidoreductase Desulfosporosinus meridiei
WP 092335120.1 NAD(P)/FAD-dependent oxidoreductase Desulfosporosinus hippei
WP 135545291.1 NAD(P)/FAD-dependent oxidoreductase Desulfosporosinus fructosivorans
WP 075363967.1 NAD(P)/FAD-dependent oxidoreductase Desulfosporosinus metallidurans
BO295 02949 Thioredoxin reductase
CO4 02265 Thioredoxin reductase
BO167 00510 Thioredoxin reductase
WP 014828144.1 NAD(P)/FAD-dependent oxidoreductase Desulfosporosinus acidiphilus
WP 047809474.1 NAD(P)/FAD-dependent oxidoreductase Desulfosporosinus acididurans
WP 006717893.1 NAD(P)/FAD-dependent oxidoreductase Desulfitobacterium metallireducens
WP 015262090.1 NAD(P)/FAD-dependent oxidoreductase Desulfitobacterium dichloroeliminans
WP 072774221.1 FAD-dependent oxidoreductase Desulfitobacterium chlororespirans
WP 015405185.1 FAD-dependent oxidoreductase Desulfocapsa sulfexigens
WP 245529435.1 FAD-dependent oxidoreductase Desulfobulbus propionicus
WP 041439758.1 FAD-dependent oxidoreductase Syntrophobacter fumaroxidans
BA129 01726 Thioredoxin reductase
WP 020877373.1 FAD-dependent oxidoreductase Desulfococcus multivorans
WP 137423243.1 FAD-dependent oxidoreductase Desulfoglaeba alkanexedens
WP 073042064.1 FAD-dependent oxidoreductase Desulfacinum infernum
WP 246913970.1 FAD-dependent oxidoreductase Desulfatitalea alkaliphila
WP 054034097.1 NAD(P)/FAD-dependent oxidoreductase Desulfatitalea tepidiphila
WP 018123284.1 FAD-dependent oxidoreductase Desulfovibrio oxyclinae
WP 089271187.1 FAD-dependent oxidoreductase Humidesulfovibrio mexicanus
BO58 00042 Thioredoxin reductase
Q58931.1 Putative thioredoxin reductase Methanocaldococcus jannaschii DSM 2661
BA69 01606 Thioredoxin reductase
O66790.1 Thioredoxin reductase Aquifex aeolicus VF5
Q05741.3 Thioredoxin reductase Streptomyces clavuligerus
P9WHH0.1 Thioredoxin reductase Mycobacterium tuberculosis CDC1551
P0A9P4.2 Thioredoxin reductase Escherichia coli K-12
Q9KSS4.1 Thioredoxin reductase Vibrio cholerae O1
WP 132433496.1 Thioredoxin-disulfide reductase Natronoflexus pectinivorans
WP 010528060.1 Thioredoxin-disulfide reductase Thermophagus xiamenensis
WP 008150903.1 Thioredoxin-disulfide reductase Parabacteroides johnsonii
CA64 00063 Thioredoxin reductase
P52213.1 Thioredoxin reductase Peptoclostridium litorale
P80880.3 Thioredoxin reductase Bacillus subtilis subsp. subtilis str. 168
Q5HQW4.1 Thioredoxin reductase Staphylococcus epidermidis RP62A
Q6GIM7.1 Thioredoxin reductase Staphylococcus aureus subsp. aureus MRSA252
P56431.1 Thioredoxin reductase Helicobacter pylori 26695
WP 134214593.1 Thioredoxin-disulfide reductase Pelotomaculum propionicicum
WP 152948269.1 Thioredoxin-disulfide reductase Desulfofundulus thermobenzoicus
WP 072867591.1 Thioredoxin-disulfide reductase Desulfofundulus thermosubterraneus
WP 073037472.1 Thioredoxin-disulfide reductase Desulfacinum infernum
WP 013705179.1 Thioredoxin-disulfide reductase Desulfobacca acetoxidans
WP 041286914.1 Thioredoxin-disulfide reductase Desulfomonile tiedjei
BA410 00503 Thioredoxin reductase
0.2

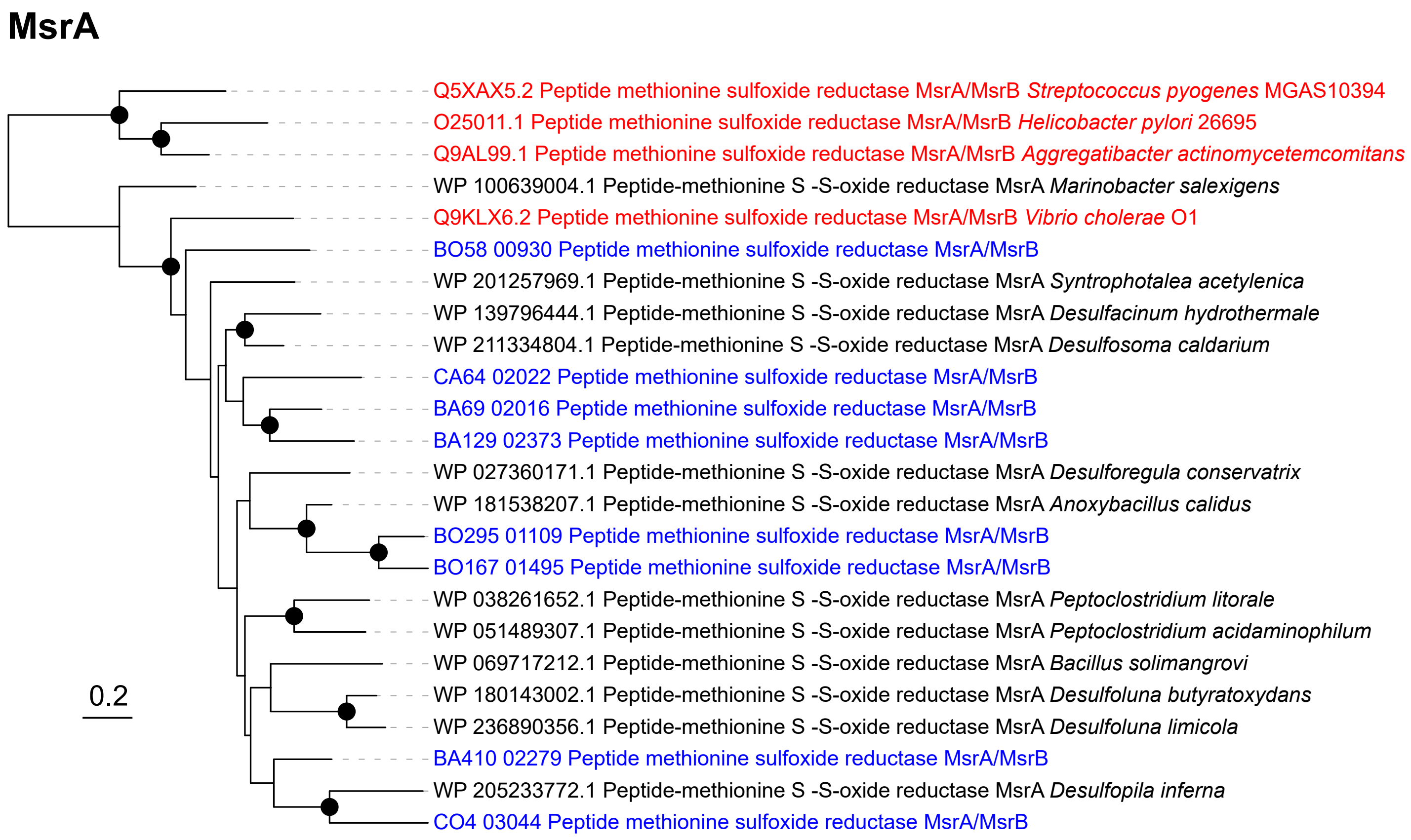
MsrA
Q5XAX5.2 Peptide methionine sulfoxide reductase MsrA/MsrB *Streptococcus pyogenes* MGAS10394
O25011.1 Peptide methionine sulfoxide reductase MsrA/MsrB *Helicobacter pylori* 26695
Q9AL99.1 Peptide methionine sulfoxide reductase MsrA/MsrB *Aggregatibacter actinomycetemcomitans*
WP 100639004.1 Peptide-methionine S -S-oxide reductase MsrA *Marinobacter salexigens*
Q9KLX6.2 Peptide methionine sulfoxide reductase MsrA/MsrB *Vibrio cholerae* O1
BO58 00930 Peptide methionine sulfoxide reductase MsrA/MsrB
WP 201257969.1 Peptide-methionine S -S-oxide reductase MsrA *Syntrophotalea acetylenica*
WP 139796444.1 Peptide-methionine S -S-oxide reductase MsrA *Desulfacinum hydrothermale*
WP 211334804.1 Peptide-methionine S -S-oxide reductase MsrA *Desulfosoma caldarium*
CA64 02022 Peptide methionine sulfoxide reductase MsrA/MsrB
BA69 02016 Peptide methionine sulfoxide reductase MsrA/MsrB
BA129 02373 Peptide methionine sulfoxide reductase MsrA/MsrB
WP 027360171.1 Peptide-methionine S -S-oxide reductase MsrA *Desulforegula conservatrix*
WP 181538207.1 Peptide-methionine S -S-oxide reductase MsrA *Anoxybacillus calidus*
BO295 01109 Peptide methionine sulfoxide reductase MsrA/MsrB
BO167 01495 Peptide methionine sulfoxide reductase MsrA/MsrB
WP 038261652.1 Peptide-methionine S -S-oxide reductase MsrA *Peptoclostridium litorale*
WP 051489307.1 Peptide-methionine S -S-oxide reductase MsrA *Peptoclostridium acidaminophilum*
WP 069717212.1 Peptide-methionine S -S-oxide reductase MsrA *Bacillus solimangrovi*
WP 180143002.1 Peptide-methionine S -S-oxide reductase MsrA *Desulfoluna butyratoxydans*
WP 236890356.1 Peptide-methionine S -S-oxide reductase MsrA *Desulfoluna limicola*
BA410 02279 Peptide methionine sulfoxide reductase MsrA/MsrB
WP 205233772.1 Peptide-methionine S -S-oxide reductase MsrA *Desulfopila inferna*
CO4 03044 Peptide methionine sulfoxide reductase MsrA/MsrB
0.2

ClpB

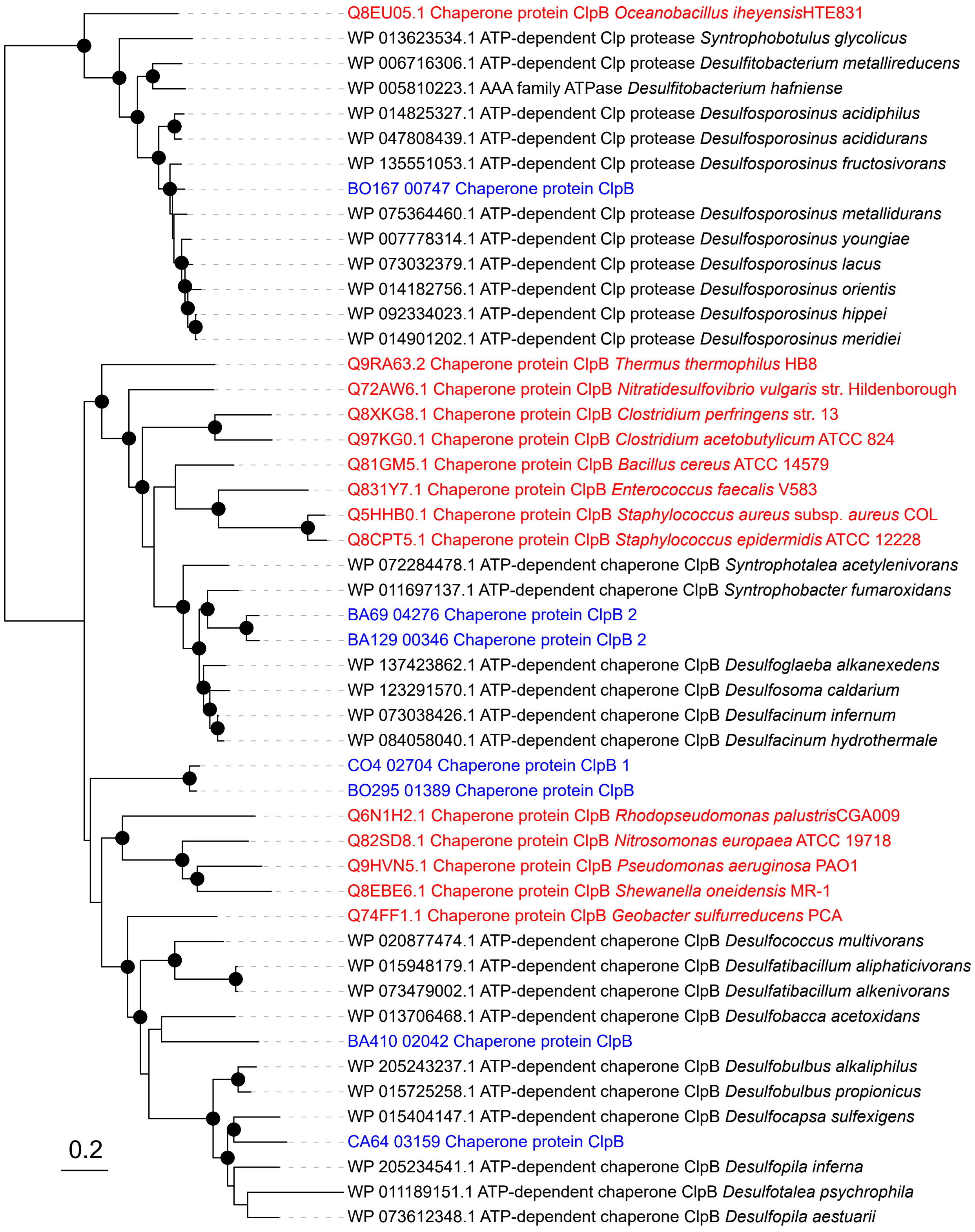
Q8EU05.1 Chaperone protein ClpB Oceanobacillus iheyensisHTE831
WP 013623534.1 ATP-dependent Clp protease Syntrophobotulus glycolicus
WP 006716306.1 ATP-dependent Clp protease Desulfitobacterium metallireducens
WP 005810223.1 AAA family ATPase Desulfitobacterium hafniense
WP 014825327.1 ATP-dependent Clp protease Desulfosporosinus acidiphilus
WP 047808439.1 ATP-dependent Clp protease Desulfosporosinus acididurans
WP 135551053.1 ATP-dependent Clp protease Desulfosporosinus fructosivorans
BO167 00747 Chaperone protein ClpB
WP 075364460.1 ATP-dependent Clp protease Desulfosporosinus metallidurans
WP 007778314.1 ATP-dependent Clp protease Desulfosporosinus youngiae
WP 073032379.1 ATP-dependent Clp protease Desulfosporosinus lacus
WP 014182756.1 ATP-dependent Clp protease Desulfosporosinus orientis
WP 092334023.1 ATP-dependent Clp protease Desulfosporosinus hippei
WP 014901202.1 ATP-dependent Clp protease Desulfosporosinus meridiei
Q9RA63.2 Chaperone protein ClpB Thermus thermophilus HB8
Q72AW6.1 Chaperone protein ClpB Nitratidesulfovibrio vulgaris str. Hildenborough
Q8XKG8.1 Chaperone protein ClpB Clostridium perfringens str. 13
Q97KG0.1 Chaperone protein ClpB Clostridium acetobutylicum ATCC 824
Q81GM5.1 Chaperone protein ClpB Bacillus cereus ATCC 14579
Q831Y7.1 Chaperone protein ClpB Enterococcus faecalis V583
Q5HHB0.1 Chaperone protein ClpB Staphylococcus aureus subsp. aureus COL
Q8CPT5.1 Chaperone protein ClpB Staphylococcus epidermidis ATCC 12228
WP 072284478.1 ATP-dependent chaperone ClpB Syntrophotalea acetylenivorans
WP 011697137.1 ATP-dependent chaperone ClpB Syntrophobacter fumaroxidans
BA69 04276 Chaperone protein ClpB 2
BA129 00346 Chaperone protein ClpB 2
WP 137423862.1 ATP-dependent chaperone ClpB Desulfoglaeba alkanexedens
WP 123291570.1 ATP-dependent chaperone ClpB Desulfosoma caldarium
WP 073038426.1 ATP-dependent chaperone ClpB Desulfacinum infernum
WP 084058040.1 ATP-dependent chaperone ClpB Desulfacinum hydrothermale
CO4 02704 Chaperone protein ClpB 1
BO295 01389 Chaperone protein ClpB
Q6N1H2.1 Chaperone protein ClpB Rhodopseudomonas palustrisCGA009
Q82SD8.1 Chaperone protein ClpB Nitrosomonas europaea ATCC 19718
Q9HVN5.1 Chaperone protein ClpB Pseudomonas aeruginosa PAO1
Q8EBE6.1 Chaperone protein ClpB Shewanella oneidensis MR-1
Q74FF1.1 Chaperone protein ClpB Geobacter sulfurreducens PCA
WP 020877474.1 ATP-dependent chaperone ClpB Desulfococcus multivorans
WP 015948179.1 ATP-dependent chaperone ClpB Desulfatibacillum aliphaticivorans
WP 073479002.1 ATP-dependent chaperone ClpB Desulfatibacillum alkenivorans
WP 013706468.1 ATP-dependent chaperone ClpB Desulfobacca acetoxidans
BA410 02042 Chaperone protein ClpB
WP 205243237.1 ATP-dependent chaperone ClpB Desulfobulbus alkaliphilus
WP 015725258.1 ATP-dependent chaperone ClpB Desulfobulbus propionicus
WP 015404147.1 ATP-dependent chaperone ClpB Desulfocapsa sulfexigens
CA64 03159 Chaperone protein ClpB
WP 205234541.1 ATP-dependent chaperone ClpB Desulfopila inferna
WP 011189151.1 ATP-dependent chaperone ClpB Desulfotalea psychrophila
WP 073612348.1 ATP-dependent chaperone ClpB Desulfopila aestuarii
0.2

DnaK

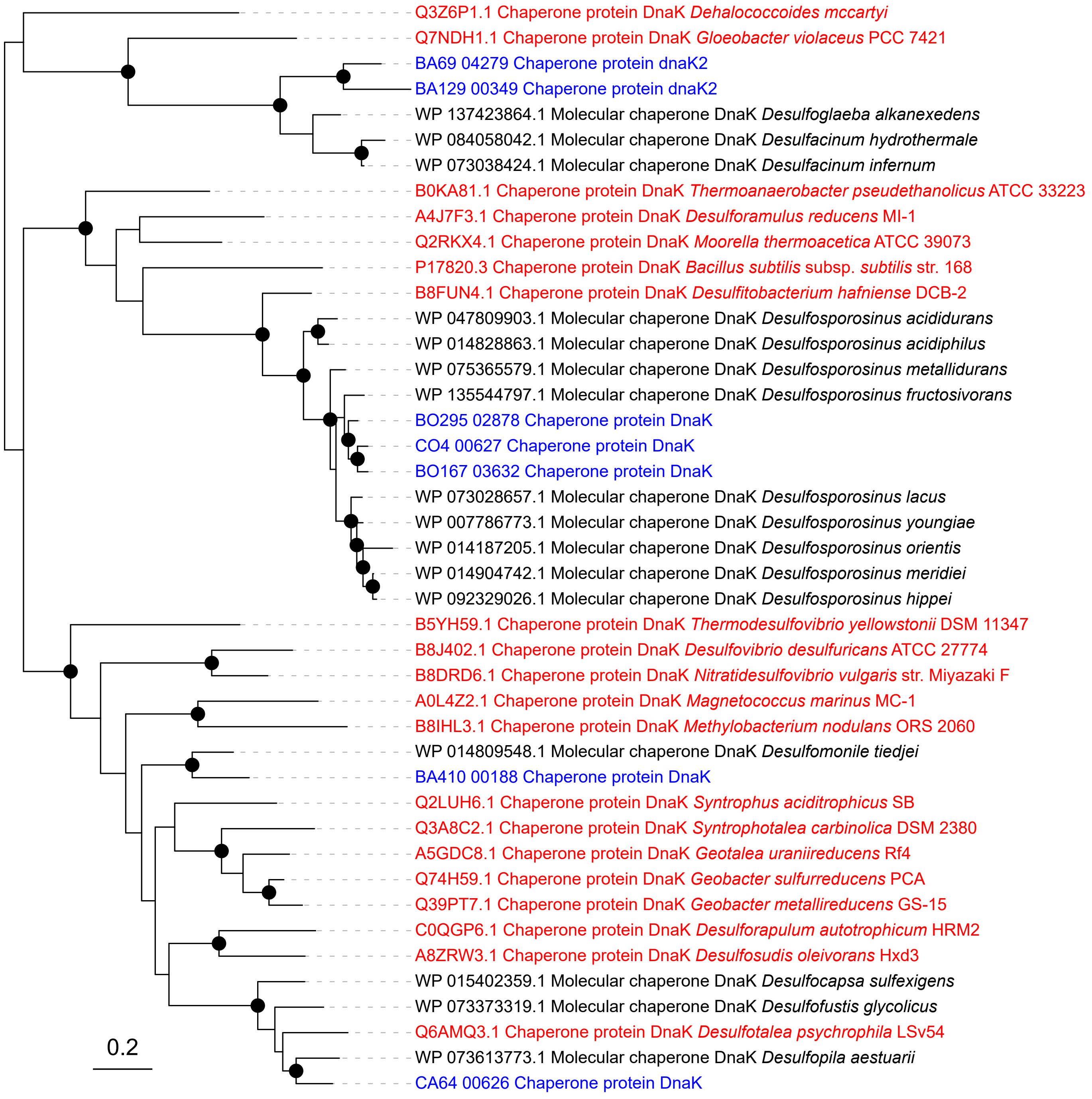

Q3Z6P1.1 Chaperone protein DnaK *Dehalococcoides mccartyi*
Q7NDH1.1 Chaperone protein DnaK *Gloeobacter violaceus* PCC 7421
BA69 04279 Chaperone protein dnaK2
BA129 00349 Chaperone protein dnaK2
WP 137423864.1 Molecular chaperone DnaK *Desulfoglaeba alkanexedens*
WP 084058042.1 Molecular chaperone DnaK *Desulfacinum hydrothermale*
WP 073038424.1 Molecular chaperone DnaK *Desulfacinum infernum*
B0KA81.1 Chaperone protein DnaK *Thermoanaerobacter pseudethanolicus* ATCC 33223
A4J7F3.1 Chaperone protein DnaK *Desulforamulus reducens* MI-1
Q2RKX4.1 Chaperone protein DnaK *Moorella thermoacetica* ATCC 39073
P17820.3 Chaperone protein DnaK *Bacillus subtilis* subsp. *subtilis* str. 168
B8FUN4.1 Chaperone protein DnaK *Desulfitobacterium hafniense* DCB-2
WP 047809903.1 Molecular chaperone DnaK *Desulfosporosinus acididurans*
WP 014828863.1 Molecular chaperone DnaK *Desulfosporosinus acidiphilus*
WP 075365579.1 Molecular chaperone DnaK *Desulfosporosinus metallidurans*
WP 135544797.1 Molecular chaperone DnaK *Desulfosporosinus fructosivorans*
BO295 02878 Chaperone protein DnaK
CO4 00627 Chaperone protein DnaK
BO167 03632 Chaperone protein DnaK
WP 073028657.1 Molecular chaperone DnaK *Desulfosporosinus lacus*
WP 007786773.1 Molecular chaperone DnaK *Desulfosporosinus youngiae*
WP 014187205.1 Molecular chaperone DnaK *Desulfosporosinus orientis*
WP 014904742.1 Molecular chaperone DnaK *Desulfosporosinus meridiei*
WP 092329026.1 Molecular chaperone DnaK *Desulfosporosinus hippei*
B5YH59.1 Chaperone protein DnaK *Thermodesulfovibrio yellowstonii* DSM 11347
B8J402.1 Chaperone protein DnaK *Desulfovibrio desulfuricans* ATCC 27774
B8DRD6.1 Chaperone protein DnaK *Nitratidesulfovibrio vulgaris* str. Miyazaki F
A0L4Z2.1 Chaperone protein DnaK *Magnetococcus marinus* MC-1
B8IHL3.1 Chaperone protein DnaK *Methylobacterium nodulans* ORS 2060
WP 014809548.1 Molecular chaperone DnaK *Desulfomonile tiedjei*
BA410 00188 Chaperone protein DnaK
Q2LUH6.1 Chaperone protein DnaK *Syntrophus aciditrophicus* SB
Q3A8C2.1 Chaperone protein DnaK *Syntrophotalea carbinolica* DSM 2380
A5GDC8.1 Chaperone protein DnaK *Geotalea uraniireducens* Rf4
Q74H59.1 Chaperone protein DnaK *Geobacter sulfurreducens* PCA
Q39PT7.1 Chaperone protein DnaK *Geobacter metallireducens* GS-15
C0QGP6.1 Chaperone protein DnaK *Desulforapulum autotrophicum* HRM2
A8ZRW3.1 Chaperone protein DnaK *Desulfosudis oleivorans* Hxd3
WP 015402359.1 Molecular chaperone DnaK *Desulfocapsa sulfexigens*
WP 073373319.1 Molecular chaperone DnaK *Desulfofustis glycolicus*
Q6AMQ3.1 Chaperone protein DnaK *Desulfotalea psychrophila* LSv54
WP 073613773.1 Molecular chaperone DnaK *Desulfopila aestuarii*
CA64 00626 Chaperone protein DnaK
0.2

DnaJ

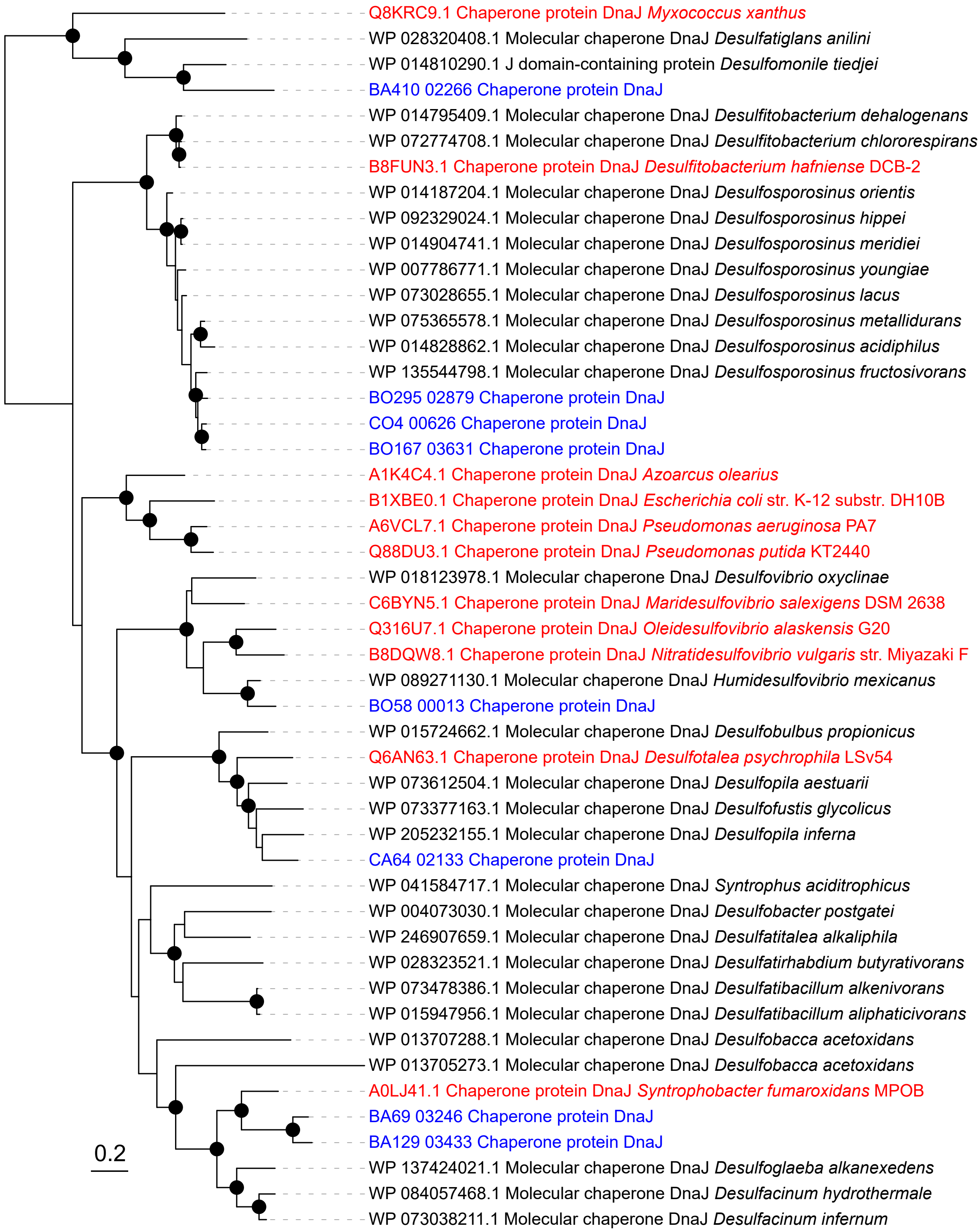

Q8KRC9.1 Chaperone protein DnaJ *Myxococcus xanthus*
WP 028320408.1 Molecular chaperone DnaJ *Desulfatiglans anilini*
WP 014810290.1 J domain-containing protein *Desulfomonile tiedjei*
BA410 02266 Chaperone protein DnaJ
WP 014795409.1 Molecular chaperone DnaJ *Desulfitobacterium dehalogenans*
WP 072774708.1 Molecular chaperone DnaJ *Desulfitobacterium chlororespirans*
B8FUN3.1 Chaperone protein DnaJ *Desulfitobacterium hafniense* DCB-2
WP 014187204.1 Molecular chaperone DnaJ *Desulfosporosinus orientis*
WP 092329024.1 Molecular chaperone DnaJ *Desulfosporosinus hippei*
WP 014904741.1 Molecular chaperone DnaJ *Desulfosporosinus meridiei*
WP 007786771.1 Molecular chaperone DnaJ *Desulfosporosinus youngiae*
WP 073028655.1 Molecular chaperone DnaJ *Desulfosporosinus lacus*
WP 075365578.1 Molecular chaperone DnaJ *Desulfosporosinus metallidurans*
WP 014828862.1 Molecular chaperone DnaJ *Desulfosporosinus acidiphilus*
WP 135544798.1 Molecular chaperone DnaJ *Desulfosporosinus fructosivorans*
BO295 02879 Chaperone protein DnaJ
CO4 00626 Chaperone protein DnaJ
BO167 03631 Chaperone protein DnaJ
A1K4C4.1 Chaperone protein DnaJ *Azoarcus olearius*
B1XBE0.1 Chaperone protein DnaJ *Escherichia coli* str. K-12 substr. DH10B
A6VCL7.1 Chaperone protein DnaJ *Pseudomonas aeruginosa* PA7
Q88DU3.1 Chaperone protein DnaJ *Pseudomonas putida* KT2440
WP 018123978.1 Molecular chaperone DnaJ *Desulfovibrio oxyclinae*
C6BYN5.1 Chaperone protein DnaJ *Maridesulfovibrio salexigens* DSM 2638
Q316U7.1 Chaperone protein DnaJ *Oleidesulfovibrio alaskensis* G20
B8DQW8.1 Chaperone protein DnaJ *Nitratidesulfovibrio vulgaris* str. Miyazaki F
WP 089271130.1 Molecular chaperone DnaJ *Humidesulfovibrio mexicanus*
BO58 00013 Chaperone protein DnaJ
WP 015724662.1 Molecular chaperone DnaJ *Desulfobulbus propionicus*
Q6AN63.1 Chaperone protein DnaJ *Desulfotalea psychrophila* LSv54
WP 073612504.1 Molecular chaperone DnaJ *Desulfopila aestuarii*
WP 073377163.1 Molecular chaperone DnaJ *Desulfofustis glycolicus*
WP 205232155.1 Molecular chaperone DnaJ *Desulfopila inferna*
CA64 02133 Chaperone protein DnaJ
WP 041584717.1 Molecular chaperone DnaJ *Syntrophus aciditrophicus*
WP 004073030.1 Molecular chaperone DnaJ *Desulfobacter postgatei*
WP 246907659.1 Molecular chaperone DnaJ *Desulfatitalea alkaliphila*
WP 028323521.1 Molecular chaperone DnaJ *Desulfatirhabdium butyrativorans*
WP 073478386.1 Molecular chaperone DnaJ *Desulfatibacillum alkenivorans*
WP 015947956.1 Molecular chaperone DnaJ *Desulfatibacillum aliphaticivorans*
WP 013707288.1 Molecular chaperone DnaJ *Desulfobacca acetoxidans*
WP 013705273.1 Molecular chaperone DnaJ *Desulfobacca acetoxidans*
A0LJ41.1 Chaperone protein DnaJ *Syntrophobacter fumaroxidans* MPOB
BA69 03246 Chaperone protein DnaJ
BA129 03433 Chaperone protein DnaJ
WP 137424021.1 Molecular chaperone DnaJ *Desulfoglaeba alkanexedens*
WP 084057468.1 Molecular chaperone DnaJ *Desulfacinum hydrothermale*
WP 073038211.1 Molecular chaperone DnaJ *Desulfacinum infernum*
0.2

GroL

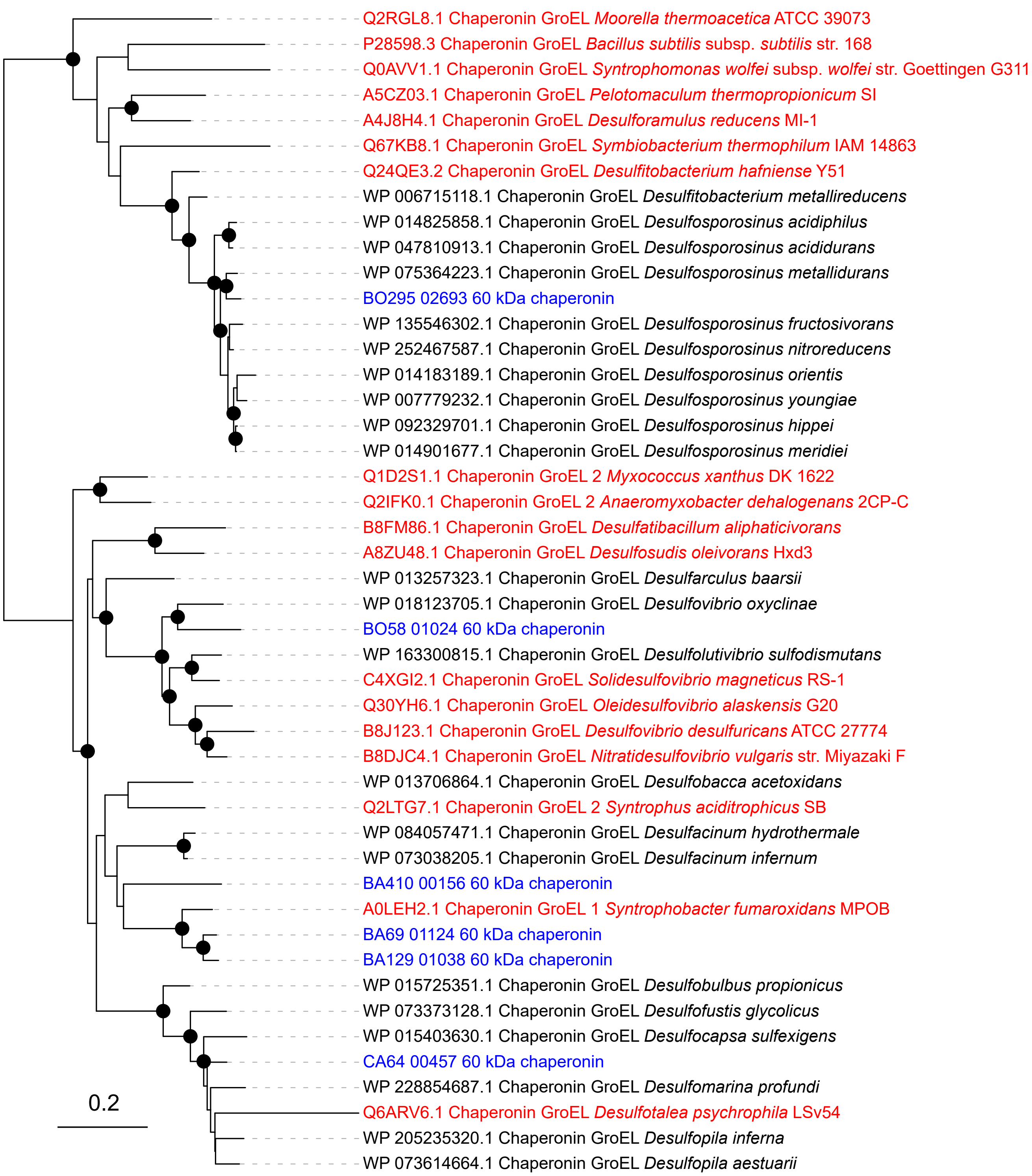
Q2RGL8.1 Chaperonin GroEL Moorella thermoacetica ATCC 39073
P28598.3 Chaperonin GroEL Bacillus subtilis subsp. subtilis str. 168
Q0AVV1.1 Chaperonin GroEL Syntrophomonas wolfei subsp. wolfei str. Goettingen G311
A5CZ03.1 Chaperonin GroEL Pelotomaculum thermopropionicum SI
A4J8H4.1 Chaperonin GroEL Desulforamulus reducens MI-1
Q67KB8.1 Chaperonin GroEL Symbiobacterium thermophilum IAM 14863
Q24QE3.2 Chaperonin GroEL Desulfitobacterium hafniense Y51
WP 006715118.1 Chaperonin GroEL Desulfitobacterium metallireducens
WP 014825858.1 Chaperonin GroEL Desulfosporosinus acidiphilus
WP 047810913.1 Chaperonin GroEL Desulfosporosinus acididurans
WP 075364223.1 Chaperonin GroEL Desulfosporosinus metallidurans
BO295 02693 60 kDa chaperonin
WP 135546302.1 Chaperonin GroEL Desulfosporosinus fructosivorans
WP 252467587.1 Chaperonin GroEL Desulfosporosinus nitroreducens
WP 014183189.1 Chaperonin GroEL Desulfosporosinus orientis
WP 007779232.1 Chaperonin GroEL Desulfosporosinus youngiae
WP 092329701.1 Chaperonin GroEL Desulfosporosinus hippei
WP 014901677.1 Chaperonin GroEL Desulfosporosinus meridiei
Q1D2S1.1 Chaperonin GroEL 2 Myxococcus xanthus DK 1622
Q2IFK0.1 Chaperonin GroEL 2 Anaeromyxobacter dehalogenans 2CP-C
B8FM86.1 Chaperonin GroEL Desulfatibacillum aliphaticivorans
A8ZU48.1 Chaperonin GroEL Desulfosudis oleivorans Hxd3
WP 013257323.1 Chaperonin GroEL Desulfarculus baarsii
WP 018123705.1 Chaperonin GroEL Desulfovibrio oxyclinae
BO58 01024 60 kDa chaperonin
WP 163300815.1 Chaperonin GroEL Desulfolutivibrio sulfodismutans
C4XGI2.1 Chaperonin GroEL Solidesulfovibrio magneticus RS-1
Q30YH6.1 Chaperonin GroEL Oleidesulfovibrio alaskensis G20
B8J123.1 Chaperonin GroEL Desulfovibrio desulfuricans ATCC 27774
B8DJC4.1 Chaperonin GroEL Nitratidesulfovibrio vulgaris str. Miyazaki F
WP 013706864.1 Chaperonin GroEL Desulfobacca acetoxidans
Q2LTG7.1 Chaperonin GroEL 2 Syntrophus aciditrophicus SB
WP 084057471.1 Chaperonin GroEL Desulfacinum hydrothermale
WP 073038205.1 Chaperonin GroEL Desulfacinum infernum
BA410 00156 60 kDa chaperonin
A0LEH2.1 Chaperonin GroEL 1 Syntrophobacter fumaroxidans MPOB
BA69 01124 60 kDa chaperonin
BA129 01038 60 kDa chaperonin
WP 015725351.1 Chaperonin GroEL Desulfobulbus propionicus
WP 073373128.1 Chaperonin GroEL Desulfofustis glycolicus
WP 015403630.1 Chaperonin GroEL Desulfocapsa sulfexigens
CA64 00457 60 kDa chaperonin
WP 228854687.1 Chaperonin GroEL Desulfomarina profundi
Q6ARV6.1 Chaperonin GroEL Desulfotalea psychrophila LSv54
WP 205235320.1 Chaperonin GroEL Desulfopila inferna
WP 073614664.1 Chaperonin GroEL Desulfopila aestuarii
0.2

GroS

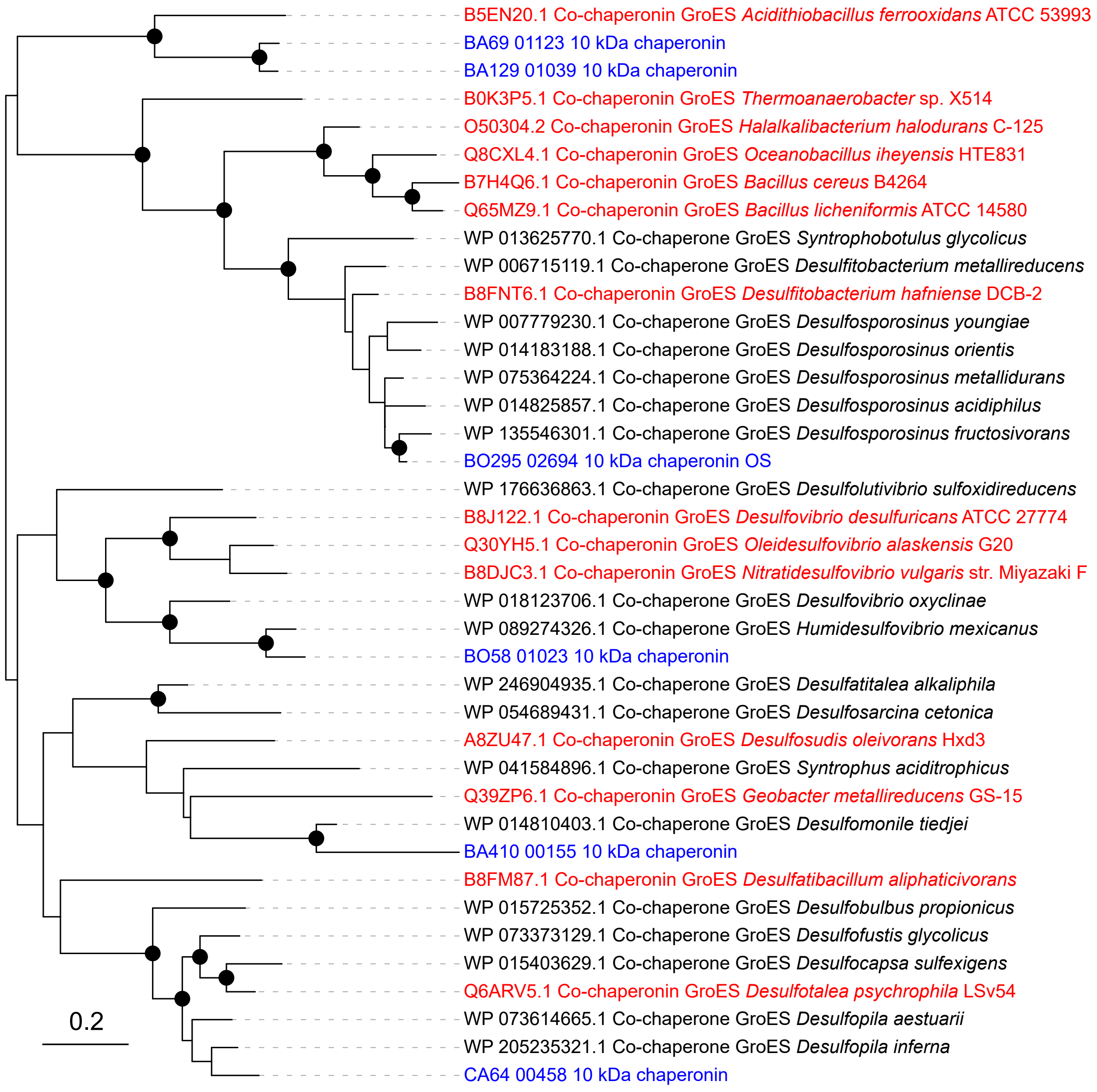
B5EN20.1 Co-chaperonin GroES Acidithiobacillus ferrooxidans ATCC 53993
BA69 01123 10 kDa chaperonin
BA129 01039 10 kDa chaperonin
B0K3P5.1 Co-chaperonin GroES Thermoanaerobacter sp. X514
O50304.2 Co-chaperonin GroES Halalkalibacterium halodurans C-125
Q8CXL4.1 Co-chaperonin GroES Oceanobacillus iheyensis HTE831
B7H4Q6.1 Co-chaperonin GroES Bacillus cereus B4264
Q65MZ9.1 Co-chaperonin GroES Bacillus licheniformis ATCC 14580
WP 013625770.1 Co-chaperone GroES Syntrophobotulus glycolicus
WP 006715119.1 Co-chaperone GroES Desulfitobacterium metallireducens
B8FNT6.1 Co-chaperonin GroES Desulfitobacterium hafniense DCB-2
WP 007779230.1 Co-chaperone GroES Desulfosporosinus youngiae
WP 014183188.1 Co-chaperone GroES Desulfosporosinus orientis
WP 075364224.1 Co-chaperone GroES Desulfosporosinus metallidurans
WP 014825857.1 Co-chaperone GroES Desulfosporosinus acidiphilus
WP 135546301.1 Co-chaperone GroES Desulfosporosinus fructosivorans
BO295 02694 10 kDa chaperonin OS
WP 176636863.1 Co-chaperone GroES Desulfolutivibrio sulfoxidireducens
B8J122.1 Co-chaperonin GroES Desulfovibrio desulfuricans ATCC 27774
Q30YH5.1 Co-chaperonin GroES Oleidesulfovibrio alaskensis G20
B8DJC3.1 Co-chaperonin GroES Nitratidesulfovibrio vulgaris str. Miyazaki F
WP 018123706.1 Co-chaperone GroES Desulfovibrio oxyclinae
WP 089274326.1 Co-chaperone GroES Humidesulfovibrio mexicanus
BO58 01023 10 kDa chaperonin
WP 246904935.1 Co-chaperone GroES Desulfatitalea alkaliphila
WP 054689431.1 Co-chaperone GroES Desulfosarcina cetonica
A8ZU47.1 Co-chaperonin GroES Desulfosudis oleivorans Hxd3
WP 041584896.1 Co-chaperone GroES Syntrophus aciditrophicus
Q39ZP6.1 Co-chaperonin GroES Geobacter metallireducens GS-15
WP 014810403.1 Co-chaperone GroES Desulfomonile tiedjei
BA410 00155 10 kDa chaperonin
B8FM87.1 Co-chaperonin GroES Desulfatibacillum aliphaticivorans
WP 015725352.1 Co-chaperone GroES Desulfobulbus propionicus
WP 073373129.1 Co-chaperone GroES Desulfofustis glycolicus
WP 015403629.1 Co-chaperone GroES Desulfocapsa sulfexigens
Q6ARV5.1 Co-chaperonin GroES Desulfotalea psychrophila LSv54
WP 073614665.1 Co-chaperone GroES Desulfopila aestuarii
WP 205235321.1 Co-chaperone GroES Desulfopila inferna
CA64 00458 10 kDa chaperonin
0.2

HtpG

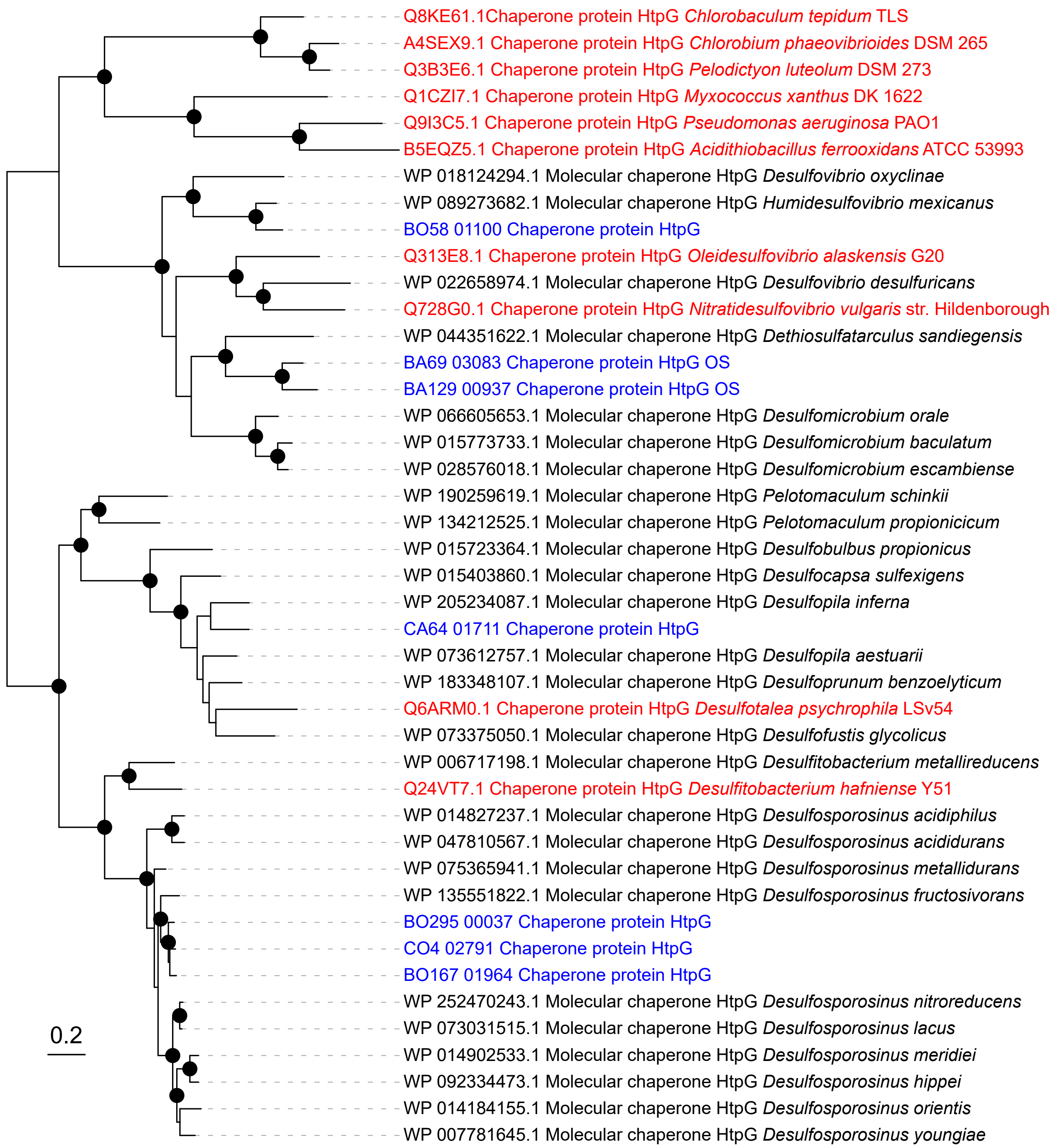

Q8KE61.1Chaperone protein HtpG Chlorobaculum tepidum TLS
A4SEX9.1 Chaperone protein HtpG Chlorobium phaeovibrioides DSM 265
Q3B3E6.1 Chaperone protein HtpG Pelodictyon luteolum DSM 273
Q1CZI7.1 Chaperone protein HtpG Myxococcus xanthus DK 1622
Q9I3C5.1 Chaperone protein HtpG Pseudomonas aeruginosa PAO1
B5EQZ5.1 Chaperone protein HtpG Acidithiobacillus ferrooxidans ATCC 53993
WP 018124294.1 Molecular chaperone HtpG Desulfovibrio oxyclinae
WP 089273682.1 Molecular chaperone HtpG Humidesulfovibrio mexicanus
BO58 01100 Chaperone protein HtpG
Q313E8.1 Chaperone protein HtpG Oleidesulfovibrio alaskensis G20
WP 022658974.1 Molecular chaperone HtpG Desulfovibrio desulfuricans
Q728G0.1 Chaperone protein HtpG Nitratidesulfovibrio vulgaris str. Hildenborough
WP 044351622.1 Molecular chaperone HtpG Dethiosulfatarculus sandiegensis
BA69 03083 Chaperone protein HtpG OS
BA129 00937 Chaperone protein HtpG OS
WP 066605653.1 Molecular chaperone HtpG Desulfomicrobium orale
WP 015773733.1 Molecular chaperone HtpG Desulfomicrobium baculatum
WP 028576018.1 Molecular chaperone HtpG Desulfomicrobium escambiense
WP 190259619.1 Molecular chaperone HtpG Pelotomaculum schinkii
WP 134212525.1 Molecular chaperone HtpG Pelotomaculum propionicicum
WP 015723364.1 Molecular chaperone HtpG Desulfobulbus propionicus
WP 015403860.1 Molecular chaperone HtpG Desulfocapsa sulfexigens
WP 205234087.1 Molecular chaperone HtpG Desulfopila inferna
CA64 01711 Chaperone protein HtpG
WP 073612757.1 Molecular chaperone HtpG Desulfopila aestuarii
WP 183348107.1 Molecular chaperone HtpG Desulfoprunum benzoelyticum
Q6ARM0.1 Chaperone protein HtpG Desulfotalea psychrophila LSv54
WP 073375050.1 Molecular chaperone HtpG Desulfofustis glycolicus
WP 006717198.1 Molecular chaperone HtpG Desulfitobacterium metallireducens
Q24VT7.1 Chaperone protein HtpG Desulfitobacterium hafniense Y51
WP 014827237.1 Molecular chaperone HtpG Desulfosporosinus acidiphilus
WP 047810567.1 Molecular chaperone HtpG Desulfosporosinus acididurans
WP 075365941.1 Molecular chaperone HtpG Desulfosporosinus metallidurans
WP 135551822.1 Molecular chaperone HtpG Desulfosporosinus fructosivorans
BO295 00037 Chaperone protein HtpG
CO4 02791 Chaperone protein HtpG
BO167 01964 Chaperone protein HtpG
WP 252470243.1 Molecular chaperone HtpG Desulfosporosinus nitroreducens
WP 073031515.1 Molecular chaperone HtpG Desulfosporosinus lacus
WP 014902533.1 Molecular chaperone HtpG Desulfosporosinus meridiei
WP 092334473.1 Molecular chaperone HtpG Desulfosporosinus hippei
WP 014184155.1 Molecular chaperone HtpG Desulfosporosinus orientis
WP 007781645.1 Molecular chaperone HtpG Desulfosporosinus youngiae
0.2
